# Genetic variation of clock genes and cancer risk: a field synopsis and meta-analysis

**DOI:** 10.18632/oncotarget.15074

**Published:** 2017-02-03

**Authors:** Clara Benna, Charlotte Helfrich-Förster, Senthilkumar Rajendran, Halenya Monticelli, Pierluigi Pilati, Donato Nitti, Simone Mocellin

**Affiliations:** ^1^ Department of Surgery Oncology and Gastroenterology, University of Padova, Padova, Italy; ^2^ Neurobiology and Genetics, Theodor-Boveri Institute, Biocenter, University of Würzburg, Würzburg, Germany; ^3^ Clinica Chirurgica I, Azienda Ospedaliera Padova, Padova, Italy; ^4^ Sant'Antonio Hospital, Padova, Italy; ^5^ Istituto Oncologico Veneto, IOV-IRCSS, Padova, Italy

**Keywords:** circadian rhythms, clock genes, SNP, meta-analysis, cancer risk

## Abstract

**BACKGROUND:**

The number of studies on the association between clock genes’ polymorphisms and cancer susceptibility has increased over the last years but the results are often conflicting and no comprehensive overview and quantitative summary of the evidence in this field is available.

**RESULTS:**

Literature search identified 27 eligible studies comprising 96756 subjects (cases: 38231) and investigating 687 polymorphisms involving 14 clock genes. Overall, 1025 primary and subgroup meta-analyses on 366 gene variants were performed. Study distribution by tumor was as follows: breast cancer (*n*=15), prostate cancer (*n*=3), pancreatic cancer (*n*=2), non-Hodgkin's lymphoma (*n*=2), glioma (*n*=1), chronic lymphocytic leukemia (*n*=1), colorectal cancer (*n*=1), non-small cell lung cancer (*n*=1) and ovarian cancer (*n*=1).

We identified 10 single nucleotide polymorphisms (SNPs) significantly associated with cancer risk: *NPAS2* rs10165970 (mixed and breast cancer shiftworkers), rs895520 (mixed), rs17024869 (breast) and rs7581886 (breast); CLOCK rs3749474 (breast) and rs11943456 (breast); RORA rs7164773 (breast and breast cancer postmenopausal), rs10519097 (breast); RORB rs7867494 (breast cancer postmenopausal), PER3 rs1012477 (breast cancer subgroups) and assessed the level of quality evidence to be intermediate. We also identified polymorphisms with lower quality statistically significant associations (*n*=30).

**CONCLUSIONS:**

Our work supports the hypothesis that genetic variation of clock genes might affect cancer risk. These findings also highlight the need for more efforts in this research field in order to fully establish the contribution of clock gene variants to the risk of developing cancer.

**METHODS:**

We conducted a systematic review and meta-analysis of the evidence on the association between clock genes’ germline variants and the risk of developing cancer. To assess result credibility, summary evidence was graded according to the Venice criteria and false positive report probability (FPRP) was calculated to further validate result noteworthiness. Subgroup meta-analysis was also performed based on participant features and tumor type. The breast cancer subgroup was further stratified by work conditions, estrogen receptor/progesterone receptor status and menopausal status, conditions associated with the risk of breast cancer in different studies.

## INTRODUCTION

Circadian rhythms (from Latin: *circa diem*) are biological processes which occur approximately every 24 hours. Sleep-wake cycles, cycling of body temperature, hormone secretion, heart rate, blood pressure, excretion and many other physiological parameters are all circadian phenomena. These circadian events are controlled by biological clocks, which are endogenous and self-sustained mechanisms that synchronize with both environmental cues, such as light and temperature, and with social cues, such as physical activity and feeding behavior [1]. The cogwheels of the circadian clock are proteins, whose production and degradation are controlled by interlocked feed-back loops [2]. At least 20% of all mammalian genes have been estimated to be clock-controlled, an indication of extensive circadian gene regulation [3, 4]. Sleep deprivation, jet-lag, shiftwork involving nightshifts and unnatural light exposure are all potential causes of circadian disruption which has been correlated, in different epidemiological studies and in laboratory studies, with diseases such as obesity, diabetes, depression and cancer [5–15]. In 2007, the International Agency for Research on Cancer (IARC) classified “shiftwork that involves circadian disruption” as a probable carcinogen (class 2a) [16].

So far, twelve core circadian genes, also known as clock genes, have been identified in humans: *CLOCK* (*clock circadian regulator*) [17], *CSNK1E* (casein kinase I epsilon) [18], *CRY1* (cryptochrome circadian clock 1), *CRY2* (cryptochrome circadian clock 2) [19], *PER1* (period circadian clock 1), *PER2* (period circadian clock 2), *PER3* (period circadian clock 3) [20, 21], *NPAS2* (neuronal PAS domain protein 2) [22], *ARNTL* (aryl hydrocarbon receptor nuclear translocator like, also referred to as brain and muscle Arnt-like protein-1, *BMAL1*) [23–25], *RORA* (RAR related orphan receptor A) [26], *NR1D1* (nuclear receptor subfamily 1 group D member 1 also known as Rev-Erb alpha) [27] and *NR1D2* (nuclear receptor subfamily 1 group D member 2 also known as Rev-Erb beta) [28–30]. An additional clock-related gene is *TIMELESS* (timeless circadian clock) [31].

These clock genes may affect cancer susceptibility by impacting on the biological pathways that regulate DNA damage and repair, carcinogen metabolism and/or detoxification, cell-cycle and apoptosis [32, 33]. Furthermore, innovative work in the field of molecular cancer epidemiology suggested that genetic variants in the clock genes are a potential risk factor for breast cancer [34]. In the first molecular epidemiologic study correlating a clock gene with the risk of human cancer, a structural variant in the circadian gene PER3 was detected to be significantly associated with increased risk of breast cancer [34, 35]. This clock–cancer link was confirmed in later studies which showed genetic associations between some variants of the clock genes *NPAS2* [36], *CRY2* [37] and *CLOCK* [38] and the risk of breast carcinoma, prostate carcinoma and Non-Hodgkin lymphoma.

Over the last few years, increasingly more studies were published on this topic. Many germline polymorphisms of clock genes have been proposed as biomarkers associated with cancer risk [39]. However, the results emerging from this growing literature are not always consistent and no systematic review has yet been published.

The aim of the present work is to fill this gap in the literature by presenting the first synopsis and meta-analysis of the available evidence in the field of DNA variation of the clock genes and the risk of cancer, including the interaction of polymorphisms with tumor type. The vast majority of the epidemiological studies included in this analysis focused on the identification of genetic biomarkers for female breast cancer susceptibility and the possible interactions with different social and personal conditions. In this work breast cancer interactions with clock gene polymorphisms were stratified by work conditions, menopausal status and tumor estrogen receptor/progesterone receptor expression.

## RESULTS

### Characteristics of eligible studies

**Figure 1:**
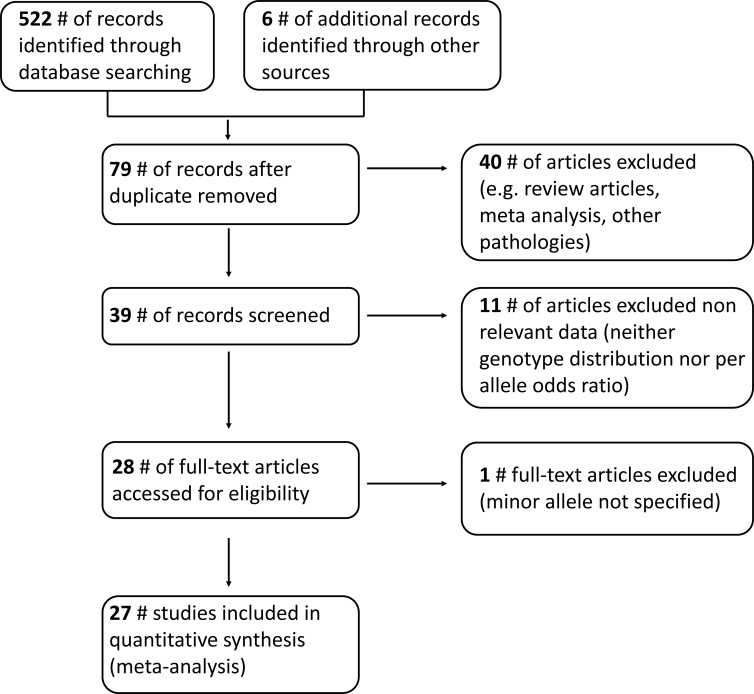
Flow diagram summarizing the study selection process

We identified 27 eligible articles (see Figure 1) comprising 96756 subjects (cases: 38231, range: 37–19159, mean: 1416), all being published after the year 2005. The main features and findings of all eligible studies are summarized in Table 1.

**Table 1:** Characteristics of the eligible studies included in this meta-analysis

INCLUDED ARTICLES		SUBJECTS CHARACTERISTICS					NEW CASTLE-OTTAWA QUALITY ASSESSMENT			
First Author	Year	Cancer Type	Cases	Controls	Ethnicity	Source of Controls	NOS1	NOS2	NOS3	NOS [0–9]
**Chu** [37]	2008	prostate	187	242	Asian	population	4	2	3	9
**Cotterchio** [49]	2015	pancreatic	179	566	Caucasian	population	4	1	3	8
**Couto** [50]	2014	NSCLC	78	74	Caucasian	population	4	2	3	9
**Dai** [51]	2011	breast	1538	1605	Asian	population	4	2	3	9
**Fu** [52]	2012	breast	441	479	Caucasian	hospital	3	2	3	8
**Grundy** [53]	2013	breast	953	974	Caucasian + Asian	population	4	2	3	9
**Hoffman** [38]	2010	breast	441	479	Caucasian	hospital	3	2	3	8
**Hoffman** [54]	2009	NHL	455	527	Caucasian	population	3	2	3	8
**Hoffman** [55]	2010	breast	441	479	Caucasian	hospital	3	2	3	8
**Hunter** [56]	2007	breast	1145	1142	Caucasian	population	4	2	3	9
**Jim** [57]	2015	ovarian	19159	33538	Caucasian	population	4	2	3	9
**Karantanos** [58]	2013	CRC	402	480	Caucasian	hospital	3	1	3	7
**Li** [59]	2011	breast	2702	5726	Caucasian	population	4	2	3	9
**Madden** [60]	2014	glioma	622	628	Caucasian	population	4	1	3	8
**Markt** [61]	2015	prostate	138	1214	Caucasian	population	4	2	3	9
**Monsees** [62]	2012	breast	609	1216	Caucasian	population	4	2	3	9
**Petersen** [63]	2010	pancreatic	3851	3934	Caucasian	population	4	2	3	9
**Rabstein** [64]	2014	breast	1022	1014	Caucasian	population	4	2	3	9
**Rana** [65]	2014	CLL	37	37	Caucasian	population	4	1	3	8
**Truong** [66]	2014	breast	1126	1174	Caucasian	population	4	2	3	9
**Wang** [115]	2011	breast	2145	2428	Asian + Caucasian	population	4	2	3	9
**Wirth** [68]	2014	breast	255	249	Caucasian	population	4	2	3	9
**Zhu** [69]	2008	breast	431	476	Caucasian	hospital	3	2	3	8
**Zhu** [35]	2005	breast	389	432	Caucasian	hospital	3	2	3	8
**Zhu** [70]	2009	prostate	1308	1266	Caucasian	population	4	2	3	9
**Zhu** [36]	2007	NHL	461	535	Caucasian	hospital	3	2	3	8
**Zienolddiny** [71]	2013	breast	563	619	Caucasian	population	3	2	3	8

According to the prevalent ancestry (the ethnicity of at least 80% of the enrolled subjects), 2 studies were Asian, 2 studies were mixed (Asian and Caucasian, analyzed separately) while 23 studies were Caucasian.

Based on the design, the majority of the studies were population-based case-control studies (n=20) and the remaining hospital-based case-control studies. Data from three GWAS were also available. Study distribution by tumor was as follows: breast cancer (n=15, cases=11354), prostate cancer (n=3, cases=1633), pancreatic cancer (n=2, cases=4030), non-Hodgkin's lymphoma (n=2, cases=916), glioma (n=1, cases=622), chronic lymphocytic leukemia (n=1, cases=37), colorectal cancer (n=1, cases=402), non-small cell lung cancer (n=1, cases=78) and ovarian cancer (n=1, cases=19159). In all studies, cancer diagnosis was confirmed by pathology evaluation. Moreover, controls were mainly matched on age, sex and ethnicity.

Less than one fourth (n=6) of the eligible studies specified subjects’ work conditions (daywork, nightwork or shiftwork with night shifts), while, among the breast cancer studies, only 6 out of 15 specified the ER/PR status (positive vs negative) of the primary tumor and 10 out of 15 the menopausal status of the subjects. For details on work conditions in the six eligible studies see Supplementary Table S1.

Overall, data on 687 polymorphisms involving the 14 selected clock genes were available (see online Supplementary Table S2). Variation consisted of SNP, except for 1 VNTR (variable number of tandem repeats) of the PER3 gene.

These genetic variants were located in the DNA upstream the “relevant” (meant as physically closest) gene (including the promoter region) (n=44), downstream the “relevant” gene (n=24), in introns (n=579), in exons (n=22), in the 5′-UTR (n=2) and the 3′-UTR (n=16). Among the exonic SNPs, the functional effects were generally missense (n=14) and synonymous coding changes (n=8).

### Meta-analysis findings

The results of data meta-analysis are comprehensively reported in the online Supplementary Table S3. At least two independent datasets were available for 366 variants across 14 genes, which allowed us to perform 1025 meta-analyses (see online Supplementary Table S2). Of these, 366 were primary meta-analyses and 659 meta-analyses of subgroups as defined by cancer type: breast cancer (n=291) vs prostate cancer (n=24); among breast cancer, work conditions: <2 years of shiftwork (n=46) vs >2 years of shiftwork (n=46) vs AnyShiftwork (n=12); ER/PR status: positive (n=15) vs negative (n=15) and menopausal status: premenopausal (n=11) vs postmenopausal (n=222).

The number of datasets meta-analyzed ranged from 2 to 14, the mean number being 3. Based on the number of datasets, the most studied variants were the following: *NPAS2* rs2305160 (n=14), *CRY2* rs1401417 (n=10), *ARNT*L rs7950226 (n=7), *NPAS2* rs17024926 (n=7), *CSNK1E* rs1534891 (n=7) and *NPAS2* rs1369481 (n=7).

The number of subjects (cases plus controls) enrolled in the 1025 meta-analyses ranged from 1927 to 58111 (median: 12372). Based on the number of subjects, the most studied variants were the following: *CRY2* rs10838527 (n=58111), *NPAS2* rs732375 (n=48514), *NPAS2* rs2305160 (n=32576), *ARNTL* rs3789327 (n=28173), *ARNTL* rs3816360 (n=27197) and *NPAS2* rs1369481 (n=25514).

Of the 1025 meta-analyses performed, 50 (5%) resulted nominally statistically significant (p<.05), whereas the remaining 975 did not reach statistical significance.

Among the statistically significant associations identified by meta-analysis, the level of summary evidence was intermediate in 13 (26%) and low in 37 analyses. No associations with high level of evidence were found. The insufficient magnitude of association (according to Venice criteria n.3, associations with summary OR <1.15 or >.87 in case of protective effect) and high FPRP (>.2) were the most frequent single cause of non-high-quality level of evidence. Among the intermediate-quality associations, FPRP was optimal at the 10E-2 level for 2/14 (*NPAS2* rs10165970 and rs895520). The details of significant associations characterized by intermediate level of summary evidence are reported in Table 2.

**Table 2:** Meta-analysis results: genetic variants significantly associated with cancer risk

Gene Symbol	SNP ID	Cancer Type	Subgroup	Data Sets	OR	LL	UL	I 2%	P Value	Cases	Controls	Risk Allele	Venice Criteria	Level of Evidence
CLOCK	rs11943456	mixed	Primary	3	1.11	1	1.2	0	.05	5605	6292	G	AAC	Low
CLOCK	rs11943456	breast	Overall	2	1.16	1.02	1.3	0	.02	1754	2358	G	AAA	Interm
CLOCK	rs3749474	breast	Overall	2	.86	.76	1	0	.02	1004	1098	T	AAA	Interm
CRY2	rs1401417	breast	>2y SW	2	.71	.54	.9	0	.01	327	459	G	BAA	Low
NPAS2	rs10165970	mixed	Primary	4	1.1	1.03	1.2	0	.002	8288	12050	A	AAC	Interm
NPAS2	rs10165970	breast	Overall	3	1.13	1.04	1.2	0	.003	4437	8116	A	AAC	Low
NPAS2	rs10165970	breast	<2y SW	2	1.19	1.03	1.4	0	.02	1235	1587	A	AAA	Interm
NPAS2	rs1053096	mixed	Primary	3	.93	.89	1	0	.007	6691	10874	T	AAC	Low
NPAS2	rs11674199	mixed	Primary	3	1.08	1.03	1.1	0	.002	6691	10874	A	AAC	Low
NPAS2	rs12622050	mixed	Primary	2	.94	.89	1	0	.04	6553	9660	A	AAC	Low
NPAS2	rs12712085	breast	Overall	2	.89	.81	1	0	.01	1735	2390	A	AAC	Low
NPAS2	rs12712085	breast	Post MP	2	.9	.81	1	0	.03	1821	1879	A	AAC	Low
NPAS2	rs1542178	mixed	Primary	4	.94	.89	1	0	.04	6260	7464	A	AAC	Low
NPAS2	rs1542178	breast	Overall	2	.9	.82	1	0	.05	2271	2316	A	AAC	Low
NPAS2	rs1542179	breast	Post MP	2	.88	.78	1	0	.04	1821	1879	C	AAC	Low
NPAS2	rs17024869	breast	Overall	2	.81	.7	.9	0	.006	2271	2316	G	AAA	Interm
NPAS2	rs17024869	breast	Post MP	2	.77	.6	1	54	.04	1821	1879	G	ACA	Low
NPAS2	rs3739008	mixed	Primary	5	1.06	1.01	1.1	0	.02	8405	9892	C	AAC	Low
NPAS2	rs3754677	mixed	Primary	3	.95	.9	1	0	.03	7836	12016	T	AAC	Low
NPAS2	rs3820787	mixed	Primary	3	.89	.81	1	0	.007	1873	3604	A	AAC	Low
NPAS2	rs3820787	breast	Overall	2	.88	.8	1	0	.005	1735	2390	A	AAC	Low
NPAS2	rs3820787	breast	<2y SW	2	.79	.63	1	63	.04	1235	1587	A	ACA	Low
NPAS2	rs4851384	mixed	Primary	3	.91	.83	1	0	.02	7679	10834	A	AAC	Low
NPAS2	rs7581886	breast	Overall	2	.86	.75	1	0	.03	1735	2390	G	AAA	Interm
NPAS2	rs895520	mixed	Primary	4	1.08	1.03	1.1	0	.001	7817	12048	A	AAC	Interm
NPAS2	rs895520	breast	Overall	2	1.09	1.02	1.2	0	.006	3828	6900	A	AAC	Low
NPAS2	rs935401	breast	Overall	2	1.11	1.02	1.2	0	.02	1735	2390	T	AAC	Low
PER1	rs2253820	breast	Primary	3	.89	.82	1	0	.004	4437	8116	A	AAC	Low
PER2	rs7602358	mixed	Overall	6	1.08	1.02	1.1	0	.005	8190	9358	G	AAC	Low
PER3	rs1012477	breast	Pre MP	2	.73	.59	.9	0	.005	641	712	G	AAA	Interm
PER3	rs1012477	breast	ER/PR +	2	.86	.75	1	0	.04	1771	1980	G	AAA	Interm
RORA	rs10162630	mixed	Primary	3	1.06	1	1.1	0	.04	6122	6250	G	AAC	Low
RORA	rs10519097	mixed	Primary	3	.91	.84	1	9	.02	6122	6250	A	AAC	Low
RORA	rs10519097	breast	Overall	2	.85	.75	1	0	.008	2271	2316	A	AAA	Interm
RORA	rs10519097	breast	Post MP	2	.87	.75	1	0	.04	1821	1879	A	AAC	Low
RORA	rs1632660	mixed	Primary	3	.94	.88	1	0	.05	6122	6250	A	AAC	Low
RORA	rs17270188	breast	Overall	2	1.12	1.02	1.2	0	.02	2271	2316	G	AAC	Low
RORA	rs2899666	breast	Primary	2	1.13	1	1.3	26	.04	2271	2316	G	ABA	Interm
RORA	rs339972	mixed	Primary	3	1.08	1.01	1.2	0	.02	6122	6250	G	AAC	Low
RORA	rs4774388	breast	Post MP	2	.87	.76	1	0	.05	1821	1879	G	AAC	Low
RORA	rs4775355	breast	Overall	2	.89	.79	1	0	.03	2271	2316	G	AAC	Low
RORA	rs4775355	breast	Post MP	2	.87	.76	1	0	.03	1821	1879	G	AAC	Low
RORA	rs7164773	breast	Overall	2	1.16	1.05	1.3	0	.003	2271	2316	A	AAA	Interm
RORA	rs7164773	breast	Post MP	2	1.17	1.04	1.3	0	.01	1821	1879	A	AAA	Interm
RORA	rs7172348	mixed	Primary	3	.93	.87	1	0	.03	6122	6250	G	AAC	Low
RORA	rs8024629	breast	Overall	2	.89	.8	1	0	.05	2271	2316	A	AAC	Low
RORB	rs10869417	breast	Post MP	2	.88	.77	1	0	.04	1821	1879	A	AAC	Low
RORB	rs7867494	breast	Overall	2	.9	.81	1	0	.04	2271	2316	G	AAC	Low
RORB	rs7867494	breast	Post MP	2	.86	.76	1	0	.01	1821	1879	G	AAA	Interm

Abbreviations: NSCLC non-small cell lung cancer; CRC colorectal cancer; CLL chronic lymphotic leukemia; NHL *non*-*Hodgkins lymphoma;* NOS1 selection; NOS2 comparability; NOS3 exposure.

OR: Odds Ratio; UL: 95% upper level; LL: 95% lower level; Venice Criteria: A (high), B (moderate), C (weak) credibility for three parameters (amount of evidence, heterogeneity and bias; see text for more details); Level of Evidence: overall level of summary evidence according to the Venice criteria; 2y SW: 2 years shiftwork; MP: menopausal; ER/PR: estrogen receptor/progesterone receptor; Interm: Intermediate

Statistically significant associations with intermediate level of evidence emerging from data meta-analysis are described below, listed by gene.

### NPAS2

*NPAS2* is the largest human clock gene. It maps on chromosome 2 at 2q11.2 and encodes for a member of the basic helix-loop-helix PAS class of transcription factors [72]. When dimerized with ARNTL (BMAL1), NPAS2 binds to E-box regulatory elements in target promoter regions and enhances target gene expression (Figure 3). Previous studies reported NPAS2 as a putative tumor suppressor [73].

A meta-analysis was possible for 64 *NPAS2* SNPs out of the 121 studied ones. Including sub-groups, 183 meta-analyses were performed and 24 of them (13%) were statistically significant (Supplementary Table S2).

rs10165970 is an intronic G>A SNP which showed a highly significant association with cancer in general according to the mixed primary meta-analysis (including 20338 subjects) although an intermediate level of evidence due to insufficient magnitude of association (summary OR: 1.1, CI: 1.03-1.17, P=.002), FPRP was low classifying this SNP as “noteworthy”. Upon subgroup meta-analysis, data from three datasets of breast cancer (including 12553 subjects) revealed that the association was statistically significant, but with a low level of evidence. In two of the three datasets subjects’ work conditions had been considered. There was a significant association with an intermediate level of evidence in the sub-group whose shiftwork lasted less than two years (2822 shiftworkers; summary OR: 1.19, CI: 1.03-1.37, P=.02). Noticeably, this SNP was not associated with breast cancer in shiftworkers whose job lasted more than two years (786 shiftworkers).

rs895520 is an intronic G>A SNP. The primary meta-analysis of four datasets (including 19865 subjects) revealed a highly significant association with an intermediate level of evidence due to insufficient magnitude of association. FPRP was low, classifying this SNP as “noteworthy” (summary OR: 1.08, CI: 1.03-1.13, P=.001). Upon the breast cancer subgroup, the statistically significant association had a low level of evidence.

rs17024869 is an intronic A>G SNP. Upon primary meta-analysis of three datasets (including 12372 subjects) the association of this SNP was statistically marginal with a high degree of heterogeneity. Instead, the meta-analysis of the breast cancer sub-group, including 4587 subjects, showed that the G allele was associated with a reduced risk with an intermediate level of evidence (summary OR: .81, CI: .7-.94, P=.006). The analysis was significant considering the postmenopausal subgroup (3700 subjects) but the level of evidence was low because of the high degree of heterogeneity.

rs7581886 is an intronic A>G SNP. The meta-analysis was possible only in the breast cancer subgroup and comprised two datasets (4125 subjects). The G allele resulted significantly associated with a reduced risk of breast cancer (summary OR: .86, CI: .75-.98, P=.03). Interestingly, the association of this SNP in the shift-work subgroup was statistically marginal with a high degree of heterogeneity.

### CLOCK

*CLOCK* maps on chromosome 4 at 4q12. Like for NPAS2, its corresponding protein product belongs to the basic helix-loop-helix PAS family of transcription factors and forms heterodimers with ARNTL (BMAL1) to enhance target gene expression (Figure 3). CLOCK is also involved in growth arrest, DNA repair and apoptosis upon genotoxic stress caused by UV radiation, suggesting that this molecule may represent an important “caretaker” promoting cell cycle arrest upon DNA damage [74]. CLOCK has the properties of a histone acetyl transferase and is involved in chromatin remodeling [75].

A meta-analysis was possible for 17 *CLOCK* SNPs out of the 41 studied ones. Including sub-groups, 47 meta-analysis were performed and 3 (6%) were significant (Supplementary Table S2).

rs3749474 C>T SNP is located on 3′-UTR region of *CLOCK*. We could perform a meta-analysis only in the breast cancer subgroup, employing two datasets (2102 subjects). The minor allele (T) resulted significantly associated with a decreased risk of developing breast cancer (summary OR: .86, CI: .76-.98, P=.02).

rs11943456 is a A>G SNP located 17kb downstream the 3′-UTR region of *CLOCK* in the first intron of *TMEM165*, which codes for a transmembrane protein, partially overlapping with *CLOCK* and transcribed on the opposite strand. TMEM165 is suggested to be involved in congenital disorders of glycosylation (CDG) [76], but, so far, no connection with cancer risk has been investigated. Upon primary meta-analysis of the three datasets comprising 11897 subjects, the association of this SNP was statistically borderline with a low level of evidence, while in the breast cancer subgroup, including 4112 subjects, the association was significant (summary OR: 1.16, CI: 1.2-1.32, P=.02).

### Retinoid-related orphan receptors (RORs)

These genes regulate the expression of several components of the circadian clock and may play a role in integrating the circadian clock and the rhythmic pattern of expression of downstream genes (for an exhaustive review see [77]). ARNTL (BMAL1)-CLOCK or ARNTL (BMAL1)-NPAS2 heterodimers promote the transcription of *RORs*, such as *RORA* and *RORB*, which in turn activate the transcription of *ARNTL* (Figure 3) [78]. Moreover, this family of proteins regulates various cellular and pathological activities [79, 80]. As a result of alternative promoter usage and exon splicing, each *ROR* gene generates several isoforms that differ only in their amino-terminus [77]. Most isoforms exhibit a distinct pattern of tissue-specific expression and are involved in the regulation of different physiological processes and target genes. The ROR genes encode 459 to 556 amino acid long proteins. RORs exhibit a typical nuclear receptor domain structure consisting of four major functional domains: an N-terminal (A/B) domain followed by a highly conserved DNA-binding domain (DBD), a hinge domain, and a C-terminal ligand-binding domain. RORB shares high sequence homology to RORA and is strongly expressed in the suprachiasmatic nucleus, in the pineal gland and in the retina which are the major regions responsible for the regulation of circadian rhythm, this may suggest similar involvement in the circadian pathway although there is little evidence supporting the regulatory effects on clock genes [81].

### RORA

*RORA* maps on chromosome 15 at 15q21-q22, spans a 730 kb large genomic region comprised of 15 exons and encodes for one member of the retinoid orphan nuclear receptor subfamily of orphan receptors. RORA has been reported as potential tumor suppressor [82, 83].

Meta-analysis was possible for 151 *RORA* SNPs out of the 289 studied ones. Including sub-groups, 429 meta-analyses were performed and 15 (4%) were statistically significant (Supplementary Table S2).

rs7164773 is an intronic G>A SNP. Upon primary meta-analysis of three datasets (including 12372 subjects) the association was not significant and displayed a high level of heterogeneity. Interestingly, subgroup meta-analysis (two datasets including 4587 subjects) showed that the A allele was associated with an increased risk of developing breast cancer (summary OR: 1.16, CI: 1.05-1.29, P=.003). This variant was also associated with disease risk (3700 subjects) in postmenopausal patients with breast cancer (summary OR: 1.17, CI: 1.04-1.32, P=.01). The level of evidence was intermediate in both analyses due to a high (>.2) FPRP.

rs10519097 is an intronic G>A SNP. Upon primary meta-analysis of three datasets (including 12372 subjects) the association was significant, although the level of evidence was low due to an insufficient magnitude of association. Noticeably, subgroups meta-analysis employing two datasets (4587 subjects) showed that the association of this SNP with breast cancer was significant with an intermediate level of evidence (summary OR: .85, CI: .75-.96, P=.008). The A allele was associated with a reduced risk of developing cancer also in postmenopausal breast cancer patients (3700 subjects; summary OR: .87, CI: .75-1, P=.04), but the level of this evidence resulted low.

### RORB

This gene maps to 9q21.13 and covers approximately 188kb of genomic DNA. RORB was found to be overexpressed in primary uterine leiomyosarcoma [81, 84], but also down-regulated in both serous and endometrioid cancer [85]. The expression levels of RORB in other tumors as well as the molecular mechanisms of how RORB affects tumor formation and progression are still unknown.

A meta-analysis was possible for 19 *RORB* SNPs out of the 35 studied ones. Including sub-groups, 53 meta-analyses were performed and 3 of them (6%) were significant (Supplementary Table S2).

rs7867494 is an intronic A>G SNP. Upon primary meta-analysis of three datasets (including 12372 subjects) the association was not significant with a high level of heterogeneity. Noticeably, subgroups meta-analysis showed that the association of this SNP with breast cancer employing two datasets (4587 subjects) was significant although the level of evidence was low (summary OR: .9, CI: .81-1, P=.04). The G allele was associated with a reduced risk of developing cancer also in postmenopausal breast cancer patients. Moreover the level of evidence was intermediate (3700 subjects; summary OR: .86, CI: .76-.97, P=.01).

### PERs (PER1, PER2, PER3)

*PERs* code for PAS domain-containing key regulators of the circadian clock. Many of the core circadian rhythm PAS factors, including the period (*PER*) genes, are downregulated in breast, colorectal, prostate, glioma and non-small cell lung cancer in humans; moreover, it has been suggested that PER1 and PER2 function as tumor suppressors [86–91]. *PER* genes control their own transcription by directly repressing ARNTL (BMAL1) heterodimers, their activators (Figure 3) [72]. *PER1* maps on chromosome 17 at 17p13.1, *PER2* on chromosome 2 at 2q37.3 and *PER3* on chromosome 1 at 1p36.23.

A meta-analysis was possible for 10, 10 and 24 *PERs* SNPs out of the respectively 14, 20, 41 studied ones. Including sub-groups 24, 33, 63 meta-analyses were performed and 1 (4%), 1 (3%), 2 (3%) were statistically significant (Supplementary Table S2). rs2253820 in *PER1* a synonymous G>A SNP had low level of evidence in breast cancer sub-group, as well as rs7602358 T>G downstream *PER2*.

rs1012477 in *PER3* is an intronic C>G SNP. Upon primary meta-analysis of six datasets comprising 10585 subjects, the association was not significant with a medium level of heterogeneity. Among breast cancer subgroups this SNP was associated with reduced risk in premenopausal women (1353 subjects; summary OR: .73, CI: .59-.91, P=.005) and in ER/PR positive subgroup (3176 subjects; summary OR: .86, CI: .75-.99, P=.04) with an intermediate level of evidence.

### CRYs (CRY1, CRY2)

CRY proteins contain a conserved photolyase homology region (PHR), which binds the cofactor FAD (flavine adenine dinucleotide), consisting of an N-terminal alphabeta-domain and a C-terminal all-helical domain as well as variable C-terminal extensions. CRYs form a complex with PERs and are involved in transcriptional repression of the ARNTL (BMAL1)/CLOCK heterodimers (Figure 3) [2]. The SNPs or deregulation of CRY1 and/or 2 are associated with increased susceptibility and mortality to several type of cancer [39]. *CRY1* maps on chromosome 12 at 12q23-q24.1 while *CRY2* on chromosome 11 at 11p11.2.

A meta-analysis was possible for 13 *CRY2* SNPs out of the 21 studied ones. Including sub-groups, 40 meta-analyses were performed and 1 of them (2.5 %) was significant (Supplementary Table S2). rs1401417 is an intronic C>G SNP. Upon primary meta-analysis of ten datasets (including 14834 subjects) the association was not significant with a medium level of heterogeneity. Being the second most studied SNP, in terms of the number of datasets, it was possible to analyze this variant in all the considered subgroups. The association with breast cancer resulted significant in shiftworkers with more than two years of shiftwork. The G allele was associated to a sensible decreased risk of developing breast cancer in “long term” shiftworkers, although the level of evidence was low due to the number of subjects enrolled (786 subjects; summary OR: .71, CI: .54-.93, P=.01).

Primary or subgroup meta-analysis for the SNPs concerning all the other clock genes (*ARNTL*, *CRY1*, *CSNK1E*, *NR1D1*, *TIMELESS*) considered in this study were found to be not statistically significantly associated with cancer risk (Supplementary Table S3). It was not possible to perform any meta-analysis for *NR1D2* SNPs due to the lack of datasets.

## DISCUSSION

The potential relationship between the genetic variations of clock genes and cancer risk has only recently been investigated. In the present article we describe the results of the first synopsis and meta-analysis with evaluation of the quality of the cumulative evidence in this field.

Out of 366 variants investigated, we found that 10 polymorphisms in 7 genes were associated with susceptibility to cancer in general or to susceptibility of specific subgroups with an intermediate level of evidence. Subgroups in breast cancer primary tumor were identified by work conditions (<2 years of shiftwork vs >2 years of shiftwork vs any shiftwork), ER/PR status (ER or PR positive vs ER or PR negative) and menopausal status (premenopausal vs postmenopausal). The genes that contributed the most to the overall association with cancer risk were *NPAS2*, *CLOCK* and *RORs* (*RORA* and *RORB*).

### Circadian clock mechanism and SNPs

The genes considered in the present study are cardinal components of the circadian system and may play a role in carcinogenesis [5, 92]. The molecular oscillator is based on interlocked feedback loops within an activating unit (CLOCK, NPAS2, and ARNTL (BMAL1)) and a repressing unit (PER and CRY). The heterodimer complex, formed of ARNTL (BMAL1) and either of the two related proteins CLOCK or NPAS2, activates the transcription of *PERs* (*PER1*, *PER2*, and *PER3*) and *CRYs* (*CRY1* and *CRY2*) genes. In turn, PER and CRY repress their own transcription by acting directly on the ARNTL (BMAL1)–CLOCK/NPAS2 complex. ARNTL (BMAL1)–CLOCK/NPAS2 heterodimers also induce a secondary regulatory loop which activates the transcription of retinoic acid-related orphan nuclear receptors, *NR1D1, NR1D2*, *RORA* and *RORB*. RORA activates the transcription of ARNTL (BMAL), whereas NR1D1/D2 represses it [2] (see Figure 3 for a schematic description of the circadian clock mechanism in mammals). TIMELESS, a member of an evolutionary conserved family of ortologs [93, 94], is involved not only in circadian rhythmicity, interacting directly with CRY1 [95], but also in embryonic development, cell cycle progression, DNA replication, and the DNA damage response [96].

Rhythmic phenotypes are due to the clock-controlled expression of downstream genes with various biological functions, including some that are relevant for carcinogenesis such as cell cycle control [38], DNA damage response [73], chromatin remodeling [75, 97, 98] and metabolism control [99, 100].

NPAS2 and CLOCK share significant sequence homology which allows them to heterodimerize with ARNTL (BMAL1) and serve as a transcriptional enhancer in the positive circadian feedback loop regulating circadian rhythm. In the present study, *NPAS2* and *CLOCK* SNPs were the most frequently associated variants with cancer risk (13% and 6% respectively of the meta-analyses showed a statistically significant association), while their counterpart *ARNTL* was not (no association among 76 meta-analyses). Of note, it was not possible to analyze the *ARNTL* variant rs117104877, which is highly significantly associated with ovarian cancer risk in the study published by Jim and Colleagues [57], because of the scarcity of datasets. RORA and RORB are also transcription factors which share high sequence homology, with 4% and 6% of their studied variants showing a statistically significant association with cancer risk, respectively.

Fewer SNPs were studied in the eligible articles involving the repressing branch (PER and CRY) of the clock; nevertheless one association was found with *CRY* SNPs and only few with *PER* SNPs. Our data suggest that variation in the transcription factors of the positive limb of the clock might play a predominant role in cancer susceptibility.

Since the majority of the abovementioned SNPs is in intronic regions, there is no obvious explanation concerning their functions, as well as the reasons why they result to be protective or to increase cancer risk, while for those which are localized in the 3′UTR, it could be argued that a particular allele can affect the regulation of transcription, although the effects are still unknown.

### Shiftwork and breast cancer

Short sleep duration and repeated unnatural exposure to light at night, as it occurs with jet-lag, shiftwork with night shifts and nightwork, which are potential causes of circadian disruption, have been associated to cancer risk [16]. It has been hypothesized that this is due to the lack of synchronization of the endogenous clock with environmental cues [34]. Epidemiologic studies suggest that disrupting circadian rhythms might increase cancer risk, which appears to be especially true for breast cancer in night and rotating female shiftworkers [101]. Although most evidence to date regards the relationship between circadian disruption and breast cancer, there is also growing evidence on colon cancer. Two epidemiological studies have reported significantly elevated risk of colon cancer in shift working women [102] and men [103]. Prostate cancer risk may also be affected by circadian disruption for reasons similar to those listed for breast cancer [70]. The role of common variants in the clock genes of rotating night shiftworkers has been investigated in six different studies, five of which focused on breast cancer risk [53, 62, 64, 66, 71] and one studying *chronic lymphocytic leukemia* [65]. Most studies divide shiftworkers in <2 years of shiftwork and >2 years of shiftwork. Interactions highlighted between shiftwork and specific clock gene variants in relation to breast cancer were never replicated in the different studies. In our meta-analysis, two *NPAS2* variants, rs10165970 and rs3820787, showed a statistically significant association with breast cancer in shiftworkers (<2 years of shiftwork stratification), the former with an intermediate level of evidence whereas the latter with a low level of evidence. The G allele of *CRY2* rs1401417 appeared to have a protective effect against breast cancer in “long term” shiftworkers (>2 years of shiftwork stratification). Although the level of evidence was low due to the low number of subjects enrolled, this association appears to deserve further investigation. Zienolddiny and co-workers studied shiftwork with a paradigm based on work intensity and duration, and reported a protective effect of *CRY2* rs1401417 in women with four or more consecutive night shifts per month [71]. We could not add this dataset to >2 years shift work subgrouping, to increase the number of subjects, because of the incompatibility of the two shiftwork paradigms.

Of note, we were unable to analyze all the variants which showed interaction with shiftwork highlighted in different studies due to the scarcity of data and to different shiftwork paradigms used in different articles.

Chronotype represents the preference of an individual to perform activity in relation to the time of the day and night, it has genetic bases and may influence the adaptability to different work schedules [104, 105]. The circadian system regulates such behavioral manifestations and clock genes polymorphisms have been associated to chronotype differences. Both chronotype and clock genes polymorphisms have been associated individually with cancer risk in “circadian disruption” conditions [106–109]. So far the abovementioned three conditions have not been associated with cancer risk in the same population therefore this topic is worth further investigation.

### Menopausal status, hormone receptor status and breast cancer

Ten out of fifteen studies on breast cancer stratified datasets by menopausal status, and six by hormonal receptor status. Upon meta-analysis, ten variants out of 222 showed a statistically significant interaction between breast cancer risk and menopausal status, although only three with intermediate level of evidence involving *RORA* (rs7164773, post-menopause), *RORB* (rs7867494, post-menopause) and *PER3* (rs1012477, pre-menopause). Noticeably, the available data on the *PER3* rs1012477 variant also revealed a significant interaction between breast cancer risk and ER/PR status (ER/PR positive). Due to the scarcity of data in this stratification, we could not analyze all the variants which showed interaction with menopausal status included in different studies.

Some investigators have suggested that hormones, including estrogens, may influence the expression of clock genes [75, 91] and that, *vice versa*, clock genes may play a role in hormone regulation [110]. Nevertheless, the exact mechanisms underlying this interplay are largely unknown. NR1D1 circadian transcription factor has found to be co-amplified with the receptor *ERBB2* in “HER2-positive breast cancer” and probably contribute to the aggressiveness of this malignancy [111–113]. HER2 is regulated by ER, and the cross talk between this two proteins has been implicated in breast cancer etiology and drug resistance [114]. Two datasets included in this meta-analysis studied NR1D1 SNPs [53, 66] in ER positive and negative breast cancer patients, but they fail to find any statistically significant association with cancer risk. A meta-analysis of those SNPs was not possible, because the two datasets included different polymorphisms.

*CRY2* variant rs1041417 was strongly associated with premenopausal and with ER-positive in breast cancer patients by Dai and colleagues [51] employing a dataset of Asian ethnicity. Primary meta-analysis revealed a non-significant association, with a high level of between-study heterogeneity. This association might be specific of Asian ethnicity, as sensitivity analysis showed no heterogeneity when only considering Caucasian ancestry datasets. More studies are needed to clarify the role of rs1041417 related to an increase of breast cancer risk in the Asian population.

### Previous meta-analysis: NPAS2, PER3

Wang and Colleagues performed a meta-analysis [115] on the *NPAS2* most investigated missense polymorphism rs2305160, whose association with cancer risk has been assessed with conflicting results in different studies. The authors found a statistically significant association with cancer risk in general (AA+GA vs GG and AG vs GG models; studies, n=8; subjects, n=8382) and with breast cancer (A vs G, AA+GA vs GG and AG vs GG models; studies, n=3; subjects, n=3342), but none with prostate cancer (studies, n=2; subjects n=2916). In our meta-analysis, which included fourteen studies (two of which were GWAS) and 32576 participants, we found no evidence of association with cancer risk (OR: .96, CI: .91-1.01, P=.08). Stratified analysis by cancer type showed no statistically significant association with either breast (studies, n=7; subjects, n=16934; OR: .95, CI: .90-1.01, P=.1) or prostate cancer (studies, n=3; subjects, n=3926; OR: 1.02, CI: .92-1.13, p=.75).

Wang and Colleagues also investigated rs17024926, the second most studied *NPAS2* SNP, but failed to find significant association with cancer, independently of the genetic model (including 3 datasets). These findings have been confirmed by our meta-analysis, which included seven datasets.

Geng and Colleagues [116] focused on *PER3* polymorphisms: rs1012477 SNP and rs57875989, known as 4/5 repeats VNTR (the first PER3 polymorphism studied in relation to breast cancer risk), reporting no evidence of statistically significant association with cancer risk. In our meta-analysis on rs1012477 SNP, we could rely upon two additional datasets, one for prostate cancer [61] and one for breast cancer [66] for a total of six datasets (including 10585 subjects). Upon primary meta-analysis, the association was not statistically significant and had a medium level of heterogeneity, which confirms Geng's findings. As discussed previously, among breast cancer subgroups, the rs1012477 SNP is associated with reduced risk in premenopausal women and in ER/PR positive subgroup with an intermediate level of evidence.

Regarding *PER3* (rs57875989) 4/5 repeats VNTR, we did not find a significant association with cancer risk in any of the considered subgroups. Our analysis strengthens the evidence for lack of association between this polymorphism and cancer risk, as already suggested by Geng and Colleagues [116], who analyzed a lower number of studies, having added an additional dataset of breast cancer (Wirth et al., 2014).

### Strengths and limitations of the study

Our meta-analysis is the first to comprehensively cover clock genes germline variants thus far reported in relation with predisposition to cancer. Previous meta-analyses on this topic [115, 116] only included two SNPs each, in *PER3* and *NPAS2* clock genes, whereas we analyzed 366 variants across 14 genes in the circadian rhythm pathway. Our findings are based on an overall sample size and regard a number of variants much larger than those reported in any other previously published work in this field. Moreover, the present work is characterized not only by a systematic search of the international literature available in this field of investigation, but also by the effort to grade the quality of the pooled evidence according to criteria dedicated to molecular association studies, that is the Venice criteria. Finally, for each statistically significant meta-analysis, we calculated the FPRP to further inform readers on the reliability of the results we presented [117].

Nevertheless, we also recognize the limitations of this synopsis. In general, we considered core clock genes polymorphisms, but the relationship between genetic susceptibility to “circadian disruption” and cancer risk may occur via different clock-related pathways. Clock controlled-genes SNPs have been studied only recently, but the data are still insufficient to pool them together. Moreover, each polymorphism was analyzed independently while it is likely that the haplotype pattern of an individual may be associated with disease risk or *vice versa* may be protective regarding a particular disease state. Epigenetic modifications such as promoter methylation status was not assessed in our study. Hoffman and Colleagues in one of the studies included in this analysis [38] address this issue finding that hypermethylation in *CLOCK* gene promoter reduces the risk of breast cancer.

Another limitation concerns the shiftwork stratification. The division of shiftworker in three categories: < 2 years of shiftwork vs < 2 years of shiftwork vs any shiftwork was the only possible, given the structure of the papers included. Still the shiftwork definition varies from study to study (see Supplementary Table S1) and so far it is unclear whether and in which measure these differences in shiftwork paradigm definition influence the association with cancer risk and the interaction with germline polymorphisms.

We magnified under the meta-analysis lens one of the aspects of the interaction between circadian disruption and cancer susceptibility, nevertheless far more efforts are needed to unveil this fine relationship.

As regards the meta-analysis, its limitations are the following: first, some data sources have been used in more than one meta-analysis, which might lead to type I error inflation. However, since we implemented the FPRP method, this occurrence should be restrained. Second, only one genetic model (that is, the additive model) was used, whereas neither the recessive nor the dominant model were explored: however, our aim was not to identify the best genetic model for specific and already well-established polymorphisms but rather to summarize (in a quantitative fashion) the evidence regarding hundreds of genetic variants, a task that is best accomplished by adopting a conservative approach (i.e., the additive model). Moreover, genotype data were lacking for a large proportion of genetic variants, which does not allow to investigate other genetic models. Finally, testing different models in both primary and subgroup meta-analyses would have roughly tripled the number of tests to be performed, which would have led to a significant inflation of type I error [117].

In conclusion, the present work provides readers with the fist systematic review and quantitative summary of the available evidence on the association between genetic variation of clock-related genes and cancer risk. Our findings support this relationship and might become a useful informative platform for future investigation, which is certainly needed to shed more light in this promising field of cancer predisposition.

## MATERIALS AND METHODS

### Search strategy, eligibility criteria and data extraction

We followed the principles proposed by the Human Genome Epidemiology Network (HuGeNet) for the systematic review of molecular association studies [40–42].

A two-step search strategy was adopted. First, a systematic review of original articles, reviews and meta-analyses studying the association between any genetic variant of the 12 clock genes with established cardinal roles in circadian regulation (plus one clock-related gene, *TIMELESS* and one homolog, *RORB*) [39] and cancer risk was performed by searching MEDLINE via the PubMed gateway. The search was carried out using three groups of keywords: (1) “cancer”, “tumor”, “carcinoma”, “leukemia”, “lymphoma”, “melanoma” and “sarcoma”; (2) “clock” and “circadian”; (3) “polymorphism”, “single nucleotide polymorphism”, its acronym “SNP” and “variant”. Searches were conducted using all combinations of at least one keyword from each group. The search was then repeated gene by gene, adding the name (or the symbol) of each single clock gene to the words “polymorphism” or “SNP” or “variants”. In the second phase, references reported in the articles retrieved in the first phase were screened. Finally, given the increasing use of genome-wide association studies (GWAS), we also searched for publicly available data from this type of source.

Authors were contacted whenever unreported data were potentially useful to enable the inclusion of a study into the systematic review or to rule out data published in different articles but regarding overlapping series of datasets.

For analysis purposes, the search database was frozen in December 2015.

To be eligible the articles had to report: (1) results from case-control or cohort studies conducted in humans; (2) the association between germline variants of clock genes and the risk of any type of malignancy; (3) measures of association (odds ratios [OR] or relative risks [RR]) along with their 95% confidence intervals (CIs) or the raw data necessary to calculate them.

The quality of the studies was evaluated according to Newcastle-Ottawa quality assessment scale [43]. In brief, three parameters were evaluated with a “star system”: the selection of the study groups (0 to 4 “stars”), the comparability of the groups (0 to 2 “stars”) and the ascertainment of either the exposure or outcome of interest for case-control or cohort studies respectively (0 to 3 “stars”). A questionnaire with multiple choices was associated with each parameter to define the number of stars. The maximum total score was 9 “stars” and represented the highest quality.

The following data were extracted from eligible studies: authors’ names; region/country where the study was conducted; year of publication; numbers of cases and controls; prevalent ethnicity (>80%, categorized in Caucasian, Asian, African and mixed); allelic frequency in both cases and controls (if no raw data were available, summary data were collected, i.e. odds ratios and confidence intervals); study design (population-based versus hospital-based); genotyping method; cancer type; work conditions (daywork, nightwork or shiftwork); for breast cancer cases only, hormonal (ER/PR) and menopausal status.

### Selected clock genes

The selected clock genes are: *ARNTL, CLOCK*, *CRY1*, *CRY2*, *CSNK1E*, *NPAS2*, *NR1D1, NR1D2*, *PER1*, *PER2*, *PER3*, *RORA*, *RORB* and *TIMELESS*. The gene symbols used are those recommended by the Human Genome Organization (HUGO) Gene Nomenclature Committee (HGNC, http://www.genenames.org).

Both intra-gene and inter-gene variants were investigated. Concerning the inter-gene variants, the nearest gene was considered to be the relevant gene exclusively for classification purposes given that the functional effects of many tested polymorphisms remain unknown.

### Statistical analysis

We used odds ratios (ORs) and their corresponding 95% confidence intervals to measure the strength of association between each polymorphism and cancer risk. We calculated per allele ORs assuming an additive (co-dominant) genetic model. We chose this approach based on the following considerations: (1) many studies (including GWAS) report exclusively per-allele ORs; (2) the additive model can be considered a conservative choice between recessive and dominant models; (3) choosing one model (additive) avoids adjustment for multiple hypotheses testing, which is instead required when multiple models are tested; (4) methods allowing the data to suggest the most appropriate genetic model (such as the model-free approach [44]) require information on genotype distributions, which is not available for all the studies we included; (5) when a large sample size is available (as often occurs in systematic reviews), non-additive models (e.g., recessive and dominant models) rarely add high-quality information to the findings based on the additive model: this is the reason why the additive model is widely used in synopses of genetic association studies [45–47].

Random effects meta-analysis (inverse variance method) was used to calculate summary ORs; this model reduces to a fixed effect meta-analysis if between-study heterogeneity is absent. We chose this model mainly because of the large between-study heterogeneity usually expected in genetic association studies. A meta-analysis was performed only if at least two independent data sources were available. In case of GWAS, we considered discovery and validation phases as separate data sources.

**Figure 2:**
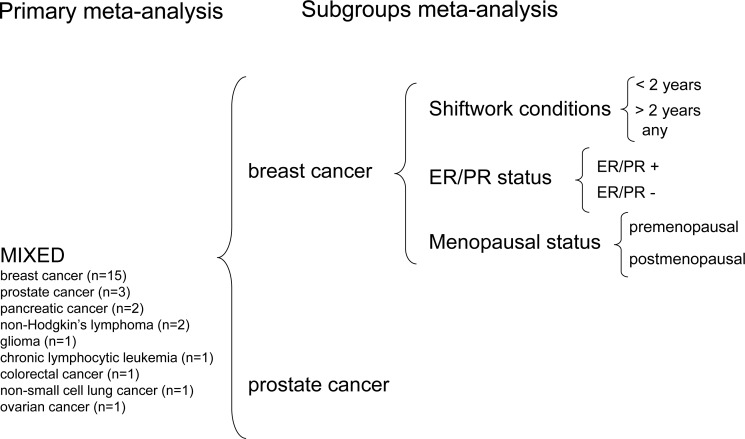
Flow diagram summarizing the study selection process

Subgroup analysis by primary tumor type (breast vs prostate), work conditions (<2 years Shiftwork vs >2 years Shiftwork vs AnyShiftwork), ER/PR status (positive vs negative) and menopausal status (premenopausal vs postmenopausal) was performed if data permitted (Figure 2). Allele frequency can vary significantly in a population, depending on ethnicity. It was not possible to perform a subgroup analysis by ethnicity (Asian vs Caucasian), due to the lack of sufficient Asian datasets, therefore in order to test any dominant study driving effect, we performed a sensitivity analysis by ethnicity excluding the Asian population-based studies from the analyses.

**Figure 3:**
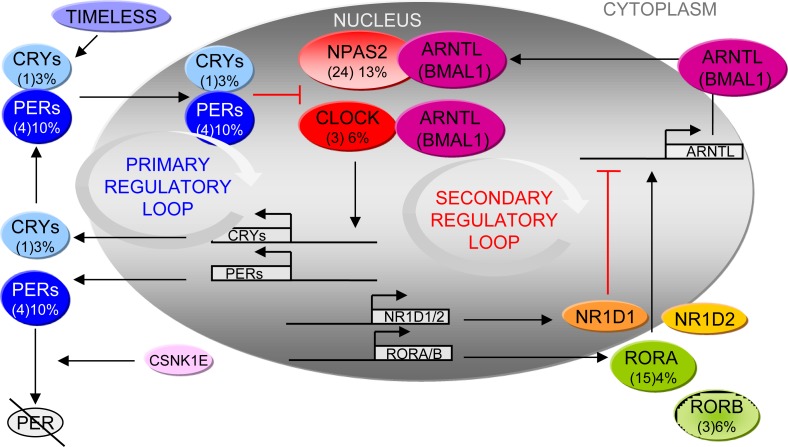
Hypothetical clock mechanism in mammals Primary regulatory loop: the transcription of *PERs* and *CRYs* genes is activated by heterodimers composed of ARNTL (BMAL1) and either CLOCK or NPAS2. PERs and CRYs, repress their own transcription by acting directly on the ARNTL (BMAL1)-CLOCK/NPAS2 complex. PER proteins interact with CRY proteins to form a protein complex. Both CRY and PER are phosphorylated by kinases, as CSNK1E, which triggers their degradation unless they are within the protein complex. Secondary regulatory loop: ARNTL (BMAL1)-CLOCK/NPAS2 heterodimers activate the transcription of retinoic acid-related orphan nuclear receptors, *NR1D1 NR1D2*, *RORA* and *RORB*. RORA activates transcription of *ARNTL*, whereas NR1D1 and NR1D2 represses it. TIMELESS interacts directly with CRY1. For each gene is indicated the percentage of SNPs statistically significantly associated with cancer risk out of the meta-analyses performed. Brackets: number of SNPs statistically significantly associated with cancer risk. See also Supplementary Table S2.

We also investigated heterogeneity. In particular, between-study heterogeneity was formally assessed by using the Cochran Q-test and the I-squared statistic (the latter indicating the proportion of the variability in effect estimates linked to true between-study heterogeneity as opposed to within-study sampling error).

### Assessment of cumulative evidence

With the aim to assess the credibility of statistically significant associations based on the results of data meta-analysis, we used the Venice criteria [40, 42]. In brief, we defined credibility levels based on the strength (classified as A=strong, B=moderate or C=weak) of three parameters: amount of the evidence, replication of the association and protection from bias. We graded the amount of evidence, which approximately depends on the study sample size, based on the sum of cases and controls: accordingly, we assigned grade A, B or C to meta-analyses with total sample size >1000, 100–1000 and <100, respectively. We graded the replication of the association based on the amount of between-study heterogeneity: in particular, we assigned grade A, B or C to meta-analyses with I-squared <25%, 25–50% and >50%, respectively. We graded protection from bias as A if no bias was observed, B if bias was potentially present or C if bias was evident. While assessing protection from bias we also considered the magnitude of the association: we assigned a score of C to an association characterized by a summary OR <1.15 (or a summary OR>.87 if the effect of the polymorphism was protective).

Besides the Venice criteria, we also considered the noteworthiness of significant findings by analyzing the so called false positive report probability (FPRP) [48]. The FPRP is defined as the probability of no true association in the presence of a statistically significant result. According to a Bayesian approach, the FPRP depends on the observed P-value of the association test, the statistical power of the test, and the prior probability that the association is true. FPRP values were calculated for the 0.01 prior probability level. As recommended, a FPRP cut-off value of 0.2 was used to classify a nominally statistically significant association as “noteworthy” [48].

Overall, we defined the credibility level of the cumulative evidence as high (Venice criteria A grades only coupled with “noteworthy” finding at FPRP analysis), low (one or more C grades combined with lack of noteworthiness) or intermediate (for all other combinations).

## SUPPLEMENTARY MATERIALS TABLES






