# Supplementary material for: Genetic variation of clock genes and cancer risk: a field synopsis and meta-analysis
Source: Oncotarget. 2017 Feb 3;8(14):23978–95. doi: 10.18632/oncotarget.15074 (PMC5410358; doi:10.18632/oncotarget.15074)
Supplement: Supplementary file 1 [file oncotarget-08-23978-s001.doc]

| **Supplementary Table S1** Characteristics of the work conditions in the six eligible studies included in this meta-analysis | | | | | | | | | |
| --- | --- | --- | --- | --- | --- | --- | --- | --- | --- |
| **First author** | **Year** | **Cancer type** | **# Cases** | **# Controls** | **Night work duration** | **Minimum work duration** | **Night shift definition** | **Shift intensity and duration** | **Parameters** |
| **Grundy** | 2013 | BREAST CANCER | 953 | 974 | <2 years,  ≥2 years | 6 months | night/evening shifts (≥ 50% time) |  | Never  0-14 years  15-29 years  ≥ 30years |
| **Monsees** | 2012 | BREAST CANCER | 609 | 1216 | <2 years,  ≥2 years |  |  | at least 3 nights/month in addition to days and evenings the same month |  |
| **Rabstein** | 2014 | BREAST CANCER | 1022 | 1014 | ever (at least 1 year), never |  | 12 am to 5am | full time | Never employed in shiftwork  Ever shift or less than 20 years night work  Night work for 20 years or longer |
| **Rana** | 2014 | CLL - Chronic Lymphotic Leukemia | 37 | 37 |  |  |  |  | Shiftworker  Non shiftworker |
| **Truong** | 2014 | BREAST CANCER | 1126 | 1174 | <2 years,  ≥2 years | 6 months | 11 am to 5 am | at least 1h | Night work never  Night work ever |
| **Zienolddiny** | 2013 | BREAST CANCER | 563 | 619 | Maximum number of consecutive night shifts (ever)  Maximum number of consecutive night shifts during a minimum of 5 years |  | 12 am to 6 am (rotating or permanent) | No restrictions | Only day work or one or two consecutive night shifts;  Three consecutive night shifts;  Four or more consecutive night shifts |
