# Supplementary material for: Genetic variation of clock genes and cancer risk: a field synopsis and meta-analysis
Source: Oncotarget. 2017 Feb 3;8(14):23978–95. doi: 10.18632/oncotarget.15074 (PMC5410358; doi:10.18632/oncotarget.15074)
Supplement: Supplementary file 2 [file oncotarget-08-23978-s002.doc]

| **GENE** | **MAP** | **N. POLYMOPHISMS** | **N. POLYMORPHISMS AVAILABLE FOR ANALYSIS (2 or more datasets)** | **N. ANALYSIS (INCLUDING SUBGROUPS)** | **N. SIGNIFICANT ANALYSIS** | **% SIGNIFICANT ANALYSIS** |
| --- | --- | --- | --- | --- | --- | --- |
| ARNTL | 11p15 | 55 | 30 | 76 | 0 | 0.0 |
| CLOCK | 4q12 | 41 | 17 | 47 | 3 | 6.4 |
| CRY1 | 12q23-q24.1 | 16 | 6 | 20 | 0 | 0.0 |
| CRY2 | 11p11.2 | 21 | 13 | 40 | 1 | 2.5 |
| CSNK1E | 22q13.1 | 12 | 8 | 25 | 0 | 0.0 |
| NPAS2 | 2q11.2 | 121 | 64 | 183 | 24 | 13.1 |
| NR1D1 | 17q11.2 | 9 | 7 | 14 | 0 | 0.0 |
| NR1D2 | 3p24 | 0 | 0 | 0 | 0 | 0.0 |
| PER1 | 17p13.1 | 14 | 10 | 24 | 1 | 4.2 |
| PER2 | 2q37.3 | 20 | 10 | 33 | 1 | 3.0 |
| PER3 | 1p36.23 | 41 | 24 | 63 | 2 | 3.2 |
| RORA | 15q21-q22 | 289 | 151 | 429 | 15 | 3.5 |
| RORB | 9q22 | 35 | 19 | 53 | 3 | 5.7 |
| TIMELESS | 12q13.3 | 13 | 7 | 18 | 0 | 0.0 |
| **TOTAL** |  | **687** | **366** | **1025** | **50** |  |
