# Supplementary material for: Genetic variation of clock genes and cancer risk: a field synopsis and meta-analysis
Source: Oncotarget. 2017 Feb 3;8(14):23978–95. doi: 10.18632/oncotarget.15074 (PMC5410358; doi:10.18632/oncotarget.15074)
Supplement: Supplementary file 3 [file oncotarget-08-23978-s003.docx]

| **PRIMARY META-ANALISYS** | | | | | | | | | | | | |
| --- | --- | --- | --- | --- | --- | --- | --- | --- | --- | --- | --- | --- |
| **SNP ID** | **GENE** | **CANCER** | **STUDIES** | **OR** | **CI Lower** | **CI Upper** | **I ^2^ %** | **P value** | **Cases** | **Controls** | **Common (Allele2)** | **Minor (Allele1)** |
| 4/5 repeats | PER3 | MIXED | 5 | 1,08 | 0,98 | 1,19 | 0 | 0,11 | 2771 | 3008 | 4 repeats | 5 repeats |
| rs1005473 | CSNK1E | MIXED | 3 | 0,96 | 0,72 | 1,28 | 0 | 0,77 | 947 | 2084 | A | C |
| rs1012477 | PER3 | MIXED | 6 | 1 | 0,9 | 1,11 | 37 | 0,99 | 4710 | 5875 | C | G |
| rs10127838 | PER3 | MIXED | 2 | 1,02 | 0,89 | 1,16 | 48 | 0,81 | 6553 | 9660 | C | A |
| **rs10162630** | **RORA** | **MIXED** | **3** | **1,06** | **1** | **1,12** | **0** | **0,04** | **6122** | **6250** | A | G |
| **rs10165970** | **NPAS2** | **MIXED** | **4** | **1,1** | **1,03** | **1,17** | **0** | **0,002** | **8288** | **12050** | G | A |
| rs1044245 | PER3 | MIXED | 3 | 1,02 | 0,94 | 1,11 | 7 | 0,6 | 5605 | 6292 | G | A |
| rs1044432 | ARNTL | MIXED | 2 | 0,96 | 0,76 | 1,21 | 75 | 0,7 | 4460 | 5150 | A | T |
| rs10462018 | PER3 | MIXED | 4 | 0,97 | 0,86 | 1,1 | 68 | 0,68 | 7817 | 12048 | G | A |
| rs10462020 | PER3 | MIXED | 6 | 1,02 | 0,97 | 1,08 | 0 | 0,36 | 9203 | 13388 | T | G |
| rs10462021 | PER3 | MIXED | 3 | 1,03 | 0,97 | 1,09 | 18 | 0,39 | 7679 | 10834 | A | G |
| rs10462023 | PER2 | MIXED | 6 | 1,01 | 0,95 | 1,07 | 39 | 0,84 | 9448 | 14278 | G | A |
| rs10462028 | CLOCK | BREAST | 2 | 1,3 | 0,66 | 2,57 | 98 | 0,45 | 2148 | 2188 | G | A |
| rs10519070 | RORA | MIXED | 3 | 0,98 | 0,91 | 1,06 | 0 | 0,67 | 6122 | 6250 | A | G |
| **rs10519097** | **RORA** | **MIXED** | **3** | **0,91** | **0,84** | **0,99** | **9** | **0,02** | **6122** | **6250** | G | A |
| rs10519099 | RORA | MIXED | 3 | 0,97 | 0,91 | 1,03 | 6 | 0,3 | 6122 | 6250 | A | G |
| rs10519111 | RORA | MIXED | 3 | 0,98 | 0,9 | 1,05 | 5 | 0,54 | 6122 | 6250 | A | G |
| **rs1053096** | **NPAS2** | **MIXED** | **3** | **0,93** | **0,89** | **0,98** | **0** | **0,007** | **6691** | **10874** | C | T |
| rs10766074 | ARNTL | BREAST | 2 | 1 | 0,88 | 1,12 | 0 | 0,96 | 1735 | 2390 | A | G |
| rs10778528 | CRY1 | MIXED | 3 | 0,99 | 0,95 | 1,04 | 0 | 0,79 | 7861 | 10926 | T | C |
| rs10778534 | CRY1 | MIXED | 2 | 1,04 | 0,95 | 1,13 | 0 | 0,43 | 1917 | 2482 | A | G |
| rs10781235 | RORB | MIXED | 3 | 0,96 | 0,9 | 1,02 | 0 | 0,18 | 6122 | 6250 | G | A |
| rs10832027 | ARNTL | MIXED | 4 | 0,99 | 0,96 | 1,02 | 0 | 0,45 | 10923 | 13598 | A | G |
| rs10838524 | CRY2 | BREAST | 2 | 0,98 | 0,83 | 1,17 | 76 | 0,83 | 2148 | 2188 | G | A |
| rs10838527 | CRY2 | MIXED | 4 | 1,03 | 0,98 | 1,08 | 0 | 0,29 | 21003 | 37108 | A | G |
| rs10864316 | PER3 | MIXED | 2 | 1,02 | 0,93 | 1,13 | 61 | 0,65 | 6553 | 9660 | A | G |
| rs10869410 | RORB | MIXED | 3 | 1 | 0,94 | 1,06 | 0 | 0,94 | 6122 | 6250 | A | G |
| rs10869417 | RORB | MIXED | 3 | 0,95 | 0,89 | 1,01 | 0 | 0,13 | 6122 | 6250 | G | A |
| rs10876889 | TIMELESS | MIXED | 3 | 1,03 | 0,97 | 1,09 | 0 | 0,35 | 5134 | 6290 | G | A |
| rs11022742 | ARNTL | MIXED | 3 | 0,97 | 0,91 | 1,04 | 0 | 0,39 | 5605 | 6292 | A | G |
| rs11022775 | ARNTL | MIXED | 2 | 0,98 | 0,83 | 1,16 | 68 | 0,82 | 6553 | 9660 | C | T |
| rs11022778 | ARNTL | MIXED | 5 | 1,02 | 0,95 | 1,09 | 46 | 0,61 | 8426 | 13264 | T | G |
| rs11022780 | ARNTL | MIXED | 2 | 0,99 | 0,94 | 1,04 | 0 | 0,62 | 6553 | 9660 | T | C |
| rs11038689 | CRY2 | MIXED | 3 | 0,94 | 0,83 | 1,07 | 0 | 0,35 | 1459 | 1625 | A | G |
| rs11038695 | CRY2 | MIXED | 3 | 1,05 | 1 | 1,1 | 0 | 0,06 | 8738 | 7830 | G | A |
| rs11038699 | CRY2 | MIXED | 2 | 1,02 | 0,89 | 1,17 | 0 | 0,77 | 747 | 2430 | A | G |
| rs11071587 | RORA | MIXED | 3 | 0,95 | 0,9 | 1 | 0 | 0,06 | 6122 | 6250 | G | A |
| rs11071588 | RORA | MIXED | 3 | 1,01 | 0,96 | 1,08 | 7 | 0,64 | 6122 | 6250 | C | A |
| rs11113179 | CRY1 | MIXED | 5 | 1,04 | 0,95 | 1,13 | 9 | 0,37 | 7684 | 8440 | C | T |
| rs11121023 | PER3 | PROSTATE | 2 | 0,99 | 0,87 | 1,12 | 0 | 0,82 | 1446 | 2480 | G | A |
| rs11123857 | NPAS2 | MIXED | 6 | 0,99 | 0,9 | 1,07 | 68 | 0,74 | 9448 | 14278 | A | G |
| rs11133373 | CLOCK | MIXED | 3 | 0,99 | 0,88 | 1,11 | 44 | 0,81 | 2480 | 3101 | C | G |
| rs11133375 | CLOCK | MIXED | 3 | 1,02 | 0,96 | 1,08 | 0 | 0,59 | 5605 | 6292 | G | A |
| rs11133391 | CLOCK | MIXED | 3 | 0,99 | 0,87 | 1,12 | 48 | 0,83 | 2189 | 2281 | A | G |
| rs11171846 | TIMELESS | MIXED | 3 | 0,97 | 0,89 | 1,06 | 0 | 0,48 | 6122 | 6250 | G | A |
| rs1159814 | RORA | MIXED | 3 | 1,03 | 0,97 | 1,09 | 0 | 0,3 | 6122 | 6250 | A | G |
| rs11600996 | ARNTL | MIXED | 2 | 0,95 | 0,85 | 1,06 | 0 | 0,35 | 1264 | 2388 | C | T |
| rs11605924 | CRY2 | MIXED | 4 | 0,96 | 0,89 | 1,04 | 0 | 0,33 | 3349 | 3414 | A | C |
| rs1160694 | RORA | MIXED | 3 | 1,02 | 0,94 | 1,11 | 39 | 0,59 | 6122 | 6250 | G | A |
| rs11629597 | RORA | MIXED | 3 | 0,99 | 0,93 | 1,06 | 0 | 0,82 | 6122 | 6250 | A | G |
| rs11630018 | RORA | MIXED | 3 | 1,04 | 0,96 | 1,13 | 0 | 0,35 | 6122 | 6250 | G | A |
| rs11630062 | RORA | MIXED | 3 | 1,01 | 0,9 | 1,13 | 25 | 0,93 | 6122 | 6250 | A | G |
| rs11630227 | RORA | MIXED | 3 | 0,99 | 0,93 | 1,04 | 0 | 0,63 | 6122 | 6250 | A | G |
| rs11631432 | RORA | MIXED | 3 | 1,01 | 0,94 | 1,08 | 23 | 0,82 | 6122 | 6250 | G | A |
| rs11632858 | RORA | MIXED | 2 | 0,84 | 0,55 | 1,27 | 81 | 0,41 | 4977 | 5108 | C | A |
| rs11634318 | RORA | MIXED | 3 | 0,96 | 0,87 | 1,07 | 0 | 0,49 | 6122 | 6250 | C | A |
| rs11635314 | RORA | MIXED | 3 | 0,97 | 0,89 | 1,06 | 18 | 0,55 | 6122 | 6250 | G | A |
| **rs11674199** | **NPAS2** | **MIXED** | **3** | 1,08 | **1,03** | **1,14** | **0** | **0,002** | **6691** | **10874** | G | A |
| rs11695472 | PER2 | MIXED | 4 | 1,05 | 0,99 | 1,11 | 0 | 0,09 | 6867 | 6961 | A | C |
| rs11894535 | PER2 | BREAST | 2 | 1 | 0,9 | 1,13 | 0 | 0,94 | 1562 | 2190 | C | T |
| rs11894671 | NPAS2 | MIXED | 4 | 1,03 | 0,98 | 1,09 | 2 | 0,24 | 7300 | 12090 | G | T |
| rs11932595 | CLOCK | MIXED | 6 | 1 | 0,93 | 1,08 | 42 | 0,98 | 7346 | 8749 | A | G |
| **rs11943456** | **CLOCK** | **MIXED** | **3** | **1,11** | **1** | **1,24** | **0** | **0,05** | **5605** | **6292** | A | G |
| rs12101478 | RORA | MIXED | 3 | 1 | 0,92 | 1,09 | 21 | 0,93 | 6122 | 6250 | A | G |
| rs12281674 | CRY2 | MIXED | 3 | 0,86 | 0,72 | 1,03 | 0 | 0,1 | 6304 | 6342 | A | G |
| rs12315175 | CRY1 | MIXED | 6 | 1 | 0,94 | 1,07 | 12 | 0,9 | 7673 | 9400 | A | G |
| rs12324380 | RORA | MIXED | 2 | 0,97 | 0,84 | 1,13 | 0 | 0,71 | 4977 | 5108 | A | G |
| rs12324440 | RORA | MIXED | 3 | 1 | 0,94 | 1,06 | 0 | 0,99 | 6122 | 6250 | G | A |
| rs12421530 | ARNTL | MIXED | 3 | 0,99 | 0,9 | 1,08 | 0 | 0,79 | 1873 | 3604 | C | G |
| rs12438866 | RORA | MIXED | 3 | 0,96 | 0,83 | 1,11 | 77 | 0,58 | 6122 | 6250 | A | G |
| rs12440095 | RORA | MIXED | 3 | 0,97 | 0,9 | 1,05 | 0 | 0,43 | 6122 | 6250 | G | A |
| rs12443239 | RORA | MIXED | 3 | 0,98 | 0,84 | 1,15 | 81 | 0,84 | 6122 | 6250 | A | G |
| rs12472321 | NPAS2 | MIXED | 3 | 0,95 | 0,86 | 1,05 | 0 | 0,3 | 1873 | 3604 | A | G |
| rs12591650 | RORA | MIXED | 3 | 0,99 | 0,92 | 1,07 | 0 | 0,85 | 6122 | 6250 | A | G |
| rs12593790 | RORA | MIXED | 2 | 0,99 | 0,72 | 1,35 | 70 | 0,94 | 4977 | 5108 | A | G |
| rs12594972 | RORA | MIXED | 3 | 0,97 | 0,89 | 1,06 | 0 | 0,48 | 6122 | 6250 | A | G |
| **rs12622050** | **NPAS2** | **MIXED** | **2** | **0,94** | **0,89** | **1** | **0** | **0,04** | **6553** | **9660** | G | A |
| rs12712083 | NPAS2 | MIXED | 5 | 0,94 | 0,86 | 1,03 | 53 | 0,18 | 6869 | 8680 | A | G |
| **rs12712085** | **NPAS2** | **BREAST** | **2** | **0,89** | **0,81** | **0,97** | **0** | **0,01** | **1735** | **2390** | G | A |
| rs12821586 | CRY1 | MIXED | 3 | 0,99 | 0,79 | 1,24 | 71 | 0,94 | 6122 | 6250 | G | A |
| rs12909890 | RORA | MIXED | 3 | 1 | 0,95 | 1,06 | 0 | 0,98 | 6122 | 6250 | A | G |
| rs12913421 | RORA | MIXED | 3 | 0,92 | 0,78 | 1,09 | 68 | 0,34 | 5175 | 5642 | G | A |
| rs12941497 | NR1D1 | MIXED | 3 | 1 | 0,94 | 1,06 | 0 | 0,97 | 6122 | 6250 | G | A |
| rs13025524 | NPAS2 | MIXED | 4 | 0,97 | 0,91 | 1,05 | 31 | 0,46 | 6731 | 7466 | G | A |
| rs13026599 | NPAS2 | BREAST | 2 | 1,05 | 0,94 | 1,18 | 0 | 0,4 | 1735 | 2390 | A | G |
| rs1323369 | RORB | MIXED | 3 | 0,96 | 0,88 | 1,04 | 40 | 0,34 | 6122 | 6250 | A | G |
| rs1327836 | RORB | MIXED | 3 | 0,96 | 0,89 | 1,03 | 0 | 0,28 | 6122 | 6250 | A | C |
| rs1327837 | RORB | MIXED | 3 | 0,97 | 0,91 | 1,02 | 0 | 0,26 | 6122 | 6250 | G | A |
| rs13329238 | RORA | MIXED | 3 | 1,05 | 0,97 | 1,13 | 30 | 0,21 | 6122 | 6250 | A | C |
| rs13329643 | RORA | MIXED | 3 | 0,96 | 0,89 | 1,03 | 0 | 0,26 | 6122 | 6250 | G | A |
| rs13394520 | NPAS2 | MIXED | 2 | 1,01 | 0,89 | 1,14 | 0 | 0,93 | 747 | 2430 | A | G |
| rs1351545 | RORA | MIXED | 3 | 0,99 | 0,92 | 1,06 | 15 | 0,77 | 6122 | 6250 | G | A |
| rs1351546 | RORA | MIXED | 3 | 0,99 | 0,94 | 1,05 | 0 | 0,71 | 6122 | 6250 | G | A |
| rs135750 | CSNK1E | MIXED | 2 | 1,04 | 0,99 | 1,08 | 0 | 0,1 | 4887 | 3896 | C | G |
| rs135757 | CSNK1E | MIXED | 5 | 1,01 | 0,95 | 1,06 | 0 | 0,86 | 7395 | 8530 | G | A |
| rs1369481 | NPAS2 | MIXED | 7 | 0,95 | 0,88 | 1,02 | 57 | 0,19 | 10356 | 15158 | G | A |
| rs1401417 | CRY2 | MIXED | 10 | 0,96 | 0,89 | 1,04 | 39 | 0,35 | 6324 | 8510 | C | G |
| rs1403737 | RORA | MIXED | 3 | 1,02 | 0,96 | 1,09 | 1 | 0,46 | 6122 | 6250 | G | A |
| rs1407845 | RORB | MIXED | 3 | 0,95 | 0,87 | 1,04 | 44 | 0,25 | 6122 | 6250 | A | G |
| rs1410226 | RORB | MIXED | 3 | 1,05 | 0,96 | 1,15 | 0 | 0,3 | 6122 | 6250 | A | C |
| rs1410227 | RORB | MIXED | 3 | 0,98 | 0,91 | 1,06 | 0 | 0,6 | 6122 | 6250 | A | G |
| rs1425287 | RORA | MIXED | 3 | 1,03 | 0,96 | 1,1 | 0 | 0,4 | 6122 | 6250 | G | A |
| rs1437543 | RORA | MIXED | 3 | 1,02 | 0,96 | 1,09 | 0 | 0,52 | 6122 | 6250 | G | A |
| rs1437547 | RORA | MIXED | 3 | 1,01 | 0,87 | 1,17 | 57 | 0,92 | 6122 | 6250 | A | G |
| rs1437550 | RORA | MIXED | 3 | 1 | 0,93 | 1,07 | 0 | 0,96 | 6122 | 6250 | A | C |
| rs1442849 | PER1 | MIXED | 3 | 1,05 | 0,99 | 1,12 | 0 | 0,11 | 5605 | 6292 | G | A |
| rs1465812 | RORA | MIXED | 3 | 0,97 | 0,92 | 1,02 | 0 | 0,25 | 6122 | 6250 | G | A |
| rs1482049 | RORA | MIXED | 3 | 0,99 | 0,93 | 1,06 | 0 | 0,86 | 6122 | 6250 | G | A |
| rs1482058 | RORA | MIXED | 3 | 1,03 | 0,97 | 1,09 | 0 | 0,32 | 6122 | 6250 | G | A |
| rs1522113 | CLOCK | MIXED | 2 | 0,9 | 0,81 | 1 | 0 | 0,06 | 6553 | 9660 | G | A |
| rs1534891 | CSNK1E | MIXED | 7 | 0,98 | 0,93 | 1,04 | 0 | 0,6 | 10310 | 14561 | C | T |
| **rs1542178** | **NPAS2** | **MIXED** | **4** | **0,94** | **0,89** | **1** | **0** | **0,04** | **6260** | **7464** | G | A |
| rs1542179 | NPAS2 | MIXED | 3 | 0,98 | 0,89 | 1,09 | 72 | 0,76 | 7679 | 10834 | T | C |
| rs1561002 | NPAS2 | MIXED | 3 | 0,96 | 0,82 | 1,12 | 81 | 0,59 | 6691 | 10874 | T | C |
| rs1562313 | NPAS2 | MIXED | 5 | 0,97 | 0,92 | 1,02 | 0 | 0,19 | 8608 | 13356 | C | T |
| rs1562438 | ARNTL | MIXED | 4 | 0,98 | 0,95 | 1,02 | 0 | 0,36 | 5634 | 6326 | C | T |
| rs1589702 | RORA | MIXED | 3 | 1,01 | 0,92 | 1,11 | 54 | 0,84 | 6122 | 6250 | G | A |
| rs1589703 | RORA | MIXED | 3 | 0,98 | 0,91 | 1,05 | 0 | 0,53 | 6122 | 6250 | A | G |
| **rs1632660** | **RORA** | **MIXED** | **3** | **0,94** | **0,88** | **1** | **0** | **0,05** | **6122** | **6250** | G | A |
| rs1680446 | RORA | MIXED | 2 | 0,99 | 0,84 | 1,15 | 0 | 0,85 | 4977 | 5108 | G | A |
| rs16912751 | ARNTL | MIXED | 4 | 0,99 | 0,94 | 1,05 | 0 | 0,81 | 11440 | 13556 | T | C |
| rs16942820 | RORA | MIXED | 2 | 1,13 | 0,88 | 1,45 | 59 | 0,35 | 4977 | 5108 | C | A |
| rs16943087 | RORA | MIXED | 3 | 1,05 | 0,93 | 1,17 | 45 | 0,45 | 6122 | 6250 | A | G |
| rs16943318 | RORA | MIXED | 3 | 0,98 | 0,92 | 1,04 | 0 | 0,5 | 6122 | 6250 | G | A |
| rs16943453 | RORA | MIXED | 3 | 0,98 | 0,91 | 1,06 | 11 | 0,62 | 6122 | 6250 | A | C |
| rs16943579 | RORA | MIXED | 3 | 1 | 0,83 | 1,2 | 60 | 1 | 6122 | 6250 | G | A |
| rs17020663 | NPAS2 | BREAST | 2 | 1 | 0,87 | 1,14 | 0 | 0,97 | 1735 | 2390 | G | C |
| rs17024869 | NPAS2 | MIXED | 3 | 0,89 | 0,75 | 1,05 | 64 | 0,17 | 6122 | 6250 | A | G |
| rs17024926 | NPAS2 | MIXED | 7 | 1,04 | 0,99 | 1,1 | 32 | 0,14 | 10310 | 14561 | T | C |
| rs17025005 | NPAS2 | BREAST | 2 | 0,9 | 0,81 | 1,01 | 0 | 0,08 | 1735 | 2390 | G | A |
| rs17191442 | RORA | MIXED | 3 | 1,03 | 0,95 | 1,12 | 0 | 0,48 | 6122 | 6250 | A | G |
| rs17191554 | RORA | MIXED | 3 | 0,99 | 0,77 | 1,27 | 77 | 0,94 | 6122 | 6250 | C | A |
| rs17204440 | RORA | MIXED | 2 | 1,05 | 0,88 | 1,24 | 85 | 0,61 | 4977 | 5108 | A | C |
| rs17204573 | RORA | MIXED | 3 | 1,01 | 0,92 | 1,1 | 0 | 0,89 | 6122 | 6250 | G | A |
| rs17204698 | RORA | MIXED | 3 | 1,05 | 0,98 | 1,12 | 0 | 0,15 | 6122 | 6250 | A | C |
| rs17204770 | RORA | MIXED | 3 | 0,98 | 0,92 | 1,03 | 0 | 0,41 | 6122 | 6250 | G | A |
| rs17204938 | RORA | MIXED | 2 | 0,93 | 0,79 | 1,09 | 1 | 0,35 | 4977 | 5108 | A | C |
| rs17237367 | RORA | MIXED | 3 | 0,99 | 0,88 | 1,12 | 58 | 0,92 | 6122 | 6250 | G | A |
| rs17237486 | RORA | MIXED | 3 | 0,98 | 0,92 | 1,05 | 0 | 0,59 | 6122 | 6250 | A | G |
| rs17237521 | RORA | MIXED | 3 | 0,97 | 0,89 | 1,07 | 21 | 0,57 | 6122 | 6250 | A | G |
| rs17237563 | RORA | MIXED | 3 | 1,03 | 0,92 | 1,15 | 36 | 0,6 | 6122 | 6250 | G | A |
| rs17270188 | RORA | MIXED | 3 | 1,06 | 0,95 | 1,17 | 59 | 0,3 | 6122 | 6250 | A | G |
| rs17270216 | RORA | MIXED | 3 | 0,95 | 0,82 | 1,09 | 73 | 0,44 | 6122 | 6250 | A | G |
| rs17303097 | RORA | MIXED | 3 | 0,95 | 0,89 | 1,02 | 0 | 0,17 | 6122 | 6250 | A | C |
| rs17452383 | ARNTL | BREAST | 2 | 0,99 | 0,86 | 1,13 | 5 | 0,87 | 1735 | 2390 | A | G |
| rs17612183 | RORB | MIXED | 3 | 0,97 | 0,89 | 1,07 | 15 | 0,56 | 6122 | 6250 | G | A |
| rs17654772 | NPAS2 | MIXED | 3 | 1,04 | 0,92 | 1,18 | 60 | 0,54 | 5605 | 6292 | G | A |
| rs17691363 | RORB | MIXED | 2 | 0,99 | 0,87 | 1,14 | 0 | 0,92 | 2271 | 2316 | A | G |
| rs17717414 | NPAS2 | MIXED | 2 | 1,07 | 0,89 | 1,28 | 10 | 0,47 | 747 | 2430 | A | G |
| rs17776421 | CLOCK | MIXED | 4 | 1,01 | 0,96 | 1,07 | 0 | 0,64 | 6913 | 7558 | G | A |
| rs1801260 | CLOCK | MIXED | 4 | 1,15 | 0,87 | 1,52 | 91 | 0,33 | 2578 | 3349 | T | C |
| rs1811399 | NPAS2 | MIXED | 3 | 0,97 | 0,85 | 1,12 | 76 | 0,71 | 7836 | 12016 | A | C |
| rs1902618 | RORA | MIXED | 3 | 0,97 | 0,91 | 1,04 | 0 | 0,43 | 6122 | 6250 | A | G |
| rs1916645 | RORA | MIXED | 3 | 0,99 | 0,84 | 1,17 | 66 | 0,93 | 6122 | 6250 | G | A |
| rs1997644 | CSNK1E | MIXED | 4 | 0,98 | 0,92 | 1,05 | 27 | 0,62 | 7430 | 7516 | A | G |
| rs2011857 | RORA | MIXED | 3 | 1 | 0,91 | 1,09 | 46 | 0,99 | 6122 | 6250 | A | G |
| rs2028122 | RORA | MIXED | 3 | 0,96 | 0,89 | 1,04 | 32 | 0,31 | 6122 | 6250 | G | A |
| rs2030619 | RORA | MIXED | 3 | 0,95 | 0,87 | 1,03 | 15 | 0,2 | 6122 | 6250 | A | C |
| rs2035691 | CLOCK | BREAST | 1 | 1,11 | 0,96 | 1,27 | 0 | 0,15 | 953 | 974 | G | A |
| rs2062094 | RORA | MIXED | 3 | 1,02 | 0,96 | 1,08 | 0 | 0,51 | 6122 | 6250 | A | G |
| rs2071427 | NR1D1 | MIXED | 3 | 0,99 | 0,95 | 1,05 | 0 | 0,83 | 7679 | 10834 | G | A |
| rs2071570 | NR1D1 | MIXED | 3 | 1,02 | 0,96 | 1,07 | 0 | 0,56 | 7679 | 10834 | C | A |
| rs2075984 | CSNK1E | MIXED | 4 | 1,01 | 0,97 | 1,06 | 0 | 0,51 | 7817 | 12048 | A | C |
| rs2117714 | NPAS2 | MIXED | 2 | 0,99 | 0,93 | 1,06 | 26 | 0,87 | 6553 | 9660 | T | C |
| rs2118326 | RORA | MIXED | 3 | 1 | 0,94 | 1,07 | 0 | 0,97 | 6122 | 6250 | G | A |
| rs2253820 | PER1 | MIXED | 5 | 0,97 | 0,86 | 1,09 | 76 | 0,58 | 9596 | 13316 | G | A |
| rs2269457 | NR1D1 | MIXED | 3 | 1 | 0,95 | 1,05 | 0 | 0,93 | 7679 | 10834 | T | C |
| rs2278637 | PER1 | MIXED | 3 | 0,95 | 0,89 | 1,01 | 0 | 0,08 | 5605 | 6292 | A | C |
| rs2278749 | ARNTL | MIXED | 4 | 0,95 | 0,86 | 1,05 | 44 | 0,35 | 6867 | 6961 | C | T |
| rs228642 | PER3 | MIXED | 3 | 0,97 | 0,92 | 1,01 | 0 | 0,15 | 7679 | 10834 | T | C |
| rs228644 | PER3 | MIXED | 4 | 1,01 | 0,96 | 1,06 | 7 | 0,76 | 4586 | 5226 | G | A |
| rs228648 | PER3 | MIXED | 3 | 0,99 | 0,89 | 1,11 | 57 | 0,91 | 5605 | 6292 | A | G |
| rs228651 | PER3 | MIXED | 3 | 0,95 | 0,9 | 1,01 | 0 | 0,12 | 5605 | 6292 | G | A |
| rs228654 | PER3 | MIXED | 2 | 0,99 | 0,91 | 1,07 | 0 | 0,84 | 6553 | 9660 | C | T |
| rs228682 | PER3 | MIXED | 4 | 1 | 0,98 | 1,03 | 0 | 0,79 | 11440 | 13556 | T | C |
| rs228697 | PER3 | PROSTATE | 2 | 1,11 | 0,94 | 1,32 | 0 | 0,77 | 1446 | 2480 | C | G |
| rs228729 | PER3 | MIXED | 6 | 1,01 | 0,94 | 1,09 | 45 | 0,71 | 8504 | 13338 | C | T |
| rs2289591 | PER1 | MIXED | 3 | 0,98 | 0,8 | 1,22 | 71 | 0,88 | 2068 | 3108 | C | A |
| rs2290035 | ARNTL | MIXED | 2 | 1 | 0,92 | 1,1 | 0 | 0,93 | 1871 | 1885 | A | T |
| rs2290037 | ARNTL | MIXED | 3 | 0,97 | 0,88 | 1,07 | 0 | 0,59 | 7679 | 10834 | T | C |
| rs2291738 | TIMELESS | MIXED | 4 | 1,01 | 0,95 | 1,06 | 0 | 0,82 | 6563 | 6729 | A | G |
| rs2292910 | CRY2 | MIXED | 4 | 1,03 | 0,96 | 1,1 | 0 | 0,48 | 3181 | 4870 | C | A |
| rs2292912 | CRY2 | MIXED | 5 | 0,92 | 0,84 | 1 | 25 | 0,06 | 3952 | 4074 | C | G |
| rs2304673 | PER2 | MIXED | 3 | 1 | 0,94 | 1,07 | 0 | 1 | 7679 | 10834 | A | C |
| rs2304674 | PER2 | MIXED | 4 | 1,01 | 0,96 | 1,07 | 0 | 0,64 | 8288 | 12050 | T | C |
| rs2304911 | PER1 | MIXED | 2 | 0,93 | 0,8 | 1,07 | 6 | 0,28 | 4977 | 5108 | A | G |
| rs2305159 | NPAS2 | MIXED | 4 | 0,98 | 0,89 | 1,07 | 60 | 0,6 | 7300 | 12090 | A | C |
| **rs2305160** | **NPAS2** | **MIXED** | **14** | 0,96 | **0,91** | **1,01** | **36** | **0,08** | **13686** | **18890** | C | T |
| rs2306074 | ARNTL2 | MIXED | 3 | 1 | 0,94 | 1,07 | 3 | 0,98 | 5559 | 5695 | T | C |
| rs2306502 | RORA | MIXED | 3 | 1,01 | 0,92 | 1,11 | 0 | 0,83 | 6122 | 6250 | A | C |
| rs2314339 | NR1D1 | MIXED | 3 | 0,97 | 0,89 | 1,05 | 0 | 0,41 | 6122 | 6250 | C | T |
| rs2412648 | CLOCK | MIXED | 2 | 1,01 | 0,96 | 1,06 | 0 | 0,74 | 6553 | 9660 | T | G |
| rs2414686 | RORA | MIXED | 3 | 0,96 | 0,91 | 1,02 | 0 | 0,16 | 6122 | 6250 | G | A |
| rs2414687 | RORA | MIXED | 3 | 0,99 | 0,93 | 1,06 | 0 | 0,82 | 6122 | 6250 | C | A |
| rs2585405 | PER1 | MIXED | 3 | 1,04 | 0,95 | 1,14 | 0 | 0,37 | 2347 | 2475 | G | C |
| rs2585408 | PER1 | MIXED | 2 | 0,98 | 0,91 | 1,05 | 51 | 0,53 | 6553 | 9660 | C | T |
| rs2640909 | PER3 | MIXED | 3 | 1,02 | 0,86 | 1,21 | 65 | 0,81 | 2055 | 3696 | T | C |
| rs2689352 | RORA | MIXED | 3 | 0,98 | 0,89 | 1,09 | 58 | 0,75 | 6122 | 6250 | A | G |
| rs2899664 | RORA | MIXED | 3 | 1,01 | 0,95 | 1,07 | 0 | 0,79 | 6122 | 6250 | G | A |
| rs2899666 | RORA | MIXED | 3 | 1,08 | 0,99 | 1,18 | 43 | 0,09 | 6122 | 6250 | A | G |
| rs3027188 | PER1 | BREAST | 3 | 0,92 | 0,82 | 1,02 | 13 | 0,11 | 2688 | 3364 | G | C |
| rs3027279 | PER1 | MIXED | 3 | 0,98 | 0,91 | 1,06 | 14 | 0,63 | 5605 | 6292 | A | C |
| **rs339972** | **RORA** | **MIXED** | **3** | **1,08** | **1,01** | **1,15** | **0** | **0,02** | **6122** | **6250** | A | G |
| rs339996 | RORA | MIXED | 3 | 1,01 | 0,96 | 1,07 | 0 | 0,69 | 6122 | 6250 | A | G |
| rs339998 | RORA | MIXED | 3 | 1,01 | 0,96 | 1,07 | 0 | 0,61 | 6122 | 6250 | G | A |
| rs340023 | RORA | MIXED | 3 | 0,97 | 0,91 | 1,03 | 0 | 0,35 | 6122 | 6250 | G | A |
| rs340026 | RORA | BREAST | 2 | 1,11 | 0,98 | 1,25 | 0 | 0,1 | 2271 | 2316 | A | G |
| rs341365 | RORA | MIXED | 3 | 0,99 | 0,92 | 1,07 | 25 | 0,75 | 6122 | 6250 | G | A |
| rs341366 | RORA | MIXED | 3 | 0,99 | 0,93 | 1,04 | 0 | 0,62 | 6122 | 6250 | A | G |
| rs341382 | RORA | MIXED | 3 | 0,99 | 0,93 | 1,05 | 0 | 0,66 | 6122 | 6250 | A | G |
| rs341392 | RORA | MIXED | 3 | 1,03 | 0,97 | 1,1 | 0 | 0,32 | 6122 | 6250 | A | C |
| rs341399 | RORA | MIXED | 3 | 0,94 | 0,87 | 1,02 | 0 | 0,12 | 6122 | 6250 | G | A |
| rs341403 | RORA | MIXED | 3 | 1,05 | 0,99 | 1,12 | 0 | 0,09 | 6122 | 6250 | A | G |
| rs341459 | RORA | MIXED | 3 | 0,96 | 0,82 | 1,12 | 80 | 0,6 | 6122 | 6250 | A | G |
| rs356642 | NPAS2 | MIXED | 3 | 0,85 | 0,73 | 0,99 | 80 | 0,04 | 7198 | 10466 | G | A |
| rs356643 | NPAS2 | MIXED | 3 | 0,97 | 0,91 | 1,02 | 0 | 0,25 | 5605 | 6292 | A | G |
| rs356652 | NPAS2 | MIXED | 3 | 1,03 | 0,93 | 1,14 | 41 | 0,58 | 7679 | 10834 | A | C |
| **rs3739008** | **NPAS2** | **MIXED** | **5** | **1,06** | **1,01** | **1,11** | **0** | **0,02** | **8405** | **9892** | T | C |
| rs3743266 | RORA | MIXED | 3 | 0,98 | 0,93 | 1,04 | 0 | 0,51 | 6122 | 6250 | A | G |
| rs3744805 | NR1D1 | MIXED | 3 | 0,98 | 0,91 | 1,05 | 0 | 0,52 | 7679 | 10834 | G | A |
| **rs3749474** | **CLOCK** | **BREAST** | **2** | **0,86** | **0,76** | **0,98** | **0** | **0,02** | **1004** | **1098** | C | T |
| rs3750420 | RORB | MIXED | 4 | 1,01 | 0,96 | 1,07 | 0 | 0,63 | 6685 | 6869 | C | T |
| rs3754674 | NPAS2 | MIXED | 3 | 0,91 | 0,8 | 1,04 | 49 | 0,17 | 1873 | 3604 | G | C |
| rs3754675 | NPAS2 | BREAST | 2 | 1,16 | 0,99 | 1,36 | 0 | 0,08 | 6122 | 6250 | A | G |
| **rs3754677** | **NPAS2** | **MIXED** | **3** | 0,95 | **0,9** | **1** | **0** | **0,03** | **7836** | **12016** | C | T |
| rs3768984 | NPAS2 | MIXED | 4 | 1 | 0,95 | 1,05 | 0 | 0,86 | 7300 | 12090 | A | C |
| rs3768988 | NPAS2 | MIXED | 2 | 1,01 | 0,95 | 1,07 | 0 | 0,86 | 4887 | 3896 | A | G |
| rs3768991 | NPAS2 | MIXED | 4 | 0,99 | 0,94 | 1,05 | 0 | 0,82 | 6260 | 7464 | T | C |
| rs3789327 | ARNTL | MIXED | 6 | 1,01 | 0,97 | 1,05 | 39 | 0,59 | 12187 | 15986 | A | G |
| rs3792603 | CLOCK | MIXED | 3 | 0,97 | 0,91 | 1,03 | 0 | 0,31 | 6122 | 6250 | A | G |
| rs3811561 | NPAS2 | MIXED | 2 | 0,94 | 0,89 | 1 | 0 | 0,06 | 6553 | 9660 | G | T |
| rs3816358 | ARNTL | MIXED | 4 | 0,93 | 0,81 | 1,07 | 60 | 0,31 | 7144 | 7264 | C | A |
| rs3816360 | ARNTL | MIXED | 5 | 1 | 0,95 | 1,06 | 56 | 0,89 | 12575 | 14622 | G | A |
| rs3817444 | CLOCK | MIXED | 2 | 0,98 | 0,93 | 1,03 | 0 | 0,47 | 6553 | 9660 | C | A |
| **rs3820787** | **NPAS2** | **MIXED** | **3** | **0,89** | **0,81** | **0,97** | **0** | **0,007** | **1873** | **3604** | G | A |
| rs3824872 | CRY2 | MIXED | 3 | 1,01 | 0,95 | 1,06 | 0 | 0,84 | 7162 | 10876 | C | A |
| rs3849377 | NPAS2 | MIXED | 2 | 0,98 | 0,91 | 1,05 | 38 | 0,5 | 6553 | 9660 | G | A |
| rs3849381 | NPAS2 | MIXED | 5 | 1,02 | 0,97 | 1,07 | 0 | 0,41 | 8426 | 13264 | T | C |
| rs3888170 | NPAS2 | MIXED | 3 | 1,05 | 0,98 | 1,12 | 0 | 0,2 | 5605 | 6292 | A | G |
| rs3905275 | RORA | MIXED | 3 | 1 | 0,91 | 1,11 | 41 | 0,94 | 6122 | 6250 | A | C |
| rs4074920 | NPAS2 | MIXED | 3 | 1,02 | 0,94 | 1,1 | 29 | 0,71 | 6304 | 6342 | A | G |
| rs4090240 | RORB | MIXED | 3 | 1 | 0,9 | 1,1 | 50 | 0,95 | 6122 | 6250 | G | A |
| rs4238351 | RORA | MIXED | 3 | 1,01 | 0,95 | 1,06 | 0 | 0,82 | 6122 | 6250 | G | A |
| rs4594196 | RORA | MIXED | 3 | 1,03 | 0,96 | 1,11 | 0 | 0,39 | 6122 | 6250 | A | G |
| rs4663868 | PER2 | MIXED | 2 | 0,98 | 0,9 | 1,06 | 0 | 0,59 | 6553 | 9660 | G | A |
| rs4757144 | ARNTL | MIXED | 5 | 1 | 0,94 | 1,06 | 47 | 0,99 | 9596 | 13316 | A | G |
| rs4757151 | ARNTL | MIXED | 3 | 1 | 0,96 | 1,03 | 0 | 0,77 | 5025 | 5110 | G | A |
| rs4774371 | RORA | MIXED | 3 | 0,96 | 0,9 | 1,03 | 0 | 0,27 | 6122 | 6250 | G | A |
| rs4774381 | RORA | MIXED | 3 | 0,97 | 0,91 | 1,04 | 0 | 0,39 | 6122 | 6250 | A | G |
| rs4774386 | RORA | MIXED | 3 | 0,96 | 0,89 | 1,03 | 0 | 0,25 | 6122 | 6250 | A | G |
| rs4774388 | RORA | MIXED | 3 | 0,96 | 0,89 | 1,03 | 0 | 0,25 | 6122 | 6250 | A | G |
| rs4775292 | RORA | MIXED | 3 | 1,07 | 0,98 | 1,16 | 37 | 0,11 | 6122 | 6250 | G | A |
| rs4775340 | RORA | MIXED | 3 | 0,99 | 0,94 | 1,05 | 0 | 0,73 | 6122 | 6250 | G | A |
| rs4775351 | RORA | MIXED | 3 | 0,99 | 0,92 | 1,06 | 0 | 0,73 | 6122 | 6250 | A | G |
| rs4775352 | RORA | MIXED | 3 | 1,01 | 0,93 | 1,1 | 0 | 0,76 | 6122 | 6250 | A | C |
| rs4775355 | RORA | MIXED | 3 | 0,96 | 0,83 | 1,1 | 71 | 0,55 | 6122 | 6250 | A | G |
| rs4775362 | RORA | MIXED | 3 | 1 | 0,94 | 1,07 | 0 | 0,93 | 6122 | 6250 | A | G |
| rs4775369 | RORA | MIXED | 3 | 0,97 | 0,92 | 1,03 | 0 | 0,36 | 6122 | 6250 | A | G |
| rs4775371 | RORA | MIXED | 3 | 0,97 | 0,91 | 1,04 | 0 | 0,43 | 6122 | 6250 | G | A |
| rs4794826 | NR1D1 | MIXED | 3 | 1,06 | 0,97 | 1,16 | 0 | 0,21 | 6122 | 6250 | C | T |
| rs4851377 | NPAS2 | MIXED | 4 | 1 | 0,92 | 1,08 | 0 | 0,95 | 6869 | 8680 | T | C |
| **rs4851384** | **NPAS2** | **MIXED** | **3** | **0,91** | **0,83** | **0,99** | **0** | **0,02** | **7679** | **10834** | G | A |
| rs4851390 | NPAS2 | MIXED | 5 | 1 | 0,94 | 1,07 | 27 | 0,97 | 8426 | 13264 | A | G |
| rs4851392 | NPAS2 | MIXED | 2 | 0,91 | 0,79 | 1,06 | 12 | 0,22 | 747 | 2430 | G | A |
| rs4864540 | CLOCK | MIXED | 3 | 1,03 | 0,96 | 1,09 | 0 | 0,42 | 5605 | 6292 | G | A |
| rs5757037 | CSNK1E | MIXED | 4 | 0,98 | 0,93 | 1,03 | 0 | 0,36 | 6867 | 6961 | C | T |
| rs6001093 | CSNK1E | MIXED | 5 | 1 | 0,95 | 1,05 | 0 | 0,96 | 9125 | 13314 | T | C |
| rs6431590 | PER2 | MIXED | 2 | 1,01 | 0,96 | 1,06 | 0 | 0,76 | 6553 | 9660 | A | G |
| rs6486121 | ARNTL | MIXED | 4 | 1 | 0,96 | 1,05 | 0 | 0,98 | 8288 | 12050 | T | C |
| rs6486122 | ARNTL | MIXED | 3 | 0,97 | 0,94 | 1,01 | 2 | 0,11 | 6195 | 5162 | A | G |
| rs6494217 | RORA | MIXED | 3 | 1 | 0,94 | 1,07 | 0 | 0,89 | 6122 | 6250 | G | A |
| rs6494219 | RORA | MIXED | 3 | 0,98 | 0,92 | 1,04 | 0 | 0,52 | 6122 | 6250 | G | A |
| rs6494221 | RORA | MIXED | 3 | 1 | 0,93 | 1,06 | 0 | 0,91 | 6122 | 6250 | A | C |
| rs6494229 | RORA | MIXED | 3 | 0,99 | 0,93 | 1,05 | 14 | 0,72 | 6122 | 6250 | G | A |
| rs6494232 | RORA | MIXED | 3 | 0,94 | 0,84 | 1,04 | 23 | 0,23 | 6122 | 6250 | A | G |
| rs6719437 | NPAS2 | MIXED | 2 | 1,07 | 0,95 | 1,19 | 0 | 0,27 | 1264 | 2388 | A | G |
| rs6719533 | NPAS2 | MIXED | 3 | 1,02 | 0,94 | 1,11 | 0 | 0,59 | 1873 | 3604 | A | G |
| rs6725296 | NPAS2 | BREAST | 2 | 1 | 0,89 | 1,12 | 0 | 0,97 | 1735 | 2390 | G | A |
| rs6729727 | NPAS2 | MIXED | 2 | 1 | 0,92 | 1,09 | 12 | 0,98 | 6553 | 9660 | A | C |
| rs6738097 | NPAS2 | MIXED | 2 | 0,98 | 0,92 | 1,06 | 0 | 0,67 | 6553 | 9660 | A | G |
| rs6740935 | NPAS2 | MIXED | 3 | 1,04 | 0,97 | 1,12 | 45 | 0,23 | 7162 | 10876 | G | A |
| rs6747755 | NPAS2 | BREAST | 2 | 0,96 | 0,87 | 1,07 | 0 | 0,5 | 1735 | 2390 | G | A |
| rs6850524 | CLOCK | BREAST | 2 | 0,95 | 0,84 | 1,07 | 0 | 0,39 | 1086 | 1285 | G | C |
| rs697673 | PER3 | MIXED | 3 | 1,03 | 0,91 | 1,16 | 60 | 0,68 | 5605 | 6292 | G | A |
| rs697686 | PER3 | MIXED | 2 | 0,99 | 0,91 | 1,07 | 61 | 0,83 | 6553 | 9660 | G | A |
| rs697690 | PER3 | BREAST | 2 | 0,98 | 0,82 | 1,16 | 68 | 0,78 | 1735 | 2390 | A | G |
| rs7022435 | RORB | BREAST | 2 | 1 | 0,9 | 1,11 | 0 | 0,98 | 1689 | 1793 | G | A |
| rs7032677 | RORB | MIXED | 3 | 1,03 | 0,97 | 1,08 | 0 | 0,35 | 6122 | 6250 | G | A |
| rs7037043 | RORB | MIXED | 3 | 1,01 | 0,96 | 1,07 | 0 | 0,62 | 6122 | 6250 | A | G |
| rs707455 | PER3 | MIXED | 3 | 1 | 0,9 | 1,11 | 52 | 0,94 | 5605 | 6292 | G | A |
| rs707467 | PER3 | MIXED | 4 | 1 | 0,93 | 1,07 | 0 | 1 | 6200 | 6324 | T | C |
| rs7123390 | CRY2 | MIXED | 3 | 0,97 | 0,84 | 1,12 | 48 | 0,64 | 2022 | 2180 | G | A |
| rs7126303 | ARNTL | MIXED | 2 | 1,01 | 0,96 | 1,06 | 0 | 0,73 | 6553 | 9660 | A | G |
| rs7162388 | RORA | MIXED | 2 | 1,07 | 0,95 | 1,21 | 0 | 0,28 | 4977 | 5108 | G | A |
| rs7164773 | RORA | MIXED | 3 | 1,09 | 0,96 | 1,22 | 68 | 0,18 | 6122 | 6250 | G | A |
| rs7166448 | RORA | MIXED | 3 | 0,93 | 0,85 | 1,02 | 0 | 0,11 | 6122 | 6250 | G | A |
| rs7168782 | RORA | MIXED | 3 | 1 | 0,91 | 1,09 | 27 | 0,93 | 6122 | 6250 | C | A |
| **rs7172348** | **RORA** | **MIXED** | **3** | **0,93** | **0,87** | **0,99** | **0** | **0,03** | **6122** | **6250** | A | G |
| rs7175883 | RORA | MIXED | 2 | 0,95 | 0,8 | 1,12 | 0 | 0,51 | 4977 | 5108 | G | A |
| rs7177611 | RORA | MIXED | 3 | 0,99 | 0,9 | 1,1 | 53 | 0,87 | 6122 | 6250 | G | A |
| rs7181803 | RORA | MIXED | 2 | 1,06 | 0,9 | 1,26 | 0 | 0,47 | 4977 | 5108 | A | G |
| rs7183955 | RORA | MIXED | 3 | 0,98 | 0,9 | 1,08 | 33 | 0,72 | 6122 | 6250 | A | C |
| rs726955 | RORA | MIXED | 3 | 0,98 | 0,92 | 1,04 | 0 | 0,49 | 6122 | 6250 | G | A |
| rs7297614 | CRY1 | MIXED | 5 | 0,98 | 0,94 | 1,03 | 0 | 0,52 | 7568 | 8730 | T | C |
| rs7302060 | TIMELESS | MIXED | 3 | 0,99 | 0,96 | 1,02 | 0 | 0,43 | 4847 | 4007 | A | G |
| rs732375 | NPAS2 | MIXED | 2 | 1,01 | 0,84 | 1,21 | 80 | 0,91 | 16524 | 31990 | T | A |
| rs7564495 | NPAS2 | MIXED | 2 | 0,98 | 0,87 | 1,11 | 0 | 0,76 | 1264 | 2388 | C | T |
| rs7565018 | NPAS2 | MIXED | 3 | 0,99 | 0,93 | 1,05 | 0 | 0,76 | 6304 | 6342 | A | G |
| **rs7581886** | **NPAS2** | **BREAST** | **2** | **0,86** | **0,75** | **0,98** | **0** | **0,03** | **1735** | **2390** | A | G |
| rs7584376 | PER2 | MIXED | 3 | 1,02 | 0,96 | 1,08 | 0 | 0,5 | 5605 | 6292 | C | A |
| rs7598826 | NPAS2 | MIXED | 5 | 0,98 | 0,94 | 1,02 | 0 | 0,27 | 8426 | 13264 | A | G |
| **rs7602358** | **PER2** | **MIXED** | **6** | **1,08** | **1,02** | **1,14** | **0** | **0,005** | **8190** | **9358** | T | G |
| rs7605570 | NPAS2 | MIXED | 3 | 0,97 | 0,92 | 1,02 | 3 | 0,22 | 7162 | 10876 | C | A |
| rs7698022 | CLOCK | MIXED | 5 | 0,97 | 0,86 | 1,1 | 57 | 0,68 | 2834 | 3087 | T | G |
| rs774027 | TIMELESS | MIXED | 3 | 1,02 | 0,94 | 1,1 | 0 | 0,67 | 2572 | 3654 | T | A |
| rs774047 | TIMELESS | MIXED | 4 | 1,01 | 0,96 | 1,07 | 0 | 0,7 | 6442 | 7556 | C | T |
| rs774049 | TIMELESS | MIXED | 2 | 0,95 | 0,83 | 1,1 | 0 | 0,52 | 4977 | 5108 | G | A |
| rs782903 | RORA | MIXED | 3 | 0,99 | 0,94 | 1,05 | 0 | 0,79 | 6122 | 6250 | A | C |
| rs782907 | RORA | MIXED | 3 | 0,95 | 0,89 | 1 | 0 | 0,06 | 6122 | 6250 | G | A |
| rs782910 | RORA | MIXED | 3 | 1,01 | 0,95 | 1,08 | 0 | 0,7 | 6122 | 6250 | G | A |
| rs782926 | RORA | MIXED | 3 | 1 | 0,94 | 1,06 | 0 | 0,91 | 6122 | 6250 | G | A |
| rs782938 | RORA | MIXED | 3 | 0,98 | 0,92 | 1,03 | 0 | 0,41 | 6122 | 6250 | A | G |
| rs782944 | RORA | MIXED | 3 | 0,99 | 0,86 | 1,13 | 74 | 0,86 | 6122 | 6250 | A | C |
| rs782947 | RORA | MIXED | 3 | 1 | 0,93 | 1,07 | 0 | 0,96 | 6122 | 6250 | A | G |
| rs7848922 | RORB | MIXED | 3 | 0,94 | 0,89 | 1,01 | 7 | 0,08 | 6122 | 6250 | A | G |
| rs7867494 | RORB | MIXED | 3 | 0,95 | 0,87 | 1,04 | 37 | 0,28 | 6122 | 6250 | A | G |
| rs7924734 | ARNTL | MIXED | 2 | 0,99 | 0,92 | 1,07 | 45 | 0,82 | 6553 | 9660 | A | G |
| rs7942486 | ARNTL | MIXED | 3 | 0,96 | 0,9 | 1,01 | 0 | 0,14 | 5605 | 6292 | A | C |
| rs7947951 | ARNTL | MIXED | 2 | 0,99 | 0,96 | 1,03 | 0 | 0,58 | 3930 | 4395 | G | A |
| rs7949336 | ARNTL | MIXED | 3 | 0,98 | 0,87 | 1,11 | 43 | 0,49 | 5134 | 6290 | A | G |
| rs7950226 | ARNTL | MIXED | 7 | 1,02 | 0,96 | 1,09 | 42 | 0,52 | 8799 | 10574 | G | A |
| rs7951225 | CRY2 | MIXED | 2 | 1,08 | 0,85 | 1,37 | 55 | 0,54 | 1264 | 2388 | A | T |
| rs8023252 | RORA | MIXED | 3 | 0,99 | 0,93 | 1,04 | 0 | 0,6 | 6122 | 6250 | A | C |
| rs8024629 | RORA | MIXED | 3 | 0,96 | 0,85 | 1,07 | 54 | 0,45 | 6122 | 6250 | C | A |
| rs8027032 | RORA | MIXED | 3 | 0,97 | 0,91 | 1,04 | 0 | 0,38 | 6122 | 6250 | A | G |
| rs8027829 | RORA | MIXED | 4 | 1,01 | 0,95 | 1,07 | 0 | 0,79 | 6122 | 6250 | G | A |
| rs8031801 | RORA | MIXED | 3 | 1,05 | 0,98 | 1,12 | 2 | 0,16 | 6122 | 6250 | G | A |
| rs8032023 | RORA | MIXED | 3 | 0,99 | 0,93 | 1,04 | 0 | 0,67 | 6122 | 6250 | A | G |
| rs8034880 | RORA | MIXED | 3 | 1 | 0,89 | 1,13 | 38 | 0,99 | 6122 | 6250 | A | G |
| rs8034950 | RORA | MIXED | 3 | 0,98 | 0,92 | 1,04 | 0 | 0,45 | 6122 | 6250 | A | G |
| rs8036866 | RORA | MIXED | 3 | 1,05 | 0,96 | 1,15 | 0 | 0,31 | 6122 | 6250 | G | A |
| rs8037669 | RORA | MIXED | 3 | 1,02 | 0,96 | 1,07 | 0 | 0,59 | 6122 | 6250 | G | A |
| rs8038077 | RORA | MIXED | 3 | 1,06 | 0,9 | 1,23 | 48 | 0,5 | 6122 | 6250 | A | G |
| rs8041061 | RORA | MIXED | 3 | 0,98 | 0,93 | 1,04 | 0 | 0,52 | 6122 | 6250 | A | C |
| rs8041381 | RORA | MIXED | 3 | 0,98 | 0,87 | 1,1 | 69 | 0,7 | 6122 | 6250 | A | G |
| rs8042149 | RORA | MIXED | 3 | 0,97 | 0,92 | 1,02 | 0 | 0,26 | 6122 | 6250 | C | A |
| rs8043356 | RORA | MIXED | 3 | 1,04 | 0,97 | 1,12 | 0 | 0,29 | 6122 | 6250 | G | A |
| rs809736 | RORA | MIXED | 3 | 0,98 | 0,92 | 1,04 | 0 | 0,53 | 6553 | 9660 | A | G |
| rs875994 | PER3 | MIXED | 3 | 1,04 | 0,97 | 1,11 | 21 | 0,3 | 6553 | 9660 | A | G |
| rs880625 | RORA | MIXED | 3 | 0,97 | 0,9 | 1,04 | 0 | 0,37 | 6122 | 6250 | A | G |
| rs880626 | RORA | MIXED | 3 | 1 | 0,94 | 1,06 | 0 | 0,99 | 6122 | 6250 | G | A |
| rs885747 | PER1 | PROSTATE | 2 | 1,03 | 0,85 | 1,25 | 55 | 0,75 | 1446 | 2480 | C | G |
| **rs895520** | **NPAS2** | **MIXED** | **4** | 1,08 | **1,03** | **1,13** | **0** | **0,001** | **7817** | **12048** | G | A |
| rs895521 | NPAS2 | MIXED | 6 | 0,95 | 0,9 | 1,01 | 29 | 0,13 | 9747 | 13942 | G | A |
| rs919000 | RORA | MIXED | 3 | 0,98 | 0,91 | 1,04 | 0 | 0,47 | 6122 | 6250 | A | G |
| rs9312661 | CLOCK | MIXED | 2 | 0,95 | 0,91 | 1 | 0 | 0,07 | 6553 | 9660 | G | A |
| rs934945 | PER2 | MIXED | 5 | 0,98 | 0,94 | 1,03 | 0 | 0,53 | 9619 | 12919 | G | A |
| **rs935401** | **NPAS2** | **BREAST** | **2** | **1,11** | **1,02** | **1,22** | **0** | **0,02** | **1735** | **2390** | A | T |
| rs940222 | RORA | MIXED | 3 | 1 | 0,94 | 1,07 | 0 | 0,96 | 6122 | 6250 | A | C |
| rs940224 | RORA | MIXED | 3 | 0,99 | 0,94 | 1,05 | 0 | 0,8 | 6122 | 6250 | G | A |
| rs965519 | NPAS2 | MIXED | 5 | 1,04 | 0,97 | 1,13 | 45 | 0,28 | 9596 | 13316 | A | G |
| rs968357 | RORB | MIXED | 3 | 0,98 | 0,92 | 1,05 | 0 | 0,62 | 6122 | 6250 | A | G |
| rs969485 | ARNTL | MIXED | 6 | 1,01 | 0,93 | 1,09 | 54 | 0,83 | 8989 | 13883 | A | G |
| rs974828 | RORA | MIXED | 3 | 0,98 | 0,83 | 1,15 | 63 | 0,78 | 6122 | 6250 | G | A |
| rs9788704 | RORA | MIXED | 3 | 1,03 | 0,94 | 1,12 | 38 | 0,55 | 6122 | 6250 | G | A |
| rs9806453 | RORA | BREAST | 2 | 1,02 | 0,9 | 1,15 | 25 | 0,78 | 6122 | 6250 | C | A |
| rs9920560 | RORA | MIXED | 3 | 1,06 | 0,96 | 1,18 | 31 | 0,23 | 6122 | 6250 | C | A |

| **BREAST CANCER SUBGROUP** | | | | | | | | | | | | |
| --- | --- | --- | --- | --- | --- | --- | --- | --- | --- | --- | --- | --- |
| **SNP ID** | **GENE** | **CANCER** | **STUDIES** | **OR** | **CI Lower** | **CI Upper** | **I ^2^ %** | **P value** | **Subgroup** | **Cases** | | **Controls** |
| 4/5 repeats | PER3 | BREAST | 3 | 1,08 | 0,97 | 1,2 | 0 | 0,19 | OVERALL | 2182 | | 2286 |
| rs1012477 | PER3 | BREAST | 3 | 0,92 | 0,82 | 1,02 | 0 | 0,13 | OVERALL | 2642 | | 2767 |
| rs10162630 | RORA | BREAST | 2 | 1,03 | 0,93 | 1,14 | 0 | 0,52 | OVERALL | 2271 | | 2316 |
| rs10165970 | NPAS2 | BREAST | 3 | 1,13 | 1,04 | 1,23 | 0 | 0,003 | OVERALL | 4437 | | 8116 |
| rs1044245 | PER3 | BREAST | 2 | 1,09 | 0,95 | 1,25 | 0 | 0,23 | OVERALL | 1754 | | 2358 |
| rs10462018 | PER3 | BREAST | 2 | 0,97 | 0,89 | 1,05 | 0 | 0,41 | OVERALL | 3828 | | 6900 |
| rs10462020 | PER3 | BREAST | 2 | 1,06 | 0,99 | 1,15 | 0 | 0,11 | OVERALL | 3828 | | 6900 |
| rs10462021 | PER3 | BREAST | 2 | 1,07 | 0,99 | 1,15 | 0 | 0,09 | OVERALL | 3828 | | 6900 |
| rs10462023 | PER2 | BREAST | 4 | 1 | 0,93 | 1,07 | 33 | 0,91 | OVERALL | 5459 | | 9130 |
| rs10462028 | CLOCK | BREAST | 2 | 1,3 | 0,66 | 2,57 | 98 | 0,45 | OVERALL | 2148 | | 2188 |
| rs10519070 | RORA | BREAST | 2 | 0,96 | 0,84 | 1,09 | 0 | 0,51 | OVERALL | 2271 | | 2316 |
| rs10519097 | RORA | BREAST | 2 | 0,85 | 0,75 | 0,96 | 0 | 0,008 | OVERALL | 2271 | | 2316 |
| rs10519099 | RORA | BREAST | 2 | 0,92 | 0,83 | 1,02 | 0 | 0,1 | OVERALL | 2271 | | 2316 |
| rs10519111 | RORA | BREAST | 2 | 1,02 | 0,89 | 1,17 | 24 | 0,77 | OVERALL | 2271 | | 2316 |
| rs10766074 | ARNTL | BREAST | 2 | 1 | 0,88 | 1,12 | 0 | 0,96 | OVERALL | 1735 | | 2390 |
| rs10781235 | RORB | BREAST | 2 | 0,95 | 0,85 | 1,06 | 0 | 0,35 | OVERALL | 2271 | | 2316 |
| rs10832027 | ARNTL | BREAST | 3 | 1,02 | 0,96 | 1,08 | 0 | 0,59 | OVERALL | 3311 | | 6942 |
| rs10838524 | CRY2 | BREAST | 2 | 0,98 | 0,83 | 1,17 | 76 | 0,83 | OVERALL | 2148 | | 2188 |
| rs10838527 | CRY2 | BREAST | 2 | 0,95 | 0,8 | 1,13 | 0 | 0,56 | OVERALL | 1754 | | 2358 |
| rs10869410 | RORB | BREAST | 2 | 1,04 | 0,75 | 1,43 | 81 | 0,81 | OVERALL | 2271 | | 2316 |
| rs10869417 | RORB | BREAST | 2 | 0,9 | 0,81 | 1 | 0 | 0,06 | OVERALL | 2271 | | 2316 |
| rs11022742 | ARNTL | BREAST | 2 | 1,01 | 0,9 | 1,14 | 0 | 0,83 | OVERALL | 1754 | | 2358 |
| rs11022778 | ARNTL | BREAST | 3 | 0,98 | 0,91 | 1,06 | 27 | 0,6 | OVERALL | 4437 | | 8116 |
| rs11038689 | CRY2 | BREAST | 2 | 0,89 | 0,76 | 1,03 | 0 | 0,12 | OVERALL | 1004 | | 1098 |
| rs11071587 | RORA | BREAST | 2 | 0,95 | 0,86 | 1,05 | 0 | 0,32 | OVERALL | 2271 | | 2316 |
| rs11071588 | RORA | BREAST | 2 | 1,07 | 0,97 | 1,18 | 0 | 0,15 | OVERALL | 2271 | | 2316 |
| rs11113179 | CRY1 | BREAST | 4 | 1,07 | 0,94 | 1,21 | 21 | 0,31 | OVERALL | 3833 | | 4506 |
| rs11123857 | NPAS2 | BREAST | 4 | 1,01 | 0,89 | 1,15 | 77 | 0,87 | OVERALL | 5459 | | 9130 |
| rs11133373 | CLOCK | BREAST | 2 | 1,04 | 0,91 | 1,19 | 28 | 0,6 | OVERALL | 1172 | | 1835 |
| rs11133375 | CLOCK | BREAST | 2 | 1,06 | 0,94 | 1,18 | 0 | 0,34 | OVERALL | 1754 | | 2358 |
| rs11133391 | CLOCK | BREAST | 2 | 0,94 | 0,78 | 1,14 | 63 | 0,52 | OVERALL | 1567 | | 1653 |
| rs11171846 | TIMELESS | BREAST | 2 | 0,98 | 0,84 | 1,14 | 16 | 0,75 | OVERALL | 2271 | | 2316 |
| rs1159814 | RORA | BREAST | 2 | 1,02 | 0,92 | 1,12 | 0 | 0,75 | OVERALL | 2271 | | 2316 |
| rs11605924 | CRY2 | BREAST | 2 | 0,98 | 0,85 | 1,12 | 0 | 0,73 | OVERALL | 1586 | | 1621 |
| rs1160694 | RORA | BREAST | 2 | 0,99 | 0,83 | 1,18 | 65 | 0,88 | OVERALL | 2271 | | 2316 |
| rs11629597 | RORA | BREAST | 2 | 0,97 | 0,86 | 1,1 | 21 | 0,67 | OVERALL | 2271 | | 2316 |
| rs11630018 | RORA | BREAST | 2 | 1 | 0,87 | 1,16 | 0 | 0,95 | OVERALL | 2271 | | 2316 |
| rs11630062 | RORA | BREAST | 2 | 0,96 | 0,77 | 1,2 | 51 | 0,73 | OVERALL | 2271 | | 2316 |
| rs11630227 | RORA | BREAST | 2 | 0,99 | 0,9 | 1,1 | 5 | 0,9 | OVERALL | 2271 | | 2316 |
| rs11631432 | RORA | BREAST | 2 | 0,95 | 0,86 | 1,05 | 2 | 0,33 | OVERALL | 2271 | | 2316 |
| rs11634318 | RORA | BREAST | 2 | 1,04 | 0,87 | 1,23 | 0 | 0,67 | OVERALL | 2271 | | 2316 |
| rs11635314 | RORA | BREAST | 2 | 1,03 | 0,9 | 1,17 | 13 | 0,7 | OVERALL | 2271 | | 2316 |
| rs11695472 | PER2 | BREAST | 2 | 0,99 | 0,87 | 1,13 | 0 | 0,9 | OVERALL | 1708 | | 1761 |
| rs11894535 | PER2 | BREAST | 2 | 1 | 0,9 | 1,13 | 0 | 0,94 | OVERALL | 1562 | | 2190 |
| rs11894671 | NPAS2 | BREAST | 2 | 1,07 | 0,99 | 1,15 | 0 | 0,07 | OVERALL | 3311 | | 6942 |
| rs11932595 | CLOCK | BREAST | 4 | 0,99 | 0,87 | 1,13 | 62 | 0,93 | OVERALL | 3357 | | 3601 |
| rs11943456 | CLOCK | BREAST | 2 | 1,16 | 1,02 | 1,32 | 0 | 0,02 | OVERALL | 1754 | | 2358 |
| rs12101478 | RORA | BREAST | 2 | 1,07 | 0,95 | 1,2 | 0 | 0,24 | OVERALL | 2271 | | 2316 |
| rs12315175 | CRY1 | BREAST | 2 | 0,97 | 0,81 | 1,16 | 54 | 0,76 | OVERALL | 1754 | | 2358 |
| rs12324440 | RORA | BREAST | 2 | 0,98 | 0,86 | 1,13 | 40 | 0,82 | OVERALL | 2271 | | 2316 |
| rs12421530 | ARNTL | BREAST | 2 | 1 | 0,91 | 1,1 | 0 | 0,94 | OVERALL | 1735 | | 2390 |
| rs12438866 | RORA | BREAST | 2 | 0,93 | 0,73 | 1,17 | 78 | 0,52 | OVERALL | 2271 | | 2316 |
| rs12440095 | RORA | BREAST | 2 | 1 | 0,88 | 1,14 | 0 | 0,94 | OVERALL | 2271 | | 2316 |
| rs12443239 | RORA | BREAST | 2 | 0,92 | 0,69 | 1,22 | 85 | 0,55 | OVERALL | 2271 | | 2316 |
| rs12472321 | NPAS2 | BREAST | 2 | 0,93 | 0,84 | 1,03 | 0 | 0,18 | OVERALL | 1735 | | 2390 |
| rs12591650 | RORA | BREAST | 2 | 0,95 | 0,84 | 1,08 | 0 | 0,44 | OVERALL | 2271 | | 2316 |
| rs12594972 | RORA | BREAST | 2 | 0,9 | 0,78 | 1,03 | 0 | 0,13 | OVERALL | 2271 | | 2316 |
| rs12712083 | NPAS2 | BREAST | 3 | 0,96 | 0,84 | 1,11 | 64 | 0,59 | OVERALL | 2880 | | 3532 |
| rs12712085 | NPAS2 | BREAST | 2 | 0,89 | 0,81 | 0,97 | 0 | 0,01 | OVERALL | 1735 | | 2390 |
| rs12821586 | CRY1 | BREAST | 2 | 1,06 | 0,74 | 1,54 | 77 | 0,75 | OVERALL | 2271 | | 2316 |
| rs12909890 | RORA | BREAST | 2 | 0,96 | 0,88 | 1,06 | 0 | 0,47 | OVERALL | 2271 | | 2316 |
| rs12941497 | NR1D1 | BREAST | 2 | 0,97 | 0,87 | 1,07 | 0 | 0,52 | OVERALL | 2271 | | 2316 |
| rs13025524 | NPAS2 | BREAST | 3 | 0,93 | 0,86 | 1,01 | 0 | 0,09 | OVERALL | 2880 | | 3532 |
| rs13026599 | NPAS2 | BREAST | 2 | 1,05 | 0,94 | 1,18 | 0 | 0,4 | OVERALL | 1735 | | 2390 |
| rs1323369 | RORB | BREAST | 2 | 0,95 | 0,79 | 1,16 | 67 | 0,64 | OVERALL | 2271 | | 2316 |
| rs1327836 | RORB | BREAST | 2 | 0,98 | 0,87 | 1,1 | 0 | 0,73 | OVERALL | 2271 | | 2316 |
| rs1327837 | RORB | BREAST | 2 | 0,93 | 0,85 | 1,03 | 0 | 0,16 | OVERALL | 2271 | | 2316 |
| rs13329238 | RORA | BREAST | 2 | 1,08 | 0,95 | 1,23 | 39 | 0,26 | OVERALL | 2271 | | 2316 |
| rs13329643 | RORA | BREAST | 2 | 0,94 | 0,83 | 1,08 | 0 | 0,4 | OVERALL | 2271 | | 2316 |
| rs1351545 | RORA | BREAST | 2 | 0,98 | 0,83 | 1,15 | 57 | 0,76 | OVERALL | 2271 | | 2316 |
| rs1351546 | RORA | BREAST | 2 | 0,96 | 0,87 | 1,06 | 0 | 0,47 | OVERALL | 2271 | | 2316 |
| rs135757 | CSNK1E | BREAST | 3 | 0,91 | 0,82 | 1,03 | 0 | 0,13 | OVERALL | 1790 | | 1948 |
| rs1369481 | NPAS2 | BREAST | 3 | 0,93 | 0,84 | 1,03 | 54 | 0,15 | OVERALL | 4437 | | 8116 |
| rs1401417 | CRY2 | BREAST | 6 | 1,02 | 0,94 | 1,1 | 20 | 0,7 | OVERALL | 4922 | | 5899 |
| rs1403737 | RORA | BREAST | 2 | 0,97 | 0,87 | 1,07 | 0 | 0,53 | OVERALL | 2271 | | 2316 |
| rs1407845 | RORB | BREAST | 2 | 0,92 | 0,76 | 1,12 | 71 | 0,42 | OVERALL | 2271 | | 2316 |
| rs1410226 | RORB | BREAST | 2 | 1,02 | 0,88 | 1,19 | 0 | 0,79 | OVERALL | 2271 | | 2316 |
| rs1410227 | RORB | BREAST | 2 | 0,98 | 0,86 | 1,11 | 0 | 0,73 | OVERALL | 2271 | | 2316 |
| rs1425287 | RORA | BREAST | 2 | 0,98 | 0,87 | 1,09 | 0 | 0,67 | OVERALL | 2271 | | 2316 |
| rs1437543 | RORA | BREAST | 2 | 1,07 | 0,96 | 1,19 | 0 | 0,21 | OVERALL | 2271 | | 2316 |
| rs1437547 | RORA | BREAST | 2 | 0,98 | 0,72 | 1,35 | 78 | 0,91 | OVERALL | 2271 | | 2316 |
| rs1437550 | RORA | BREAST | 2 | 0,99 | 0,88 | 1,11 | 0 | 0,86 | OVERALL | 2271 | | 2316 |
| rs1442849 | PER1 | BREAST | 2 | 1,05 | 0,93 | 1,18 | 0 | 0,41 | OVERALL | 1754 | | 2358 |
| rs1465812 | RORA | BREAST | 2 | 0,96 | 0,87 | 1,07 | 0 | 0,49 | OVERALL | 2271 | | 2316 |
| rs1482049 | RORA | BREAST | 2 | 1 | 0,89 | 1,13 | 12 | 0,99 | OVERALL | 2271 | | 2316 |
| rs1482058 | RORA | BREAST | 2 | 1 | 0,9 | 1,11 | 0 | 1 | OVERALL | 2271 | | 2316 |
| rs1534891 | CSNK1E | BREAST | 3 | 0,97 | 0,86 | 1,1 | 35 | 0,67 | OVERALL | 4391 | | 7519 |
| rs1542178 | NPAS2 | BREAST | 2 | 0,9 | 0,82 | 1 | 0 | 0,05 | OVERALL | 2271 | | 2316 |
| rs1542179 | NPAS2 | BREAST | 2 | 0,95 | 0,85 | 1,05 | 49 | 0,28 | OVERALL | 3828 | | 6900 |
| rs1562313 | NPAS2 | BREAST | 2 | 0,98 | 0,9 | 1,06 | 0 | 0,57 | OVERALL | 3311 | | 6942 |
| rs1562438 | ARNTL | BREAST | 2 | 1,01 | 0,92 | 1,12 | 0 | 0,8 | OVERALL | 1735 | | 2390 |
| rs1589702 | RORA | BREAST | 2 | 0,97 | 0,79 | 1,18 | 72 | 0,73 | OVERALL | 2271 | | 2316 |
| rs1589703 | RORA | BREAST | 2 | 1 | 0,9 | 1,12 | 7 | 0,95 | OVERALL | 2271 | | 2316 |
| rs1632660 | RORA | BREAST | 2 | 0,99 | 0,88 | 1,11 | 0 | 0,83 | OVERALL | 2271 | | 2316 |
| rs16912751 | ARNTL | BREAST | 2 | 1,03 | 0,88 | 1,19 | 2 | 0,74 | OVERALL | 3828 | | 6900 |
| rs16943087 | RORA | BREAST | 2 | 1,12 | 0,98 | 1,27 | 0 | 0,09 | OVERALL | 2271 | | 2316 |
| rs16943318 | RORA | BREAST | 2 | 1,02 | 0,92 | 1,14 | 0 | 0,66 | OVERALL | 2271 | | 2316 |
| rs16943453 | RORA | BREAST | 2 | 1,03 | 0,9 | 1,17 | 14 | 0,68 | OVERALL | 2271 | | 2316 |
| rs16943579 | RORA | BREAST | 2 | 0,93 | 0,71 | 1,21 | 60 | 0,58 | OVERALL | 2271 | | 2316 |
| rs17020663 | NPAS2 | BREAST | 2 | 1 | 0,87 | 1,14 | 0 | 0,97 | OVERALL | 1735 | | 2390 |
| rs17024869 | NPAS2 | BREAST | 2 | 0,81 | 0,7 | 0,94 | 0 | 0,006 | OVERALL | 2271 | | 2316 |
| rs17024926 | NPAS2 | BREAST | 3 | 1,05 | 0,99 | 1,11 | 0 | 0,13 | OVERALL | 4391 | | 7519 |
| rs17025005 | NPAS2 | BREAST | 2 | 0,9 | 0,81 | 1,01 | 0 | 0,08 | OVERALL | 1735 | | 2390 |
| rs17191442 | RORA | BREAST | 2 | 1,07 | 0,93 | 1,24 | 0 | 0,34 | OVERALL | 2271 | | 2316 |
| rs17191554 | RORA | BREAST | 2 | 1,04 | 0,65 | 1,69 | 87 | 0,86 | OVERALL | 2271 | | 2316 |
| rs17204573 | RORA | BREAST | 2 | 1,01 | 0,88 | 1,17 | 0 | 0,88 | OVERALL | 2271 | | 2316 |
| rs17204698 | RORA | BREAST | 2 | 1,03 | 0,92 | 1,15 | 0 | 0,64 | OVERALL | 2271 | | 2316 |
| rs17204770 | RORA | BREAST | 2 | 1,02 | 0,93 | 1,13 | 0 | 0,66 | OVERALL | 2271 | | 2316 |
| rs17237367 | RORA | BREAST | 2 | 0,97 | 0,78 | 1,21 | 73 | 0,78 | OVERALL | 2271 | | 2316 |
| rs17237486 | RORA | BREAST | 2 | 0,96 | 0,83 | 1,1 | 39 | 0,54 | OVERALL | 2271 | | 2316 |
| rs17237521 | RORA | BREAST | 2 | 0,91 | 0,81 | 1,03 | 0 | 0,13 | OVERALL | 2271 | | 2316 |
| rs17237563 | RORA | BREAST | 2 | 1,07 | 0,86 | 1,32 | 64 | 0,56 | OVERALL | 2271 | | 2316 |
| rs17270188 | RORA | BREAST | 2 | 1,12 | 1,02 | 1,24 | 0 | 0,02 | OVERALL | 2271 | | 2316 |
| rs17270216 | RORA | BREAST | 2 | 0,89 | 0,76 | 1,03 | 43 | 0,11 | OVERALL | 2271 | | 2316 |
| rs17303097 | RORA | BREAST | 2 | 0,97 | 0,87 | 1,08 | 0 | 0,58 | OVERALL | 2271 | | 2316 |
| rs17452383 | ARNTL | BREAST | 2 | 0,99 | 0,86 | 1,13 | 5 | 0,87 | OVERALL | 1735 | | 2390 |
| rs17612183 | RORB | BREAST | 2 | 0,93 | 0,77 | 1,13 | 48 | 0,48 | OVERALL | 2271 | | 2316 |
| rs17654772 | NPAS2 | BREAST | 2 | 1,1 | 0,94 | 1,29 | 43 | 0,22 | OVERALL | 1754 | | 2358 |
| rs17776421 | CLOCK | BREAST | 2 | 0,95 | 0,85 | 1,06 | 0 | 0,36 | OVERALL | 1754 | | 2358 |
| rs1801260 | CLOCK | BREAST | 3 | 0,98 | 0,86 | 1,13 | 57 | 0,81 | OVERALL | 2176 | | 2869 |
| rs1902618 | RORA | BREAST | 2 | 0,96 | 0,86 | 1,08 | 0 | 0,49 | OVERALL | 2271 | | 2316 |
| rs1916645 | RORA | BREAST | 2 | 1,02 | 0,73 | 1,43 | 82 | 0,91 | OVERALL | 2271 | | 2316 |
| rs1997644 | CSNK1E | BREAST | 2 | 0,93 | 0,8 | 1,07 | 41 | 0,31 | OVERALL | 2271 | | 2316 |
| rs2011857 | RORA | BREAST | 2 | 1 | 0,82 | 1,21 | 69 | 0,99 | OVERALL | 2271 | | 2316 |
| rs2028122 | RORA | BREAST | 2 | 0,98 | 0,81 | 1,17 | 66 | 0,78 | OVERALL | 2271 | | 2316 |
| rs2030619 | RORA | BREAST | 2 | 0,99 | 0,84 | 1,15 | 32 | 0,85 | OVERALL | 2271 | | 2316 |
| rs2062094 | RORA | BREAST | 2 | 1,07 | 0,96 | 1,18 | 0 | 0,23 | OVERALL | 2271 | | 2316 |
| rs2071427 | NR1D1 | BREAST | 2 | 0,99 | 0,93 | 1,06 | 0 | 0,81 | OVERALL | 3828 | | 6900 |
| rs2071570 | NR1D1 | BREAST | 2 | 1,01 | 0,94 | 1,09 | 0 | 0,73 | OVERALL | 3828 | | 6900 |
| rs2075984 | CSNK1E | BREAST | 2 | 1,03 | 0,97 | 1,1 | 0 | 0,32 | OVERALL | 3828 | | 6900 |
| rs2118326 | RORA | BREAST | 2 | 0,97 | 0,86 | 1,08 | 0 | 0,55 | OVERALL | 2271 | | 2316 |
| rs2253820 | PER1 | BREAST | 3 | 0,89 | 0,82 | 0,96 | 0 | 0,004 | OVERALL | 4437 | | 8116 |
| rs2269457 | NR1D1 | BREAST | 2 | 0,98 | 0,91 | 1,05 | 0 | 0,53 | OVERALL | 3828 | | 6900 |
| rs2278637 | PER1 | BREAST | 2 | 0,93 | 0,83 | 1,04 | 0 | 0,22 | OVERALL | 1754 | | 2358 |
| rs2278749 | ARNTL | BREAST | 2 | 0,86 | 0,71 | 1,04 | 40 | 0,11 | OVERALL | 1708 | | 1761 |
| rs228642 | PER3 | BREAST | 3 | 0,98 | 0,88 | 1,09 | 58 | 0,71 | OVERALL | 3828 | | 6900 |
| rs228648 | PER3 | BREAST | 2 | 0,94 | 0,82 | 1,07 | 25 | 0,34 | OVERALL | 1754 | | 2358 |
| rs228651 | PER3 | BREAST | 2 | 0,96 | 0,9 | 1,03 | 7 | 0,27 | OVERALL | 1754 | | 2358 |
| rs228682 | PER3 | BREAST | 2 | 1 | 0,93 | 1,08 | 26 | 0,93 | OVERALL | 3828 | | 6900 |
| rs228729 | PER3 | BREAST | 3 | 1 | 0,95 | 1,07 | 0 | 0,88 | OVERALL | 4437 | | 8116 |
| rs2290037 | ARNTL | BREAST | 2 | 0,93 | 0,82 | 1,06 | 0 | 0,27 | OVERALL | 3828 | | 6900 |
| rs2291738 | TIMELESS | BREAST | 3 | 1 | 0,92 | 1,1 | 0 | 0,92 | OVERALL | 2712 | | 2795 |
| rs2292910 | CRY2 | BREAST | 2 | 1,03 | 0,92 | 1,14 | 17 | 0,64 | OVERALL | 1735 | | 2390 |
| rs2292912 | CRY2 | BREAST | 2 | 0,97 | 0,73 | 1,28 | 78 | 0,82 | OVERALL | 1567 | | 1653 |
| rs2304673 | PER2 | BREAST | 2 | 0,98 | 0,9 | 1,07 | 0 | 0,67 | OVERALL | 3828 | | 6900 |
| rs2304674 | PER2 | BREAST | 3 | 1,02 | 0,96 | 1,08 | 0 | 0,55 | OVERALL | 4437 | | 8116 |
| rs2305159 | NPAS2 | BREAST | 2 | 0,95 | 0,85 | 1,07 | 50 | 0,4 | OVERALL | 3311 | | 6942 |
| rs2305160 | NPAS2 | BREAST | 7 | 0,95 | 0,9 | 1,01 | 24 | 0,1 | OVERALL | 6519 | | 10415 |
| rs2306502 | RORA | BREAST | 2 | 0,98 | 0,83 | 1,15 | 0 | 0,78 | OVERALL | 2271 | | 2316 |
| rs2314339 | NR1D1 | BREAST | 2 | 0,99 | 0,87 | 1,13 | 0 | 0,88 | OVERALL | 2271 | | 2316 |
| rs2414686 | RORA | BREAST | 2 | 0,99 | 0,89 | 1,09 | 0 | 0,84 | OVERALL | 2271 | | 2316 |
| rs2414687 | RORA | BREAST | 2 | 1,04 | 0,93 | 1,15 | 0 | 0,49 | OVERALL | 2271 | | 2316 |
| rs2689352 | RORA | BREAST | 2 | 1,02 | 0,8 | 1,28 | 79 | 0,9 | OVERALL | 2271 | | 2316 |
| rs2899664 | RORA | BREAST | 2 | 0,98 | 0,89 | 1,08 | 0 | 0,72 | OVERALL | 2271 | | 2316 |
| rs2899666 | RORA | BREAST | 2 | 1,13 | 1 | 1,28 | 26 | 0,04 | OVERALL | 2271 | | 2316 |
| rs3027188 | PER1 | BREAST | 2 | 0,92 | 0,82 | 1,02 | 13 | 0,11 | OVERALL | 2688 | | 3364 |
| rs3027279 | PER1 | BREAST | 2 | 1,04 | 0,93 | 1,16 | 0 | 0,48 | OVERALL | 1754 | | 2358 |
| rs339972 | RORA | BREAST | 2 | 1,07 | 0,95 | 1,21 | 16 | 0,25 | OVERALL | 2271 | | 2316 |
| rs339996 | RORA | BREAST | 2 | 1,03 | 0,93 | 1,14 | 0 | 0,56 | OVERALL | 2271 | | 2316 |
| rs339998 | RORA | BREAST | 2 | 1,02 | 0,92 | 1,13 | 3 | 0,76 | OVERALL | 2271 | | 2316 |
| rs340023 | RORA | BREAST | 2 | 0,98 | 0,88 | 1,09 | 0 | 0,7 | OVERALL | 2271 | | 2316 |
| rs340026 | RORA | BREAST | 2 | 1,11 | 0,98 | 1,25 | 0 | 0,1 | OVERALL | 2271 | | 2316 |
| rs341365 | RORA | BREAST | 2 | 0,99 | 0,83 | 1,17 | 60 | 0,91 | OVERALL | 2271 | | 2316 |
| rs341366 | RORA | BREAST | 2 | 0,98 | 0,89 | 1,08 | 0 | 0,75 | OVERALL | 2271 | | 2316 |
| rs341382 | RORA | BREAST | 2 | 1 | 0,9 | 1,11 | 0 | 0,94 | OVERALL | 2271 | | 2316 |
| rs341392 | RORA | BREAST | 2 | 1,01 | 0,91 | 1,13 | 0 | 0,84 | OVERALL | 2271 | | 2316 |
| rs341399 | RORA | BREAST | 2 | 0,9 | 0,79 | 1,02 | 0 | 0,09 | OVERALL | 2271 | | 2316 |
| rs341403 | RORA | BREAST | 2 | 1,06 | 0,94 | 1,19 | 15 | 0,34 | OVERALL | 2271 | | 2316 |
| rs341459 | RORA | BREAST | 2 | 0,91 | 0,75 | 1,1 | 69 | 0,32 | OVERALL | 2271 | | 2316 |
| rs356642 | NPAS2 | BREAST | 2 | 0,79 | 0,61 | 1,01 | 82 | 0,06 | OVERALL | 3347 | | 6532 |
| rs356643 | NPAS2 | BREAST | 2 | 1,01 | 0,9 | 1,13 | 0 | 0,86 | OVERALL | 1754 | | 2358 |
| rs356652 | NPAS2 | BREAST | 2 | 1,06 | 0,85 | 1,33 | 70 | 0,6 | OVERALL | 3828 | | 6900 |
| rs3739008 | NPAS2 | BREAST | 4 | 1,04 | 0,96 | 1,12 | 25 | 0,38 | OVERALL | 2271 | | 2316 |
| rs3743266 | RORA | BREAST | 2 | 0,95 | 0,85 | 1,07 | 23 | 0,44 | OVERALL | 2271 | | 2316 |
| rs3744805 | NR1D1 | BREAST | 2 | 0,97 | 0,89 | 1,06 | 0 | 0,53 | OVERALL | 3828 | | 6900 |
| rs3749474 | CLOCK | BREAST | 2 | 0,86 | 0,76 | 0,98 | 0 | 0,02 | OVERALL | 1004 | | 1098 |
| rs3750420 | RORB | BREAST | 3 | 1,03 | 0,94 | 1,12 | 0 | 0,54 | OVERALL | 2834 | | 2935 |
| rs3754674 | NPAS2 | BREAST | 2 | 0,96 | 0,88 | 1,05 | 0 | 0,4 | OVERALL | 1735 | | 2390 |
| rs3754675 | NPAS2 | BREAST | 2 | 1,16 | 0,99 | 1,36 | 0 | 0,08 | OVERALL | 2271 | | 2316 |
| rs3768984 | *NPAS2* | BREAST | 2 | 0,99 | 0,92 | 1,06 | 0 | 0,77 | OVERALL | 3311 | | 6942 |
| rs3768991 | NPAS2 | BREAST | 2 | 1,02 | 0,92 | 1,12 | 0 | 0,7 | OVERALL | 2271 | | 2316 |
| rs3789327 | ARNTL | BREAST | 3 | 0,97 | 0,92 | 1,03 | 6 | 0,36 | OVERALL | 4437 | | 8116 |
| rs3792603 | CLOCK | BREAST | 2 | 0,97 | 0,87 | 1,08 | 0 | 0,54 | OVERALL | 2271 | | 2316 |
| rs3816358 | ARNTL | BREAST | 3 | 0,89 | 0,75 | 1,07 | 59 | 0,22 | OVERALL | 3293 | | 3330 |
| rs3816360 | ARNTL | BREAST | 3 | 1,09 | 0,94 | 1,26 | 64 | 0,27 | OVERALL | 3655 | | 6700 |
| rs3820787 | NPAS2 | BREAST | 2 | 0,88 | 0,8 | 0,96 | 0 | 0,005 | OVERALL | 1735 | | 2390 |
| rs3824872 | CRY2 | BREAST | 2 | 1,02 | 0,94 | 1,1 | 0 | 0,69 | OVERALL | 3311 | | 6942 |
| rs3849381 | NPAS2 | BREAST | 3 | 1,02 | 0,96 | 1,08 | 0 | 0,51 | OVERALL | 4437 | | 8116 |
| rs3888170 | NPAS2 | BREAST | 2 | 1,09 | 0,97 | 1,24 | 0 | 0,16 | OVERALL | 1754 | | 2358 |
| rs3905275 | RORA | BREAST | 2 | 0,96 | 0,8 | 1,14 | 55 | 0,64 | OVERALL | 2271 | | 2316 |
| rs4090240 | RORB | BREAST | 2 | 1,06 | 0,96 | 1,18 | 0 | 0,26 | OVERALL | 2271 | | 2316 |
| rs4238351 | RORA | BREAST | 2 | 1,03 | 0,93 | 1,13 | 0 | 0,61 | OVERALL | 2271 | | 2316 |
| rs4594196 | RORA | BREAST | 2 | 1,04 | 0,93 | 1,18 | 0 | 0,46 | OVERALL | 2271 | | 2316 |
| rs4757144 | ARNTL | BREAST | 3 | 1,01 | 0,95 | 1,08 | 16 | 0,67 | OVERALL | 4437 | | 8116 |
| rs4774371 | RORA | BREAST | 2 | 1 | 0,89 | 1,12 | 0 | 0,99 | OVERALL | 2271 | | 2316 |
| rs4774381 | RORA | BREAST | 2 | 1,01 | 0,9 | 1,13 | 0 | 0,87 | OVERALL | 2271 | | 2316 |
| rs4774386 | RORA | BREAST | 2 | 0,93 | 0,83 | 1,06 | 0 | 0,27 | OVERALL | 2271 | | 2316 |
| rs4774388 | RORA | BREAST | 2 | 0,9 | 0,8 | 1,02 | 0 | 0,09 | OVERALL | 2271 | | 2316 |
| rs4775292 | RORA | BREAST | 2 | 1,07 | 0,88 | 1,28 | 66 | 0,5 | OVERALL | 2271 | | 2316 |
| rs4775340 | RORA | BREAST | 2 | 0,99 | 0,88 | 1,12 | 30 | 0,91 | OVERALL | 2271 | | 2316 |
| rs4775351 | RORA | BREAST | 2 | 1,01 | 0,89 | 1,14 | 0 | 0,89 | OVERALL | 2271 | | 2316 |
| rs4775352 | RORA | BREAST | 2 | 1 | 0,85 | 1,19 | 31 | 0,96 | OVERALL | 2271 | | 2316 |
| rs4775355 | RORA | BREAST | 2 | 0,89 | 0,79 | 0,99 | 0 | 0,03 | OVERALL | 2271 | | 2316 |
| rs4775362 | RORA | BREAST | 2 | 1,06 | 0,95 | 1,18 | 0 | 0,27 | OVERALL | 2271 | | 2316 |
| rs4775369 | RORA | BREAST | 2 | 0,97 | 0,88 | 1,07 | 0 | 0,56 | OVERALL | 2271 | | 2316 |
| rs4775371 | RORA | BREAST | 2 | 0,97 | 0,86 | 1,08 | 0 | 0,54 | OVERALL | 2271 | | 2316 |
| rs4794826 | NR1D1 | BREAST | 2 | 1,03 | 0,88 | 1,22 | 19 | 0,69 | OVERALL | 2271 | | 2316 |
| rs4851377 | *NPAS2* | BREAST | 3 | 1 | 0,92 | 1,08 | 0 | 0,98 | OVERALL | 2880 | | 3532 |
| rs4851384 | NPAS2 | BREAST | 2 | 0,92 | 0,82 | 1,03 | 0 | 0,13 | OVERALL | 3828 | | 6900 |
| rs4851390 | NPAS2 | BREAST | 3 | 0,96 | 0,9 | 1,03 | 0 | 0,3 | OVERALL | 4437 | | 8116 |
| rs4864540 | CLOCK | BREAST | 2 | 0,99 | 0,88 | 1,11 | 0 | 0,81 | OVERALL | 1754 | | 2358 |
| rs5757037 | CSNK1E | BREAST | 2 | 0,97 | 0,81 | 1,15 | 48 | 0,72 | OVERALL | 1708 | | 1761 |
| rs6001093 | CSNK1E | BREAST | 2 | 1,01 | 0,93 | 1,1 | 0 | 0,78 | OVERALL | 3828 | | 6900 |
| rs6486121 | ARNTL | BREAST | 3 | 1,01 | 0,95 | 1,07 | 0 | 0,8 | OVERALL | 4437 | | 8116 |
| rs6494217 | RORA | BREAST | 2 | 0,99 | 0,88 | 1,11 | 8 | 0,82 | OVERALL | 2271 | | 2316 |
| rs6494219 | RORA | BREAST | 2 | 0,95 | 0,86 | 1,06 | 0 | 0,36 | OVERALL | 2271 | | 2316 |
| rs6494221 | RORA | BREAST | 2 | 0,99 | 0,88 | 1,12 | 18 | 0,88 | OVERALL | 2271 | | 2316 |
| rs6494229 | RORA | BREAST | 2 | 1,02 | 0,86 | 1,2 | 56 | 0,84 | OVERALL | 2271 | | 2316 |
| rs6494232 | RORA | BREAST | 2 | 0,93 | 0,74 | 1,17 | 62 | 0,53 | OVERALL | 2271 | | 2316 |
| rs6719533 | NPAS2 | BREAST | 2 | 1,02 | 0,93 | 1,11 | 0 | 0,73 | OVERALL | 1735 | | 2390 |
| rs6725296 | NPAS2 | BREAST | 2 | 1 | 0,89 | 1,12 | 0 | 0,97 | OVERALL | 1735 | | 2390 |
| rs6740935 | NPAS2 | BREAST | 2 | 1 | 0,9 | 1,12 | 53 | 0,95 | OVERALL | 3311 | | 6942 |
| rs6747755 | NPAS2 | BREAST | 2 | 0,96 | 0,87 | 1,07 | 0 | 0,5 | OVERALL | 1735 | | 2390 |
| rs6850524 | CLOCK | BREAST | 2 | 0,95 | 0,84 | 1,07 | 0 | 0,39 | OVERALL | 1086 | | 1285 |
| rs697673 | PER3 | BREAST | 2 | 1,06 | 0,83 | 1,35 | 75 | 0,62 | OVERALL | 1754 | | 2358 |
| rs697690 | PER3 | BREAST | 2 | 0,98 | 0,82 | 1,16 | 68 | 0,78 | OVERALL | 1735 | | 2390 |
| rs7022435 | RORB | BREAST | 2 | 1 | 0,9 | 1,11 | 0 | 0,98 | OVERALL | 1689 | | 1793 |
| rs7032677 | RORB | BREAST | 2 | 1,04 | 0,94 | 1,14 | 0 | 0,48 | OVERALL | 2271 | | 2316 |
| rs7037043 | RORB | BREAST | 2 | 0,97 | 0,88 | 1,08 | 0 | 0,58 | OVERALL | 2271 | | 2316 |
| rs707455 | PER3 | BREAST | 2 | 0,97 | 0,77 | 1,22 | 74 | 0,78 | OVERALL | 1754 | | 2358 |
| rs707467 | PER3 | BREAST | 2 | 0,95 | 0,84 | 1,07 | 0 | 0,4 | OVERALL | 2271 | | 2316 |
| rs7123390 | CRY2 | BREAST | 2 | 0,91 | 0,81 | 1,02 | 0 | 0,09 | OVERALL | 1567 | | 1653 |
| rs7164773 | RORA | BREAST | 2 | 1,16 | 1,05 | 1,29 | 0 | 0,003 | OVERALL | 2271 | | 2316 |
| rs7166448 | RORA | BREAST | 2 | 0,9 | 0,77 | 1,04 | 0 | 0,14 | OVERALL | 2271 | | 2316 |
| rs7168782 | RORA | BREAST | 2 | 0,94 | 0,84 | 1,05 | 0 | 0,28 | OVERALL | 2271 | | 2316 |
| rs7172348 | RORA | BREAST | 2 | 0,92 | 0,81 | 1,05 | 29 | 0,21 | OVERALL | 2271 | | 2316 |
| rs7177611 | RORA | BREAST | 2 | 0,96 | 0,77 | 1,2 | 76 | 0,74 | OVERALL | 2271 | | 2316 |
| rs7183955 | RORA | BREAST | 2 | 0,98 | 0,8 | 1,2 | 66 | 0,84 | OVERALL | 2271 | | 2316 |
| rs726955 | RORA | BREAST | 2 | 0,94 | 0,85 | 1,05 | 0 | 0,28 | OVERALL | 2271 | | 2316 |
| rs7297614 | CRY1 | BREAST | 3 | 0,97 | 0,9 | 1,05 | 0 | 0,48 | OVERALL | 2271 | | 2316 |
| rs7302060 | TIMELESS | BREAST | 2 | 0,96 | 0,85 | 1,08 | 0 | 0,53 | OVERALL | 1086 | | 1285 |
| rs7581886 | NPAS2 | BREAST | 2 | 0,86 | 0,75 | 0,98 | 0 | 0,03 | OVERALL | 1735 | | 2390 |
| rs7584376 | PER2 | BREAST | 2 | 1,02 | 0,92 | 1,15 | 0 | 0,66 | OVERALL | 1754 | | 2358 |
| rs7598826 | NPAS2 | BREAST | 3 | 0,95 | 0,9 | 1,01 | 0 | 0,11 | OVERALL | 4437 | | 8116 |
| rs7602358 | PER2 | BREAST | 2 | 1,06 | 0,96 | 1,18 | 0 | 0,24 | OVERALL | 2271 | | 2316 |
| rs7605570 | NPAS2 | BREAST | 2 | 0,9 | 0,81 | 1,01 | 0 | 0,07 | OVERALL | 3311 | | 6942 |
| rs7698022 | CLOCK | BREAST | 3 | 1,01 | 0,85 | 1,19 | 66 | 0,93 | OVERALL | 2212 | | 2459 |
| rs782903 | RORA | BREAST | 2 | 0,98 | 0,88 | 1,08 | 0 | 0,65 | OVERALL | 2271 | | 2316 |
| rs782907 | RORA | BREAST | 2 | 0,95 | 0,85 | 1,06 | 8 | 0,33 | OVERALL | 2271 | | 2316 |
| rs782910 | RORA | BREAST | 2 | 0,99 | 0,89 | 1,11 | 0 | 0,91 | OVERALL | 2271 | | 2316 |
| rs782926 | RORA | BREAST | 2 | 0,96 | 0,87 | 1,06 | 0 | 0,42 | OVERALL | 2271 | | 2316 |
| rs782938 | RORA | BREAST | 2 | 0,96 | 0,87 | 1,07 | 0 | 0,49 | OVERALL | 2271 | | 2316 |
| rs782944 | RORA | BREAST | 2 | 0,92 | 0,7 | 1,21 | 83 | 0,57 | OVERALL | 2271 | | 2316 |
| rs782947 | RORA | BREAST | 2 | 1,01 | 0,88 | 1,17 | 33 | 0,85 | OVERALL | 2271 | | 2316 |
| rs7848922 | RORB | BREAST | 2 | 0,92 | 0,79 | 1,07 | 49 | 0,26 | OVERALL | 2271 | | 2316 |
| rs7867494 | RORB | BREAST | 2 | 0,9 | 0,81 | 1 | 0 | 0,04 | OVERALL | 2271 | | 2316 |
| rs7942486 | ARNTL | BREAST | 2 | 0,98 | 0,87 | 1,1 | 0 | 0,73 | OVERALL | 1754 | | 2358 |
| rs7950226 | ARNTL | BREAST | 3 | 1 | 0,92 | 1,08 | 0 | 0,99 | OVERALL | 2880 | | 3532 |
| rs8023252 | RORA | BREAST | 2 | 0,94 | 0,85 | 1,03 | 0 | 0,2 | OVERALL | 2271 | | 2316 |
| rs8024629 | RORA | BREAST | 2 | 0,89 | 0,8 | 1 | 0 | 0,05 | OVERALL | 2271 | | 2316 |
| rs8027032 | RORA | BREAST | 2 | 0,98 | 0,86 | 1,12 | 30 | 0,79 | OVERALL | 2271 | | 2316 |
| rs8027829 | RORA | BREAST | 2 | 0,99 | 0,89 | 1,1 | 0 | 0,83 | OVERALL | 2271 | | 2316 |
| rs8031801 | RORA | BREAST | 2 | 1,09 | 0,95 | 1,25 | 29 | 0,2 | OVERALL | 2271 | | 2316 |
| rs8032023 | RORA | BREAST | 2 | 0,97 | 0,88 | 1,08 | 0 | 0,6 | OVERALL | 2271 | | 2316 |
| rs8034880 | RORA | BREAST | 2 | 0,98 | 0,77 | 1,24 | 63 | 0,84 | OVERALL | 2271 | | 2316 |
| rs8034950 | RORA | BREAST | 2 | 0,95 | 0,86 | 1,05 | 0 | 0,31 | OVERALL | 2271 | | 2316 |
| rs8036866 | RORA | BREAST | 2 | 1,11 | 0,95 | 1,28 | 0 | 0,19 | OVERALL | 2271 | | 2316 |
| rs8037669 | RORA | BREAST | 2 | 1 | 0,9 | 1,1 | 0 | 0,94 | OVERALL | 2271 | | 2316 |
| rs8038077 | RORA | BREAST | 2 | 1,17 | 0,98 | 1,38 | 0 | 0,08 | OVERALL | 2271 | | 2316 |
| rs8041061 | RORA | BREAST | 2 | 0,95 | 0,86 | 1,04 | 0 | 0,27 | OVERALL | 2271 | | 2316 |
| rs8041381 | RORA | BREAST | 2 | 0,92 | 0,83 | 1,01 | 0 | 0,08 | OVERALL | 2271 | | 2316 |
| rs8042149 | RORA | BREAST | 2 | 0,96 | 0,87 | 1,05 | 0 | 0,37 | OVERALL | 2271 | | 2316 |
| rs8043356 | RORA | BREAST | 2 | 1,02 | 0,87 | 1,19 | 30 | 0,8 | OVERALL | 2271 | | 2316 |
| rs809736 | RORA | BREAST | 2 | 0,97 | 0,87 | 1,09 | 0 | 0,63 | OVERALL | 2271 | | 2316 |
| rs875994 | PER3 | BREAST | 2 | 1,08 | 1 | 1,17 | 0 | 0,06 | OVERALL | 3828 | | 6900 |
| rs880625 | RORA | BREAST | 2 | 0,98 | 0,87 | 1,11 | 0 | 0,78 | OVERALL | 2271 | | 2316 |
| rs880626 | RORA | BREAST | 2 | 0,97 | 0,88 | 1,08 | 0 | 0,63 | OVERALL | 2271 | | 2316 |
| rs895520 | NPAS2 | BREAST | 2 | 1,09 | 1,02 | 1,16 | 0 | 0,006 | OVERALL | 3828 | | 6900 |
| rs895521 | NPAS2 | BREAST | 2 | 0,96 | 0,9 | 1,04 | 0 | 0,33 | OVERALL | 3828 | | 6900 |
| rs919000 | RORA | BREAST | 2 | 1,02 | 0,91 | 1,14 | 0 | 0,79 | OVERALL | 2271 | | 2316 |
| rs934945 | PER2 | BREAST | 3 | 1 | 0,94 | 1,07 | 0 | 0,94 | OVERALL | 5366 | | 8505 |
| rs935401 | NPAS2 | BREAST | 2 | 1,11 | 1,02 | 1,22 | 0 | 0,02 | OVERALL | 1735 | | 2390 |
| rs940222 | RORA | BREAST | 2 | 0,98 | 0,88 | 1,09 | 0 | 0,72 | OVERALL | 1126 | | 1174 |
| rs940224 | RORA | BREAST | 2 | 1 | 0,9 | 1,11 | 0 | 0,98 | OVERALL | 1126 | | 1174 |
| rs965519 | NPAS2 | BREAST | 3 | 1,07 | 0,98 | 1,18 | 26 | 0,14 | OVERALL | 4437 | | 8116 |
| rs968357 | RORB | BREAST | 2 | 1,02 | 0,91 | 1,15 | 0 | 0,7 | OVERALL | 2271 | | 2316 |
| rs969485 | ARNTL | BREAST | 4 | 1,05 | 0,93 | 1,17 | 44 | 0,66 | OVERALL | 5000 | | 8735 |
| rs974828 | RORA | BREAST | 2 | 0,89 | 0,77 | 1,03 | 0 | 0,11 | OVERALL | 2271 | | 2316 |
| rs9788704 | RORA | BREAST | 2 | 1 | 0,83 | 1,21 | 68 | 0,99 | OVERALL | 2271 | | 2316 |
| rs9806453 | RORA | BREAST | 2 | 1,02 | 0,9 | 1,15 | 25 | 0,78 | OVERALL | 2271 | | 2316 |
| rs9920560 | RORA | BREAST | 2 | 1,13 | 0,99 | 1,29 | 2 | 0,07 | OVERALL | 2271 | | 2316 |
| rs1012477 | PER3 | BREAST | 3 | 0,91 | 0,81 | 1,04 | 0 | 0,16 | ANY SHIFTWORK |  |  | |
| rs10462023 | PER2 | BREAST | 3 | 0,99 | 0,89 | 1,09 | 0 | 0,84 | ANY SHIFTWORK |  |  | |
| rs10462028 | CLOCK | BREAST | 2 | 1,02 | 0,72 | 1,43 | 60 | 0,92 | ANY SHIFTWORK |  |  | |
| rs10838524 | CRY2 | BREAST | 2 | 1,06 | 0,94 | 1,19 | 0 | 0,33 | ANY SHIFTWORK |  |  | |
| rs11123857 | NPAS2 | BREAST | 3 | 0,95 | 0,74 | 1,21 | 75 | 0,66 | ANY SHIFTWORK |  |  | |
| rs11133373 | CLOCK | BREAST | 2 | 0,98 | 0,65 | 1,46 | 88 | 0,91 | ANY SHIFTWORK |  |  | |
| rs1401417 | CRY2 | BREAST | 3 | 0,93 | 0,84 | 1,03 | 0 | 0,18 | ANY SHIFTWORK |  |  | |
| rs17024926 | NPAS2 | BREAST | 2 | 0,96 | 0,85 | 1,07 | 0 | 0,44 | ANY SHIFTWORK |  |  | |
| rs3750420 | RORB | BREAST | 2 | 1,04 | 0,93 | 1,16 | 0 | 0,51 | ANY SHIFTWORK |  |  | |
| rs3816358 | ARNTL | BREAST | 2 | 0,95 | 0,78 | 1,15 | 0 | 0,59 | ANY SHIFTWORK |  |  | |
| rs7022435 | RORB | BREAST | 2 | 1,01 | 0,89 | 1,14 | 0 | 0,89 | ANY SHIFTWORK |  |  | |
| rs969485 | ARNTL | BREAST | 3 | 1,18 | 0,93 | 1,49 | 79 | 0,18 | ANY SHIFTWORK |  |  | |
| rs1012477 | PER3 | BREAST | 2 | 0,93 | 0,88 | 1,08 | 0 | 0,33 | <2YSHIFTWORK |  | | |
| rs10165970 | NPAS2 | BREAST | 2 | 1,19 | 1,03 | 1,37 | 0 | 0,02 | <2YSHIFTWORK |  | | |
| rs10462023 | PER2 | BREAST | 2 | 0,98 | 0,88 | 1,1 | 0 | 0,75 | <2YSHIFTWORK |  | | |
| rs10766074 | ARNTL | BREAST | 2 | 1,05 | 0,91 | 1,22 | 0 | 0,51 | <2YSHIFTWORK |  | | |
| rs11022778 | ARNTL | BREAST | 2 | 0,95 | 0,85 | 1,07 | 0 | 0,42 | <2YSHIFTWORK |  | | |
| rs11113179 | CRY1 | BREAST | 3 | 1,03 | 0,86 | 1,24 | 17 | 0,71 | <2YSHIFTWORK |  | | |
| rs11123857 | NPAS2 | BREAST | 2 | 1,07 | 0,94 | 1,21 | 0 | 0,32 | <2YSHIFTWORK |  | | |
| rs11894535 | PER2 | BREAST | 2 | 0,94 | 0,8 | 1,12 | 0 | 0,51 | <2YSHIFTWORK |  | | |
| rs12421530 | ARNTL | BREAST | 2 | 1,06 | 0,94 | 1,18 | 0 | 0,36 | <2YSHIFTWORK |  | | |
| rs12472321 | NPAS2 | BREAST | 2 | 0,95 | 0,78 | 1,16 | 51 | 0,63 | <2YSHIFTWORK |  | | |
| rs12712083 | NPAS2 | BREAST | 2 | 1,06 | 0,95 | 1,18 | 0 | 0,3 | <2YSHIFTWORK |  | | |
| rs12712085 | NPAS2 | BREAST | 2 | 0,79 | 0,63 | 1,01 | 70 | 0,06 | <2YSHIFTWORK |  | | |
| rs13025524 | NPAS2 | BREAST | 2 | 0,98 | 0,87 | 1,09 | 0 | 0,67 | <2YSHIFTWORK |  | | |
| rs13026599 | NPAS2 | BREAST | 2 | 1,05 | 0,89 | 1,24 | 21 | 0,57 | <2YSHIFTWORK |  | | |
| rs1369481 | NPAS2 | BREAST | 2 | 1,02 | 0,84 | 1,23 | 51 | 0,87 | <2YSHIFTWORK |  | | |
| rs1401417 | CRY2 | BREAST | 2 | 0,98 | 0,86 | 1,12 | 0 | 0,79 | <2YSHIFTWORK |  | | |
| rs1534891 | CSNK1E | BREAST | 2 | 0,92 | 0,72 | 1,17 | 38 | 0,47 | <2YSHIFTWORK |  | | |
| rs1562438 | ARNTL | BREAST | 2 | 1,04 | 0,92 | 1,18 | 0 | 0,49 | <2YSHIFTWORK |  | | |
| rs17020663 | NPAS2 | BREAST | 2 | 0,99 | 0,85 | 1,17 | 0 | 0,95 | <2YSHIFTWORK |  | | |
| rs17025005 | NPAS2 | BREAST | 2 | 0,94 | 0,82 | 1,08 | 0 | 0,36 | <2YSHIFTWORK |  | | |
| rs17452383 | ARNTL | BREAST | 2 | 0,94 | 0,8 | 1,11 | 0 | 0,48 | <2YSHIFTWORK |  | | |
| rs1801260 | CLOCK | BREAST | 2 | 0,92 | 0,82 | 1,04 | 0 | 0,17 | <2YSHIFTWORK |  | | |
| rs2253820 | PER1 | BREAST | 2 | 0,97 | 0,84 | 1,13 | 0 | 0,71 | <2YSHIFTWORK |  | | |
| rs228729 | PER3 | BREAST | 2 | 1,02 | 0,9 | 1,14 | 0 | 0,8 | <2YSHIFTWORK |  | | |
| rs2292910 | CRY2 | BREAST | 2 | 1,03 | 0,92 | 1,15 | 0 | 0,65 | <2YSHIFTWORK |  | | |
| rs2304674 | PER2 | BREAST | 2 | 1,06 | 0,94 | 1,19 | 0 | 0,38 | <2YSHIFTWORK |  | | |
| rs2305160 | NPAS2 | BREAST | 2 | 0,77 | 0,58 | 1,02 | 77 | 0,06 | <2YSHIFTWORK |  | | |
| rs3027188 | PER1 | BREAST | 3 | 0,97 | 0,85 | 1,1 | 0 | 0,61 | <2YSHIFTWORK |  | | |
| rs3754674 | NPAS2 | BREAST | 2 | 0,95 | 0,85 | 1,06 | 0 | 0,38 | <2YSHIFTWORK |  | | |
| rs3789327 | ARNTL | BREAST | 2 | 0,98 | 0,88 | 1,1 | 0 | 0,75 | <2YSHIFTWORK |  | | |
| rs3820787 | NPAS2 | BREAST | 2 | 0,79 | 0,63 | 0,99 | 63 | 0,04 | <2YSHIFTWORK |  | | |
| rs3849381 | NPAS2 | BREAST | 2 | 0,98 | 0,87 | 1,1 | 0 | 0,69 | <2YSHIFTWORK |  | | |
| rs4757144 | ARNTL | BREAST | 2 | 0,97 | 0,85 | 1,11 | 24 | 0,7 | <2YSHIFTWORK |  | | |
| rs4851377 | *NPAS2* | BREAST | 2 | 1,06 | 0,95 | 1,18 | 0 | 0,3 | <2YSHIFTWORK |  | | |
| rs4851390 | NPAS2 | BREAST | 2 | 1 | 0,87 | 1,15 | 0 | 0,99 | <2YSHIFTWORK |  | | |
| rs6486121 | ARNTL | BREAST | 2 | 1,08 | 0,96 | 1,21 | 2 | 0,21 | <2YSHIFTWORK |  | | |
| rs6719533 | NPAS2 | BREAST | 2 | 1,06 | 0,95 | 1,18 | 0 | 0,3 | <2YSHIFTWORK |  | | |
| rs6725296 | NPAS2 | BREAST | 2 | 1,06 | 0,93 | 1,22 | 0 | 0,37 | <2YSHIFTWORK |  | | |
| rs6747755 | NPAS2 | BREAST | 2 | 1 | 0,86 | 1,16 | 13 | 0,99 | <2YSHIFTWORK |  | | |
| rs697690 | PER3 | BREAST | 2 | 1 | 0,77 | 1,31 | 75 | 0,98 | <2YSHIFTWORK |  | | |
| rs7581886 | NPAS2 | BREAST | 2 | 0,78 | 0,6 | 1,01 | 49 | 0,06 | <2YSHIFTWORK |  | | |
| rs7598826 | NPAS2 | BREAST | 2 | 0,98 | 0,88 | 1,1 | 0 | 0,77 | <2YSHIFTWORK |  | | |
| rs7950226 | ARNTL | BREAST | 2 | 0,95 | 0,85 | 1,06 | 0 | 0,36 | <2YSHIFTWORK |  | | |
| rs935401 | NPAS2 | BREAST | 2 | 1,08 | 0,96 | 1,2 | 0 | 0,19 | <2YSHIFTWORK |  | | |
| rs965519 | NPAS2 | BREAST | 2 | 1,06 | 0,92 | 1,22 | 0 | 0,44 | <2YSHIFTWORK |  | | |
| rs969485 | ARNTL | BREAST | 2 | 1,05 | 0,88 | 1,27 | 47 | 0,57 | <2YSHIFTWORK |  | | |
| rs1012477 | PER3 | BREAST | 2 | 0,86 | 0,63 | 1,17 | 0 | 0,33 | >2YSHIFTWORK |  |  | |
| rs10165970 | NPAS2 | BREAST | 2 | 0,89 | 0,62 | 1,27 | 23 | 0,51 | >2YSHIFTWORK |  |  | |
| rs10462023 | PER2 | BREAST | 2 | 1,1 | 0,88 | 1,38 | 0 | 0,41 | >2YSHIFTWORK |  |  | |
| rs10766074 | ARNTL | BREAST | 2 | 0,91 | 0,68 | 1,21 | 0 | 0,51 | >2YSHIFTWORK |  |  | |
| rs11022778 | ARNTL | BREAST | 2 | 1,26 | 0,87 | 1,82 | 58 | 0,23 | >2YSHIFTWORK |  |  | |
| rs11113179 | CRY1 | BREAST | 3 | 0,93 | 0,57 | 1,51 | 63 | 0,77 | >2YSHIFTWORK |  |  | |
| rs11123857 | NPAS2 | BREAST | 2 | 1,26 | 0,68 | 2,33 | 81 | 0,47 | >2YSHIFTWORK |  |  | |
| rs11894535 | PER2 | BREAST | 2 | 1,11 | 0,68 | 1,8 | 79 | 0,68 | >2YSHIFTWORK |  |  | |
| rs12421530 | ARNTL | BREAST | 2 | 0,84 | 0,66 | 1,06 | 0 | 0,14 | >2YSHIFTWORK |  |  | |
| rs12472321 | NPAS2 | BREAST | 2 | 1 | 0,79 | 1,27 | 0 | 0,99 | >2YSHIFTWORK |  |  | |
| rs12712083 | NPAS2 | BREAST | 2 | 0,93 | 0,75 | 1,16 | 0 | 0,53 | >2YSHIFTWORK |  |  | |
| rs12712085 | NPAS2 | BREAST | 2 | 0,99 | 0,8 | 1,22 | 0 | 0,9 | >2YSHIFTWORK |  |  | |
| rs13025524 | NPAS2 | BREAST | 2 | 0,94 | 0,75 | 1,19 | 0 | 0,62 | >2YSHIFTWORK |  |  | |
| rs13026599 | NPAS2 | BREAST | 2 | 0,95 | 0,72 | 1,25 | 0 | 0,72 | >2YSHIFTWORK |  |  | |
| rs1369481 | NPAS2 | BREAST | 2 | 0,88 | 0,69 | 1,12 | 0 | 0,3 | >2YSHIFTWORK |  |  | |
| rs1401417 | CRY2 | BREAST | 2 | 0,71 | 0,54 | 0,93 | 0 | 0,01 | >2YSHIFTWORK |  |  | |
| rs1534891 | CSNK1E | BREAST | 2 | 0,77 | 0,57 | 1,04 | 0 | 0,09 | >2YSHIFTWORK |  |  | |
| rs1562438 | ARNTL | BREAST | 2 | 1,03 | 0,78 | 1,34 | 13 | 0,85 | >2YSHIFTWORK |  |  | |
| rs17020663 | NPAS2 | BREAST | 2 | 1 | 0,73 | 1,36 | 0 | 1 | >2YSHIFTWORK |  |  | |
| rs17025005 | NPAS2 | BREAST | 2 | 0,92 | 0,7 | 1,2 | 0 | 0,54 | >2YSHIFTWORK |  |  | |
| rs17452383 | ARNTL | BREAST | 2 | 1,13 | 0,83 | 1,55 | 0 | 0,43 | >2YSHIFTWORK |  |  | |
| rs1801260 | CLOCK | BREAST | 2 | 0,87 | 0,69 | 1,09 | 0 | 0,23 | >2YSHIFTWORK |  |  | |
| rs2253820 | PER1 | BREAST | 2 | 0,87 | 0,58 | 1,31 | 47 | 0,5 | >2YSHIFTWORK |  |  | |
| rs228729 | PER3 | BREAST | 2 | 1,05 | 0,83 | 1,32 | 3 | 0,68 | >2YSHIFTWORK |  |  | |
| rs2292910 | CRY2 | BREAST | 2 | 0,87 | 0,69 | 1,09 | 0 | 0,23 | >2YSHIFTWORK |  |  | |
| rs2304674 | PER2 | BREAST | 2 | 0,91 | 0,72 | 1,15 | 0 | 0,42 | >2YSHIFTWORK |  |  | |
| rs2305160 | NPAS2 | BREAST | 2 | 1,12 | 0,9 | 1,39 | 0 | 0,31 | >2YSHIFTWORK |  |  | |
| rs3027188 | PER1 | BREAST | 3 | 0,8 | 0,58 | 1,12 | 50 | 0,19 | >2YSHIFTWORK |  |  | |
| rs3754674 | NPAS2 | BREAST | 2 | 1,06 | 0,79 | 1,42 | 38 | 0,71 | >2YSHIFTWORK |  |  | |
| rs3789327 | ARNTL | BREAST | 2 | 1,24 | 0,6 | 2,59 | 89 | 0,56 | >2YSHIFTWORK |  |  | |
| rs3820787 | NPAS2 | BREAST | 2 | 0,98 | 0,79 | 1,22 | 0 | 0,86 | >2YSHIFTWORK |  |  | |
| rs3849381 | NPAS2 | BREAST | 2 | 1,02 | 0,81 | 1,3 | 0 | 0,84 | >2YSHIFTWORK |  |  | |
| rs4757144 | ARNTL | BREAST | 2 | 0,98 | 0,79 | 1,21 | 0 | 0,85 | >2YSHIFTWORK |  |  | |
| rs4851377 | *NPAS2* | BREAST | 2 | 0,92 | 0,73 | 1,14 | 0 | 0,43 | >2YSHIFTWORK |  |  | |
| rs4851390 | NPAS2 | BREAST | 2 | 1,26 | 0,8 | 1,99 | 53 | 0,32 | >2YSHIFTWORK |  |  | |
| rs6486121 | ARNTL | BREAST | 2 | 0,85 | 0,67 | 1,08 | 0 | 0,18 | >2YSHIFTWORK |  |  | |
| rs6719533 | NPAS2 | BREAST | 2 | 0,85 | 0,69 | 1,05 | 0 | 0,13 | >2YSHIFTWORK |  |  | |
| rs6725296 | NPAS2 | BREAST | 2 | 0,85 | 0,64 | 1,13 | 0 | 0,27 | >2YSHIFTWORK |  |  | |
| rs6747755 | NPAS2 | BREAST | 2 | 0,91 | 0,65 | 1,28 | 35 | 0,58 | >2YSHIFTWORK |  |  | |
| rs697690 | PER3 | BREAST | 2 | 0,96 | 0,76 | 1,22 | 9 | 0,77 | >2YSHIFTWORK |  |  | |
| rs7581886 | NPAS2 | BREAST | 2 | 1,11 | 0,8 | 1,55 | 0 | 0,53 | >2YSHIFTWORK |  |  | |
| rs7598826 | NPAS2 | BREAST | 2 | 0,91 | 0,72 | 1,15 | 8 | 0,41 | >2YSHIFTWORK |  |  | |
| rs7950226 | ARNTL | BREAST | 2 | 1,24 | 0,88 | 1,74 | 55 | 0,22 | >2YSHIFTWORK |  |  | |
| rs935401 | NPAS2 | BREAST | 2 | 1,23 | 0,87 | 1,74 | 56 | 0,24 | >2YSHIFTWORK |  |  | |
| rs965519 | NPAS2 | BREAST | 2 | 0,91 | 0,67 | 1,23 | 3 | 0,53 | >2YSHIFTWORK |  |  | |
| rs969485 | ARNTL | BREAST | 2 | 1,1 | 0,88 | 1,39 | 0 | 0,4 | >2YSHIFTWORK |  |  | |
| rs1012477 | PER3 | BREAST | 2 | 0,86 | 0,75 | 0,99 | 0 | 0,04 | ER/PR + |  | |  |
| rs11113179 | CRY1 | BREAST | 2 | 1,13 | 0,88 | 1,46 | 56 | 0,32 | ER/PR + |  | |  |
| rs11133391 | CLOCK | BREAST | 2 | 1 | 0,88 | 1,14 | 0 | 0,97 | ER/PR + |  | |  |
| rs11932595 | CLOCK | BREAST | 3 | 0,99 | 0,8 | 1,21 | 72 | 0,9 | ER/PR + |  | |  |
| rs1401417 | CRY2 | BREAST | 4 | 1,01 | 0,92 | 1,11 | 0 | 0,87 | ER/PR + |  | |  |
| rs1801260 | CLOCK | BREAST | 2 | 0,98 | 0,75 | 1,3 | 55 | 0,9 | ER/PR + |  | |  |
| rs2291738 | TIMELESS | BREAST | 2 | 0,99 | 0,89 | 1,11 | 0 | 0,89 | ER/PR + |  | |  |
| rs2292912 | CRY2 | BREAST | 2 | 0,95 | 0,76 | 1,18 | 24 | 0,63 | ER/PR + |  | |  |
| rs2305160 | NPAS2 | BREAST | 2 | 0,97 | 0,76 | 1,26 | 82 | 0,84 | ER/PR + |  | |  |
| rs3027188 | PER1 | BREAST | 2 | 0,86 | 0,71 | 1,03 | 43 | 0,11 | ER/PR + |  | |  |
| rs6850524 | CLOCK | BREAST | 2 | 0,94 | 0,81 | 1,09 | 0 | 0,43 | ER/PR + |  | |  |
| rs7123390 | CRY2 | BREAST | 2 | 0,93 | 0,82 | 1,06 | 0 | 0,3 | ER/PR + |  | |  |
| rs7302060 | TIMELESS | BREAST | 2 | 0,94 | 0,81 | 1,09 | 16 | 0,42 | ER/PR + |  | |  |
| rs7698022 | CLOCK | BREAST | 2 | 0,94 | 0,83 | 1,06 | 18 | 0,32 | ER/PR + |  | |  |
| rs934945 | PER2 | BREAST | 2 | 0,95 | 0,86 | 1,06 | 0 | 0,36 | ER/PR + |  | |  |
| rs1012477 | PER3 | BREAST | 2 | 1,11 | 0,87 | 1,42 | 0 | 0,38 | ER/PR - |  | |  |
| rs11113179 | CRY1 | BREAST | 2 | 1,14 | 0,8 | 1,62 | 26 | 0,46 | ER/PR - |  | |  |
| rs11133391 | CLOCK | BREAST | 2 | 1,04 | 0,85 | 1,26 | 0 | 0,73 | ER/PR - |  | |  |
| rs11932595 | CLOCK | BREAST | 3 | 1,16 | 0,91 | 1,49 | 56 | 0,23 | ER/PR - |  | |  |
| rs1401417 | CRY2 | BREAST | 4 | 1,24 | 0,99 | 1,56 | 52 | 0,06 | ER/PR - |  | |  |
| rs1801260 | CLOCK | BREAST | 2 | 1,16 | 0,86 | 1,55 | 46 | 0,33 | ER/PR - |  | |  |
| rs2291738 | TIMELESS | BREAST | 2 | 1,14 | 0,94 | 1,38 | 0 | 0,19 | ER/PR - |  | |  |
| rs2292912 | CRY2 | BREAST | 2 | 0,79 | 0,49 | 1,26 | 64 | 0,32 | ER/PR - |  | |  |
| rs2305160 | NPAS2 | BREAST | 2 | 0,95 | 0,74 | 1,21 | 36 | 0,68 | ER/PR - |  | |  |
| rs3027188 | PER1 | BREAST | 2 | 0,95 | 0,68 | 1,32 | 39 | 0,76 | ER/PR - |  | |  |
| rs6850524 | CLOCK | BREAST | 2 | 0,8 | 0,64 | 1,01 | 0 | 0,06 | ER/PR - |  | |  |
| rs7123390 | CRY2 | BREAST | 2 | 0,72 | 0,43 | 1,21 | 69 | 0,22 | ER/PR - |  | |  |
| rs7302060 | TIMELESS | BREAST | 2 | 0,85 | 0,68 | 1,07 | 0 | 0,17 | ER/PR - |  | |  |
| rs7698022 | CLOCK | BREAST | 2 | 1,11 | 0,92 | 1,35 | 12 | 0,28 | ER/PR - |  | |  |
| rs934945 | PER2 | BREAST | 2 | 1,11 | 0,98 | 1,27 | 0 | 0,1 | ER/PR - |  | |  |
| 4/5 repeats | PER3 | BREAST | 3 | 0,62 | 0,13 | 3,04 | 98 | 0,56 | PREMENOPAUSAL |  | | |
| rs1012477 | PER3 | BREAST | 2 | 0,73 | 0,59 | 0,91 | 0 | 0,005 | PREMENOPAUSAL |  | | |
| rs11113179 | CRY1 | BREAST | 2 | 1,03 | 0,72 | 1,48 | 48 | 0,86 | PREMENOPAUSAL |  | | |
| rs11932595 | CLOCK | BREAST | 2 | 1,15 | 0,98 | 1,33 | 0 | 0,08 | PREMENOPAUSAL |  | | |
| rs1401417 | CRY2 | BREAST | 4 | 1,61 | 0,45 | 5,74 | 99 | 0,46 | PREMENOPAUSAL |  | | |
| rs2292912 | CRY2 | BREAST | 2 | 0,92 | 0,75 | 1,13 | 0 | 0,43 | PREMENOPAUSAL |  | | |
| rs2305160 | NPAS2 | BREAST | 3 | 0,95 | 0,71 | 1,29 | 71 | 0,76 | PREMENOPAUSAL |  | | |
| rs3027188 | PER1 | BREAST | 2 | 0,86 | 0,69 | 1,06 | 0 | 0,15 | PREMENOPAUSAL |  | | |
| rs7123390 | CRY2 | BREAST | 2 | 0,95 | 0,66 | 1,36 | 61 | 0,76 | PREMENOPAUSAL |  | | |
| rs7698022 | CLOCK | BREAST | 2 | 1,07 | 0,88 | 1,31 | 20 | 0,47 | PREMENOPAUSAL |  | | |
| rs934945 | PER2 | BREAST | 2 | 0,48 | 0,14 | 1,64 | 98 | 0,24 | PREMENOPAUSAL |  | | |
| 4/5 repeats | PER3 | BREAST | 3 | 0,98 | 0,83 | 1,16 | 0 | 0,85 | POSTMENOPAUSAL |  |  | |
| rs1012477 | PER3 | BREAST | 2 | 1 | 0,85 | 1,17 | 0 | 1 | POSTMENOPAUSAL |  |  | |
| rs10162630 | RORA | BREAST | 2 | 1,04 | 0,93 | 1,17 | 0 | 0,48 | POSTMENOPAUSAL |  |  | |
| rs10165970 | NPAS2 | BREAST | 3 | 1,03 | 0,79 | 1,34 | 83 | 0,84 | POSTMENOPAUSAL |  | |  |
| rs10462018 | PER3 | BREAST | 2 | 1,08 | 0,93 | 1,24 | 0 | 0,3 | POSTMENOPAUSAL |  | |  |
| rs10462020 | PER3 | BREAST | 2 | 1 | 0,87 | 1,14 | 12 | 0,98 | POSTMENOPAUSAL |  | |  |
| rs10462021 | PER3 | BREAST | 2 | 0,99 | 0,86 | 1,14 | 18 | 0,86 | POSTMENOPAUSAL |  | |  |
| rs10462023 | PER2 | BREAST | 2 | 1,05 | 0,93 | 1,18 | 0 | 0,44 | POSTMENOPAUSAL |  | |  |
| rs10519070 | RORA | BREAST | 2 | 0,96 | 0,83 | 1,11 | 0 | 0,55 | POSTMENOPAUSAL |  | |  |
| rs10519097 | RORA | BREAST | 2 | 0,87 | 0,75 | 1 | 0 | 0,04 | POSTMENOPAUSAL |  | |  |
| rs10519099 | RORA | BREAST | 2 | 0,91 | 0,81 | 1,02 | 0 | 0,12 | POSTMENOPAUSAL |  | |  |
| rs10519111 | RORA | BREAST | 2 | 1,06 | 0,92 | 1,21 | 0 | 0,44 | POSTMENOPAUSAL |  | |  |
| rs10781235 | RORB | BREAST | 2 | 0,94 | 0,83 | 1,07 | 0 | 0,36 | POSTMENOPAUSAL |  | |  |
| rs10869410 | RORB | BREAST | 2 | 0,95 | 0,82 | 1,09 | 30 | 0,45 | POSTMENOPAUSAL |  | |  |
| rs10869417 | RORB | BREAST | 2 | 0,88 | 0,77 | 0,99 | 0 | 0,04 | POSTMENOPAUSAL |  | |  |
| rs11022778 | ARNTL | BREAST | 2 | 1,07 | 0,95 | 1,2 | 0 | 0,29 | POSTMENOPAUSAL |  | |  |
| rs11071587 | RORA | BREAST | 2 | 0,97 | 0,86 | 1,09 | 0 | 0,6 | POSTMENOPAUSAL |  | |  |
| rs11071588 | RORA | BREAST | 2 | 1,04 | 0,93 | 1,17 | 0 | 0,49 | POSTMENOPAUSAL |  | |  |
| rs11113179 | CRY1 | BREAST | 3 | 1,12 | 0,97 | 1,29 | 0 | 0,12 | POSTMENOPAUSAL |  | |  |
| rs11123857 | NPAS2 | BREAST | 3 | 1,04 | 0,95 | 1,15 | 0 | 0,38 | POSTMENOPAUSAL |  | |  |
| rs11171846 | TIMELESS | BREAST | 2 | 1,02 | 0,87 | 1,19 | 0 | 0,84 | POSTMENOPAUSAL |  | |  |
| rs1159814 | RORA | BREAST | 2 | 0,99 | 0,89 | 1,12 | 0 | 0,92 | POSTMENOPAUSAL |  | |  |
| rs1160694 | RORA | BREAST | 2 | 0,98 | 0,82 | 1,16 | 53 | 0,78 | POSTMENOPAUSAL |  | |  |
| rs11629597 | RORA | BREAST | 2 | 0,97 | 0,86 | 1,1 | 21 | 0,67 | POSTMENOPAUSAL |  | |  |
| rs11630018 | RORA | BREAST | 2 | 1 | 0,85 | 1,17 | 0 | 0,99 | POSTMENOPAUSAL |  | |  |
| rs11630062 | RORA | BREAST | 2 | 0,95 | 0,73 | 1,24 | 55 | 0,73 | POSTMENOPAUSAL |  | |  |
| rs11630227 | RORA | BREAST | 2 | 0,98 | 0,87 | 1,11 | 0 | 0,76 | POSTMENOPAUSAL |  | |  |
| rs11631432 | RORA | BREAST | 2 | 0,98 | 0,87 | 1,11 | 0 | 0,77 | POSTMENOPAUSAL |  | |  |
| rs11634318 | RORA | BREAST | 2 | 1,04 | 0,87 | 1,23 | 0 | 0,67 | POSTMENOPAUSAL |  | |  |
| rs11635314 | RORA | BREAST | 2 | 1,03 | 0,89 | 1,2 | 8 | 0,65 | POSTMENOPAUSAL |  | |  |
| rs11605924 | CRY2 | BREAST | 2 | 1,03 | 0,87 | 1,21 | 20 | 0,76 | POSTMENOPAUSAL |  | |  |
| rs11932595 | CLOCK | BREAST | 3 | 0,87 | 0,73 | 1,05 | 72 | 0,15 | POSTMENOPAUSAL |  | |  |
| rs12101478 | RORA | BREAST | 2 | 1,09 | 0,96 | 1,25 | 0 | 0,18 | POSTMENOPAUSAL |  | |  |
| rs12324440 | RORA | BREAST | 2 | 0,97 | 0,86 | 1,09 | 0 | 0,58 | POSTMENOPAUSAL |  | |  |
| rs12438866 | RORA | BREAST | 2 | 0,89 | 0,65 | 1,23 | 86 | 0,48 | POSTMENOPAUSAL |  | |  |
| rs12440095 | RORA | BREAST | 2 | 1 | 0,86 | 1,16 | 0 | 0,98 | POSTMENOPAUSAL |  | |  |
| rs12591650 | RORA | BREAST | 2 | 0,95 | 0,83 | 1,08 | 0 | 0,43 | POSTMENOPAUSAL |  | |  |
| rs12594972 | RORA | BREAST | 2 | 0,86 | 0,73 | 1,02 | 0 | 0,08 | POSTMENOPAUSAL |  | |  |
| rs12712083 | NPAS2 | BREAST | 2 | 0,97 | 0,74 | 1,27 | 80 | 0,85 | POSTMENOPAUSAL |  | |  |
| rs12712085 | NPAS2 | BREAST | 2 | 0,9 | 0,81 | 0,99 | 0 | 0,03 | POSTMENOPAUSAL |  | |  |
| rs12821586 | CRY1 | BREAST | 2 | 1,1 | 0,7 | 1,72 | 81 | 0,69 | POSTMENOPAUSAL |  | |  |
| rs12909890 | RORA | BREAST | 2 | 1 | 0,89 | 1,12 | 0 | 1 | POSTMENOPAUSAL |  | |  |
| rs13025524 | NPAS2 | BREAST | 2 | 0,96 | 0,82 | 1,13 | 48 | 0,63 | POSTMENOPAUSAL |  | |  |
| rs1323369 | RORB | BREAST | 2 | 0,97 | 0,77 | 1,22 | 72 | 0,77 | POSTMENOPAUSAL |  | |  |
| rs1327836 | RORB | BREAST | 2 | 0,98 | 0,85 | 1,13 | 0 | 0,76 | POSTMENOPAUSAL |  | |  |
| rs1327837 | RORB | BREAST | 2 | 0,94 | 0,84 | 1,06 | 0 | 0,31 | POSTMENOPAUSAL |  | |  |
| rs13329238 | RORA | BREAST | 2 | 1,08 | 0,93 | 1,24 | 36 | 0,33 | POSTMENOPAUSAL |  | |  |
| rs13329643 | RORA | BREAST | 2 | 0,93 | 0,8 | 1,07 | 0 | 0,31 | POSTMENOPAUSAL |  | |  |
| rs1351545 | RORA | BREAST | 2 | 0,93 | 0,83 | 1,05 | 0 | 0,23 | POSTMENOPAUSAL |  | |  |
| rs1351546 | RORA | BREAST | 2 | 0,98 | 0,87 | 1,1 | 0 | 0,72 | POSTMENOPAUSAL |  | |  |
| rs135757 | CSNK1E | BREAST | 2 | 0,89 | 0,78 | 1,02 | 0 | 0,09 | POSTMENOPAUSAL |  | |  |
| rs1369481 | NPAS2 | BREAST | 2 | 0,95 | 0,69 | 1,31 | 86 | 0,76 | POSTMENOPAUSAL |  | |  |
| rs1401417 | CRY2 | BREAST | 5 | 1,08 | 0,98 | 1,2 | 17 | 0,13 | POSTMENOPAUSAL |  | |  |
| rs1403737 | RORA | BREAST | 2 | 0,96 | 0,85 | 1,08 | 0 | 0,51 | POSTMENOPAUSAL |  | |  |
| rs1407845 | RORB | BREAST | 2 | 0,9 | 0,76 | 1,07 | 53 | 0,24 | POSTMENOPAUSAL |  | |  |
| rs1410226 | RORB | BREAST | 2 | 1,06 | 0,9 | 1,26 | 0 | 0,48 | POSTMENOPAUSAL |  | |  |
| rs1410227 | RORB | BREAST | 2 | 1 | 0,86 | 1,15 | 0 | 0,96 | POSTMENOPAUSAL |  | |  |
| rs1425287 | RORA | BREAST | 2 | 0,94 | 0,82 | 1,07 | 0 | 0,31 | POSTMENOPAUSAL |  | |  |
| rs1437543 | RORA | BREAST | 2 | 1,1 | 0,93 | 1,31 | 49 | 0,27 | POSTMENOPAUSAL |  | |  |
| rs1437547 | RORA | BREAST | 2 | 0,97 | 0,71 | 1,33 | 72 | 0,87 | POSTMENOPAUSAL |  | |  |
| rs1437550 | RORA | BREAST | 2 | 0,97 | 0,85 | 1,12 | 0 | 0,69 | POSTMENOPAUSAL |  | |  |
| rs1465812 | RORA | BREAST | 2 | 1 | 0,88 | 1,12 | 0 | 0,94 | POSTMENOPAUSAL |  | |  |
| rs1482049 | RORA | BREAST | 2 | 1 | 0,86 | 1,17 | 29 | 0,99 | POSTMENOPAUSAL |  | |  |
| rs1482058 | RORA | BREAST | 2 | 1,01 | 0,89 | 1,14 | 0 | 0,87 | POSTMENOPAUSAL |  | |  |
| rs1534891 | CSNK1E | BREAST | 2 | 1 | 0,86 | 1,16 | 0 | 0,97 | POSTMENOPAUSAL |  | |  |
| rs1542178 | NPAS2 | BREAST | 2 | 0,93 | 0,83 | 1,05 | 0 | 0,25 | POSTMENOPAUSAL |  | |  |
| rs1542179 | NPAS2 | BREAST | 2 | 0,88 | 0,78 | 0,99 | 0 | 0,04 | POSTMENOPAUSAL |  | |  |
| rs1589702 | RORA | BREAST | 2 | 0,96 | 0,78 | 1,18 | 67 | 0,7 | POSTMENOPAUSAL |  | |  |
| rs1589703 | RORA | BREAST | 2 | 1 | 0,88 | 1,14 | 20 | 0,98 | POSTMENOPAUSAL |  | |  |
| rs1632660 | RORA | BREAST | 2 | 0,9 | 0,8 | 1,03 | 0 | 0,12 | POSTMENOPAUSAL |  | |  |
| rs16943087 | RORA | BREAST | 2 | 1,06 | 0,92 | 1,22 | 0 | 0,44 | POSTMENOPAUSAL |  | |  |
| rs16943318 | RORA | BREAST | 2 | 0,99 | 0,88 | 1,13 | 0 | 0,92 | POSTMENOPAUSAL |  | |  |
| rs16943453 | RORA | BREAST | 2 | 1 | 0,87 | 1,15 | 0 | 1 | POSTMENOPAUSAL |  | |  |
| rs16943579 | RORA | BREAST | 2 | 0,85 | 0,7 | 1,02 | 0 | 0,08 | POSTMENOPAUSAL |  | |  |
| rs17024869 | NPAS2 | BREAST | 2 | 0,77 | 0,6 | 0,98 | 54 | 0,04 | POSTMENOPAUSAL |  | |  |
| rs17024926 | NPAS2 | BREAST | 2 | 1,09 | 0,97 | 1,23 | 0 | 0,16 | POSTMENOPAUSAL |  | |  |
| rs17191442 | RORA | BREAST | 2 | 1,11 | 0,9 | 1,38 | 39 | 0,33 | POSTMENOPAUSAL |  | |  |
| rs17191554 | RORA | BREAST | 2 | 0,97 | 0,51 | 1,84 | 91 | 0,93 | POSTMENOPAUSAL |  | |  |
| rs17204573 | RORA | BREAST | 2 | 1,01 | 0,86 | 1,19 | 0 | 0,86 | POSTMENOPAUSAL |  | |  |
| rs17204698 | RORA | BREAST | 2 | 1,06 | 0,93 | 1,21 | 0 | 0,39 | POSTMENOPAUSAL |  | |  |
| rs17204770 | RORA | BREAST | 2 | 1,05 | 0,93 | 1,18 | 0 | 0,45 | POSTMENOPAUSAL |  | |  |
| rs17237367 | RORA | BREAST | 2 | 0,97 | 0,77 | 1,23 | 69 | 0,81 | POSTMENOPAUSAL |  | |  |
| rs17237486 | RORA | BREAST | 2 | 0,98 | 0,86 | 1,12 | 0 | 0,79 | POSTMENOPAUSAL |  | |  |
| rs17237521 | RORA | BREAST | 2 | 0,91 | 0,79 | 1,05 | 0 | 0,2 | POSTMENOPAUSAL |  | |  |
| rs17237563 | RORA | BREAST | 2 | 1,05 | 0,8 | 1,37 | 71 | 0,74 | POSTMENOPAUSAL |  | |  |
| rs17270188 | RORA | BREAST | 2 | 1,13 | 0,98 | 1,31 | 32 | 0,08 | POSTMENOPAUSAL |  | |  |
| rs17270216 | RORA | BREAST | 2 | 0,92 | 0,81 | 1,05 | 0 | 0,2 | POSTMENOPAUSAL |  | |  |
| rs17303097 | RORA | BREAST | 2 | 0,91 | 0,8 | 1,04 | 0 | 0,16 | POSTMENOPAUSAL |  | |  |
| rs1902618 | RORA | BREAST | 2 | 0,97 | 0,85 | 1,11 | 0 | 0,67 | POSTMENOPAUSAL |  | |  |
| rs1916645 | RORA | BREAST | 2 | 1,06 | 0,8 | 1,4 | 68 | 0,7 | POSTMENOPAUSAL |  | |  |
| rs1997644 | CSNK1E | BREAST | 2 | 0,93 | 0,79 | 1,1 | 46 | 0,39 | POSTMENOPAUSAL |  | |  |
| rs2011857 | RORA | BREAST | 2 | 0,99 | 0,82 | 1,19 | 59 | 0,91 | POSTMENOPAUSAL |  | |  |
| rs2028122 | RORA | BREAST | 2 | 0,99 | 0,84 | 1,16 | 47 | 0,92 | POSTMENOPAUSAL |  | |  |
| rs2030619 | RORA | BREAST | 2 | 1,01 | 0,81 | 1,27 | 59 | 0,93 | POSTMENOPAUSAL |  | |  |
| rs2062094 | RORA | BREAST | 2 | 1,09 | 0,97 | 1,23 | 0 | 0,16 | POSTMENOPAUSAL |  | |  |
| rs2075984 | CSNK1E | BREAST | 2 | 1,01 | 0,89 | 1,13 | 0 | 0,93 | POSTMENOPAUSAL |  | |  |
| rs2118326 | RORA | BREAST | 2 | 0,95 | 0,84 | 1,09 | 0 | 0,48 | POSTMENOPAUSAL |  | |  |
| rs2278637 | PER1 | BREAST | 2 | 1 | 0,88 | 1,15 | 0 | 0,96 | POSTMENOPAUSAL |  | |  |
| rs228642 | PER3 | BREAST | 2 | 1,05 | 0,89 | 1,23 | 48 | 0,59 | POSTMENOPAUSAL |  | |  |
| rs228682 | PER3 | BREAST | 2 | 0,97 | 0,86 | 1,09 | 0 | 0,61 | POSTMENOPAUSAL |  | |  |
| rs228729 | PER3 | BREAST | 2 | 0,98 | 0,87 | 1,1 | 0 | 0,72 | POSTMENOPAUSAL |  | |  |
| rs2291738 | TIMELESS | BREAST | 2 | 0,99 | 0,88 | 1,11 | 0 | 0,88 | POSTMENOPAUSAL |  | |  |
| rs2292912 | CRY2 | BREAST | 2 | 0,97 | 0,7 | 1,33 | 74 | 0,84 | POSTMENOPAUSAL |  | |  |
| rs2304673 | PER2 | BREAST | 2 | 1,04 | 0,9 | 1,2 | 0 | 0,57 | POSTMENOPAUSAL |  | |  |
| rs2304674 | PER2 | BREAST | 2 | 1 | 0,89 | 1,13 | 0 | 0,95 | POSTMENOPAUSAL |  | |  |
| rs2305160 | NPAS2 | BREAST | 4 | 0,98 | 0,9 | 1,07 | 0 | 0,72 | POSTMENOPAUSAL |  | |  |
| rs2306502 | RORA | BREAST | 2 | 1,05 | 0,87 | 1,25 | 0 | 0,62 | POSTMENOPAUSAL |  | |  |
| rs2414686 | RORA | BREAST | 2 | 0,98 | 0,87 | 1,1 | 0 | 0,71 | POSTMENOPAUSAL |  | |  |
| rs2414687 | RORA | BREAST | 2 | 1,01 | 0,89 | 1,15 | 16 | 0,84 | POSTMENOPAUSAL |  | |  |
| rs2689352 | RORA | BREAST | 2 | 1 | 0,77 | 1,31 | 80 | 0,97 | POSTMENOPAUSAL |  | |  |
| rs2899664 | RORA | BREAST | 2 | 0,97 | 0,86 | 1,09 | 0 | 0,58 | POSTMENOPAUSAL |  | |  |
| rs2899666 | RORA | BREAST | 2 | 1,14 | 1 | 1,3 | 22 | 0,06 | POSTMENOPAUSAL |  | |  |
| rs3027188 | PER1 | BREAST | 2 | 0,9 | 0,72 | 1,13 | 50 | 0,37 | POSTMENOPAUSAL |  | |  |
| rs339972 | RORA | BREAST | 2 | 1,04 | 0,85 | 1,28 | 62 | 0,68 | POSTMENOPAUSAL |  | |  |
| rs339996 | RORA | BREAST | 2 | 0,97 | 0,87 | 1,09 | 0 | 0,65 | POSTMENOPAUSAL |  | |  |
| rs339998 | RORA | BREAST | 2 | 1,04 | 0,92 | 1,17 | 0 | 0,51 | POSTMENOPAUSAL |  | |  |
| rs340023 | RORA | BREAST | 2 | 0,99 | 0,88 | 1,12 | 0 | 0,86 | POSTMENOPAUSAL |  | |  |
| rs340026 | RORA | BREAST | 2 | 1,09 | 0,95 | 1,25 | 0 | 0,22 | POSTMENOPAUSAL |  | |  |
| rs341365 | RORA | BREAST | 2 | 0,97 | 0,84 | 1,12 | 32 | 0,68 | POSTMENOPAUSAL |  | |  |
| rs341366 | RORA | BREAST | 2 | 0,93 | 0,83 | 1,05 | 0 | 0,23 | POSTMENOPAUSAL |  | |  |
| rs341382 | RORA | BREAST | 2 | 1,03 | 0,91 | 1,16 | 0 | 0,65 | POSTMENOPAUSAL |  | |  |
| rs341392 | RORA | BREAST | 2 | 1,04 | 0,9 | 1,18 | 13 | 0,61 | POSTMENOPAUSAL |  | |  |
| rs341399 | RORA | BREAST | 2 | 0,91 | 0,79 | 1,05 | 0 | 0,2 | POSTMENOPAUSAL |  | |  |
| rs341403 | RORA | BREAST | 2 | 1,04 | 0,86 | 1,25 | 57 | 0,72 | POSTMENOPAUSAL |  | |  |
| rs341459 | RORA | BREAST | 2 | 0,9 | 0,71 | 1,13 | 74 | 0,35 | POSTMENOPAUSAL |  | |  |
| rs356642 | NPAS2 | BREAST | 2 | 0,95 | 0,61 | 1,49 | 89 | 0,83 | POSTMENOPAUSAL |  | |  |
| rs356652 | NPAS2 | BREAST | 2 | 1,03 | 0,85 | 1,25 | 12 | 0,75 | POSTMENOPAUSAL |  | |  |
| rs3739008 | NPAS2 | BREAST | 2 | 1,04 | 0,82 | 1,32 | 75 | 0,74 | POSTMENOPAUSAL |  | |  |
| rs3743266 | RORA | BREAST | 2 | 0,96 | 0,85 | 1,09 | 7 | 0,56 | POSTMENOPAUSAL |  | |  |
| rs3754675 | NPAS2 | BREAST | 2 | 1,14 | 0,95 | 1,36 | 0 | 0,17 | POSTMENOPAUSAL |  | |  |
| rs3768991 | NPAS2 | BREAST | 2 | 1,05 | 0,93 | 1,18 | 0 | 0,42 | POSTMENOPAUSAL |  | |  |
| rs3789327 | ARNTL | BREAST | 2 | 1,02 | 0,89 | 1,17 | 21 | 0,78 | POSTMENOPAUSAL |  | |  |
| rs3792603 | CLOCK | BREAST | 2 | 0,97 | 0,87 | 1,08 | 0 | 0,54 | POSTMENOPAUSAL |  | |  |
| rs3816358 | ARNTL | BREAST | 2 | 1,01 | 0,86 | 1,18 | 0 | 0,93 | POSTMENOPAUSAL |  | |  |
| rs3816360 | ARNTL | BREAST | 2 | 1,06 | 0,85 | 1,32 | 66 | 0,59 | POSTMENOPAUSAL |  | |  |
| rs3820787 | NPAS2 | BREAST | 2 | 0,93 | 0,78 | 1,12 | 66 | 0,45 | POSTMENOPAUSAL |  | |  |
| rs3849381 | NPAS2 | BREAST | 2 | 0,95 | 0,84 | 1,07 | 0 | 0,39 | POSTMENOPAUSAL |  | |  |
| rs3905275 | RORA | BREAST | 2 | 0,96 | 0,78 | 1,18 | 57 | 0,71 | POSTMENOPAUSAL |  | |  |
| rs4090240 | RORB | BREAST | 2 | 1,07 | 0,95 | 1,21 | 0 | 0,28 | POSTMENOPAUSAL |  | |  |
| rs4238351 | RORA | BREAST | 2 | 1 | 0,89 | 1,12 | 0 | 0,99 | POSTMENOPAUSAL |  | |  |
| rs4594196 | RORA | BREAST | 2 | 1,03 | 0,9 | 1,18 | 0 | 0,64 | POSTMENOPAUSAL |  | |  |
| rs4757144 | ARNTL | BREAST | 2 | 0,98 | 0,87 | 1,1 | 0 | 0,76 | POSTMENOPAUSAL |  | |  |
| rs4774371 | RORA | BREAST | 2 | 1,02 | 0,9 | 1,15 | 0 | 0,81 | POSTMENOPAUSAL |  | |  |
| rs4774381 | RORA | BREAST | 2 | 1,02 | 0,9 | 1,16 | 0 | 0,77 | POSTMENOPAUSAL |  | |  |
| rs4774386 | RORA | BREAST | 2 | 0,93 | 0,81 | 1,07 | 0 | 0,34 | POSTMENOPAUSAL |  | |  |
| rs4774388 | RORA | BREAST | 2 | 0,87 | 0,76 | 1 | 0 | 0,05 | POSTMENOPAUSAL |  | |  |
| rs4775292 | RORA | BREAST | 2 | 1,09 | 0,85 | 1,39 | 77 | 0,49 | POSTMENOPAUSAL |  | |  |
| rs4775340 | RORA | BREAST | 2 | 0,94 | 0,84 | 1,06 | 0 | 0,3 | POSTMENOPAUSAL |  | |  |
| rs4775351 | RORA | BREAST | 2 | 0,97 | 0,85 | 1,11 | 0 | 0,69 | POSTMENOPAUSAL |  | |  |
| rs4775352 | RORA | BREAST | 2 | 0,95 | 0,81 | 1,12 | 0 | 0,57 | POSTMENOPAUSAL |  | |  |
| rs4775355 | RORA | BREAST | 2 | 0,87 | 0,76 | 0,98 | 0 | 0,03 | POSTMENOPAUSAL |  | |  |
| rs4775362 | RORA | BREAST | 2 | 1,02 | 0,88 | 1,18 | 26 | 0,77 | POSTMENOPAUSAL |  | |  |
| rs4775369 | RORA | BREAST | 2 | 0,99 | 0,88 | 1,11 | 0 | 0,89 | POSTMENOPAUSAL |  | |  |
| rs4775371 | RORA | BREAST | 2 | 0,96 | 0,84 | 1,09 | 0 | 0,51 | POSTMENOPAUSAL |  | |  |
| rs4851377 | *NPAS2* | BREAST | 2 | 0,96 | 0,85 | 1,08 | 0 | 0,45 | POSTMENOPAUSAL |  | |  |
| rs4851390 | NPAS2 | BREAST | 2 | 1,04 | 0,92 | 1,19 | 0 | 0,53 | POSTMENOPAUSAL |  | |  |
| rs6001093 | CSNK1E | BREAST | 2 | 1,04 | 0,91 | 1,19 | 0 | 0,54 | POSTMENOPAUSAL |  | |  |
| rs6486121 | ARNTL | BREAST | 2 | 0,96 | 0,86 | 1,08 | 0 | 0,5 | POSTMENOPAUSAL |  | |  |
| rs6494217 | RORA | BREAST | 2 | 1 | 0,89 | 1,14 | 0 | 0,95 | POSTMENOPAUSAL |  | |  |
| rs6494219 | RORA | BREAST | 2 | 0,94 | 0,84 | 1,07 | 0 | 0,35 | POSTMENOPAUSAL |  | |  |
| rs6494221 | RORA | BREAST | 2 | 1,02 | 0,89 | 1,15 | 0 | 0,82 | POSTMENOPAUSAL |  | |  |
| rs6494229 | RORA | BREAST | 2 | 1,05 | 0,93 | 1,18 | 0 | 0,44 | POSTMENOPAUSAL |  | |  |
| rs6494232 | RORA | BREAST | 2 | 0,91 | 0,72 | 1,15 | 49 | 0,45 | POSTMENOPAUSAL |  | |  |
| rs6719533 | NPAS2 | BREAST | 2 | 1,08 | 0,98 | 1,2 | 0 | 0,13 | POSTMENOPAUSAL |  | |  |
| rs7032677 | RORB | BREAST | 2 | 0,97 | 0,86 | 1,09 | 0 | 0,59 | POSTMENOPAUSAL |  | |  |
| rs7037043 | RORB | BREAST | 2 | 1 | 0,89 | 1,12 | 0 | 0,97 | POSTMENOPAUSAL |  | |  |
| rs707467 | PER3 | BREAST | 2 | 0,98 | 0,85 | 1,13 | 0 | 0,78 | POSTMENOPAUSAL |  | |  |
| rs7123390 | CRY2 | BREAST | 2 | 0,89 | 0,69 | 1,14 | 64 | 0,34 | POSTMENOPAUSAL |  | |  |
| rs7164773 | RORA | BREAST | 2 | 1,17 | 1,04 | 1,32 | 0 | 0,01 | POSTMENOPAUSAL |  | |  |
| rs7166448 | RORA | BREAST | 2 | 0,86 | 0,73 | 1,02 | 0 | 0,08 | POSTMENOPAUSAL |  | |  |
| rs7168782 | RORA | BREAST | 2 | 0,92 | 0,81 | 1,06 | 0 | 0,24 | POSTMENOPAUSAL |  | |  |
| rs7172348 | RORA | BREAST | 2 | 0,94 | 0,83 | 1,06 | 0 | 0,29 | POSTMENOPAUSAL |  | |  |
| rs7177611 | RORA | BREAST | 2 | 0,93 | 0,79 | 1,1 | 48 | 0,4 | POSTMENOPAUSAL |  | |  |
| rs7183955 | RORA | BREAST | 2 | 0,94 | 0,82 | 1,08 | 7 | 0,36 | POSTMENOPAUSAL |  | |  |
| rs726955 | RORA | BREAST | 2 | 0,94 | 0,83 | 1,06 | 0 | 0,28 | POSTMENOPAUSAL |  | |  |
| rs7297614 | CRY1 | BREAST | 2 | 0,98 | 0,87 | 1,1 | 0 | 0,72 | POSTMENOPAUSAL |  | |  |
| rs7581886 | NPAS2 | BREAST | 2 | 1,02 | 0,87 | 1,19 | 8 | 0,8 | POSTMENOPAUSAL |  | |  |
| rs7598826 | NPAS2 | BREAST | 2 | 0,94 | 0,83 | 1,05 | 0 | 0,27 | POSTMENOPAUSAL |  | |  |
| rs7602358 | PER2 | BREAST | 2 | 1,04 | 0,92 | 1,18 | 0 | 0,53 | POSTMENOPAUSAL |  | |  |
| rs7605570 | NPAS2 | BREAST | 2 | 0,95 | 0,85 | 1,06 | 0 | 0,33 | POSTMENOPAUSAL |  | |  |
| rs7698022 | CLOCK | BREAST | 2 | 0,86 | 0,7 | 1,06 | 60 | 0,15 | POSTMENOPAUSAL |  | |  |
| rs782903 | RORA | BREAST | 2 | 0,97 | 0,86 | 1,09 | 0 | 0,63 | POSTMENOPAUSAL |  | |  |
| rs782907 | RORA | BREAST | 2 | 0,93 | 0,79 | 1,1 | 49 | 0,42 | POSTMENOPAUSAL |  | |  |
| rs782910 | RORA | BREAST | 2 | 0,97 | 0,86 | 1,1 | 0 | 0,68 | POSTMENOPAUSAL |  | |  |
| rs782926 | RORA | BREAST | 2 | 1,01 | 0,88 | 1,16 | 28 | 0,87 | POSTMENOPAUSAL |  | |  |
| rs782938 | RORA | BREAST | 2 | 0,93 | 0,83 | 1,05 | 0 | 0,25 | POSTMENOPAUSAL |  | |  |
| rs782944 | RORA | BREAST | 2 | 0,92 | 0,7 | 1,19 | 79 | 0,51 | POSTMENOPAUSAL |  | |  |
| rs782947 | RORA | BREAST | 2 | 0,99 | 0,8 | 1,23 | 62 | 0,92 | POSTMENOPAUSAL |  | |  |
| rs7848922 | RORB | BREAST | 2 | 0,91 | 0,78 | 1,06 | 40 | 0,23 | POSTMENOPAUSAL |  | |  |
| rs7867494 | RORB | BREAST | 2 | 0,86 | 0,76 | 0,97 | 0 | 0,01 | POSTMENOPAUSAL |  | |  |
| rs7950226 | ARNTL | BREAST | 2 | 0,98 | 0,87 | 1,1 | 0 | 0,73 | POSTMENOPAUSAL |  | |  |
| rs8023252 | RORA | BREAST | 2 | 0,96 | 0,86 | 1,08 | 0 | 0,5 | POSTMENOPAUSAL |  | |  |
| rs8024629 | RORA | BREAST | 2 | 0,89 | 0,78 | 1,01 | 0 | 0,08 | POSTMENOPAUSAL |  | |  |
| rs8027032 | RORA | BREAST | 2 | 0,95 | 0,84 | 1,08 | 68 | 0,43 | POSTMENOPAUSAL |  | |  |
| rs8027829 | RORA | BREAST | 2 | 0,95 | 0,85 | 1,07 | 0 | 0,43 | POSTMENOPAUSAL |  | |  |
| rs8031801 | RORA | BREAST | 2 | 1,12 | 0,99 | 1,28 | 0 | 0,08 | POSTMENOPAUSAL |  | |  |
| rs8032023 | RORA | BREAST | 2 | 0,91 | 0,81 | 1,03 | 0 | 0,14 | POSTMENOPAUSAL |  | |  |
| rs8034880 | RORA | BREAST | 2 | 1,03 | 0,87 | 1,21 | 3 | 0,75 | POSTMENOPAUSAL |  | |  |
| rs8034950 | RORA | BREAST | 2 | 0,98 | 0,87 | 1,1 | 0 | 0,73 | POSTMENOPAUSAL |  | |  |
| rs8036866 | RORA | BREAST | 2 | 1,08 | 0,91 | 1,28 | 0 | 0,36 | POSTMENOPAUSAL |  | |  |
| rs8037669 | RORA | BREAST | 2 | 0,97 | 0,87 | 1,09 | 0 | 0,64 | POSTMENOPAUSAL |  | |  |
| rs8038077 | RORA | BREAST | 2 | 1,17 | 0,98 | 1,38 | 0 | 0,08 | POSTMENOPAUSAL |  | |  |
| rs8041061 | RORA | BREAST | 2 | 1 | 0,89 | 1,13 | 0 | 1 | POSTMENOPAUSAL |  | |  |
| rs8041381 | RORA | BREAST | 2 | 0,91 | 0,81 | 1,03 | 0 | 0,12 | POSTMENOPAUSAL |  | |  |
| rs8042149 | RORA | BREAST | 2 | 1 | 0,89 | 1,12 | 0 | 0,95 | POSTMENOPAUSAL |  | |  |
| rs8043356 | RORA | BREAST | 2 | 0,97 | 0,83 | 1,12 | 0 | 0,65 | POSTMENOPAUSAL |  | |  |
| rs809736 | RORA | BREAST | 2 | 0,98 | 0,86 | 1,11 | 0 | 0,71 | POSTMENOPAUSAL |  | |  |
| rs875994 | PER3 | BREAST | 2 | 1,08 | 0,95 | 1,23 | 0 | 0,26 | POSTMENOPAUSAL |  | |  |
| rs880625 | RORA | BREAST | 2 | 0,96 | 0,83 | 1,1 | 0 | 0,55 | POSTMENOPAUSAL |  | |  |
| rs880626 | RORA | BREAST | 2 | 0,98 | 0,87 | 1,11 | 0 | 0,76 | POSTMENOPAUSAL |  | |  |
| rs895520 | *NPAS2* | BREAST | 2 | 1,02 | 0,81 | 1,28 | 73 | 0,86 | POSTMENOPAUSAL |  | |  |
| rs895521 | NPAS2 | BREAST | 2 | 0,89 | 0,79 | 1,01 | 0 | 0,08 | POSTMENOPAUSAL |  | |  |
| rs919000 | RORA | BREAST | 2 | 0,97 | 0,85 | 1,11 | 0 | 0,68 | POSTMENOPAUSAL |  | |  |
| rs934945 | PER2 | BREAST | 3 | 1,06 | 0,94 | 1,19 | 10 | 0,33 | POSTMENOPAUSAL |  | |  |
| rs940222 | RORA | BREAST | 2 | 1,03 | 0,91 | 1,16 | 0 | 0,64 | POSTMENOPAUSAL |  | |  |
| rs940224 | RORA | BREAST | 2 | 1 | 0,89 | 1,13 | 0 | 0,99 | POSTMENOPAUSAL |  | |  |
| rs965519 | NPAS2 | BREAST | 2 | 0,97 | 0,8 | 1,17 | 50 | 0,74 | POSTMENOPAUSAL |  | |  |
| rs968357 | RORB | BREAST | 2 | 1,06 | 0,93 | 1,21 | 0 | 0,42 | POSTMENOPAUSAL |  | |  |
| rs969485 | ARNTL | BREAST | 2 | 0,95 | 0,85 | 1,07 | 0 | 0,44 | POSTMENOPAUSAL |  | |  |
| rs974828 | RORA | BREAST | 2 | 0,92 | 0,78 | 1,09 | 0 | 0,35 | POSTMENOPAUSAL |  | |  |
| rs9788704 | RORA | BREAST | 2 | 1,01 | 0,81 | 1,26 | 72 | 0,93 | POSTMENOPAUSAL |  | |  |
| rs9806453 | RORA | BREAST | 2 | 1,05 | 0,93 | 1,18 | 0 | 0,42 | POSTMENOPAUSAL |  | |  |
| rs9920560 | RORA | BREAST | 2 | 1,08 | 0,93 | 1,25 | 0 | 0,29 | POSTMENOPAUSAL |  | |  |

| **PROSTATE CANCER SUBGROUP** | | | | | | | | | | | |
| --- | --- | --- | --- | --- | --- | --- | --- | --- | --- | --- | --- |
| **SNP ID** | **GENE** | **CANCER** | **STUDIES** | **OR** | **CI Lower** | **CI Upper** | **I ^2^ %** | **P value** | **Cases** | **Controls** |  |
| rs1005473 | CSNK1E | PROSTATE | 2 | 1,09 | 0,61 | 1,96 | 31 | 0,76 | 1446 | 2480 |  |
| rs1012477 | PER3 | PROSTATE | 2 | 1,15 | 0,97 | 1,37 | 21 | 0,12 | 1446 | 2480 |  |
| rs10462020 | PER3 | PROSTATE | 2 | 1,04 | 0,91 | 1,19 | 0 | 0,55 | 1446 | 2480 |  |
| rs11121023 | PER3 | PROSTATE | 2 | 0,99 | 0,87 | 1,12 | 0 | 0,82 | 1446 | 2480 |  |
| rs12315175 | CRY1 | PROSTATE | 2 | 1,1 | 0,97 | 1,25 | 0 | 0,14 | 1446 | 2480 |  |
| rs135757 | CSNK1E | PROSTATE | 2 | 0,99 | 0,88 | 1,11 | 0 | 0,88 | 1446 | 2480 |  |
| rs1369481 | NPAS2 | PROSTATE | 2 | 0,99 | 0,7 | 1,41 | 79 | 0,97 | 1446 | 2480 |  |
| rs1401417 | CRY2 | PROSTATE | 2 | 0,78 | 0,58 | 1,03 | 31 | 0,08 | 1446 | 2480 |  |
| rs1534891 | CSNK1E | PROSTATE | 2 | 0,99 | 0,85 | 1,14 | 0 | 0,85 | 1446 | 2480 |  |
| rs1562313 | NPAS2 | PROSTATE | 2 | 0,94 | 0,83 | 1,06 | 0 | 0,32 | 1446 | 2480 |  |
| rs17024926 | NPAS2 | PROSTATE | 2 | 1,1 | 0,96 | 1,27 | 24 | 0,18 | 1446 | 2480 |  |
| rs228697 | PER3 | PROSTATE | 2 | 1,11 | 0,94 | 1,32 | 0 | 0,21 | 1446 | 2480 |  |
| rs2289591 | PER1 | PROSTATE | 2 | 1,07 | 0,95 | 1,2 | 0 | 0,3 | 1446 | 2480 |  |
| rs2292910 | CRY2 | PROSTATE | 2 | 1,03 | 0,92 | 1,15 | 0 | 0,61 | 1446 | 2480 |  |
| rs2305160 | NPAS2 | PROSTATE | 3 | 1,02 | 0,92 | 1,13 | 0 | 0,75 | 1446 | 2480 |  |
| rs2640909 | PER3 | PROSTATE | 2 | 1,1 | 0,93 | 1,3 | 34 | 0,27 | 1446 | 2480 |  |
| rs6001093 | CSNK1E | PROSTATE | 2 | 0,99 | 0,87 | 1,13 | 0 | 0,92 | 1446 | 2480 |  |
| rs7297614 | CRY1 | PROSTATE | 2 | 0,92 | 0,82 | 1,04 | 10 | 0,19 | 1446 | 2480 |  |
| rs7602358 | PER2 | PROSTATE | 2 | 1,09 | 0,96 | 1,23 | 0 | 0,17 | 1446 | 2480 |  |
| rs774027 | TIMELESS | PROSTATE | 2 | 1,03 | 0,93 | 1,14 | 0 | 0,57 | 1446 | 2480 |  |
| rs774047 | TIMELESS | PROSTATE | 2 | 1,03 | 0,93 | 1,14 | 0 | 0,59 | 1446 | 2480 |  |
| rs7950226 | ARNTL | PROSTATE | 2 | 1,18 | 0,96 | 1,45 | 36 | 0,12 | 1446 | 2480 |  |
| rs885747 | PER1 | PROSTATE | 2 | 1,03 | 0,85 | 1,25 | 55 | 0,75 | 1446 | 2480 |  |
| rs895521 | NPAS2 | PROSTATE | 2 | 0,98 | 0,68 | 1,41 | 79 | 0,93 | 1446 | 2480 |  |

LEGEND: CI 95% confidence interval ; OR summary Odds Ratio
